# Trial-based economic evaluations of non-drug interventions in the Royal Australian College of General Practitioners (RACGP) Handbook of Non-Drug Interventions in primary care: a systemic review

**DOI:** 10.1136/fmch-2025-003312

**Published:** 2025-08-31

**Authors:** Tiffany Atkins, Darryn Marks, Caroline Dowsett, Paul Glasziou, Loai Albarqouni

**Affiliations:** 1 Institute for Evidence-Based Healthcare, Bond University, Robina, Queensland, Australia; 2 Faculty of Medicine and Health Sciences, Bond University, Gold Coast, QLD, Australia

**Keywords:** General Practice, Diet, Food, and Nutrition, Family Medicine

## Abstract

**Objective:**

This systematic review assessed trial-based economic evaluations to provide empirical evidence on the cost-effectiveness of non-drug interventions (NDIs) that are currently recommended within the Royal Australian College of General Practitioners Handbook of Non-Drug Interventions (HANDI).

**Methods:**

Medline, CINAHL and PsycINFO along with clinical trial registries (clinicaltrials.gov and WHO International Clinical Trials Registry Platform) were searched from inception to 1 July 2025. Randomised controlled trials (RCTs) that reported cost effectiveness for a prescribed non-drug intervention (NDI) from HANDI were included in the study. The primary outcome was the incremental cost-utility ratio (ICUR) derived from cost-utility analyses (CUAs).

**Results:**

A total of 11 187 citations were identified, from which 156 RCTs were included. These RCTs enrolled a total of 66 926 participants (median=214, IQR 139–342), with a median follow-up duration of 12 months (IQR 6–12 months). Over half of the CUA NDIs were for mental health conditions (n=81; 54.0%), one-third were for were for musculoskeletal conditions (n=44; 29.3%), while only 16.0% (n=24) were for those with cardiovascular/metabolic conditions. Out of the 150 NDIs that reported CUAs, 40% were deemed to be in the south-east (SE) quadrant (cheaper and more effective) and 49.3% fell in the north-east (NE) quadrant (more costly but more effective), with 70% considered cost effective against a £25 000/quality-adjusted life-year (QALY) willingness to pay threshold. The overall median ICUR was £2400/QALY (IQR −18 986 to 20 027).

**Conclusions:**

Most of the HANDI NDIs that were included within this systematic review are cost-effective compared with a variety of alternatives including usual care or waiting list controls. HANDI NDIs warrant use as a first line of treatment when clinically appropriate.

**WHAT IS ALREADY KNOWN ON THIS TOPIC**

- The Royal Australian College of General Practitioners’ Handbook of Non-Drug Interventions (HANDI) recommends many clinically effective non-drug interventions (NDIs) for use in primary care.
- However, a comprehensive analysis of the cost-effectiveness of these recommended NDIs was lacking, creating a barrier to their widespread adoption and funding.

**WHAT THIS STUDY ADDS**

- This systematic review of 156 trials demonstrates that most NDIs recommended by HANDI are cost-effective, with 40% being dominant (cheaper and more effective) and an additional 49.3% providing good value for money compared with usual care.
- The review provides specific cost-effectiveness data across major conditions, finding that 86% of musculoskeletal, 67% of cardiovascular/diabetes and 64% of mental health NDIs are cost-effective.

**HOW THIS STUDY MIGHT AFFECT RESEARCH, PRACTICE OR POLICY**

- This study provides the economic evidence for clinicians to confidently prescribe and for policymakers to fund, a wide range of NDIs as efficient, first-line treatments in primary care.
- By identifying the few interventions that were not cost-effective, this review directs future research towards improving their value or developing more affordable alternatives.

## Introduction

 Effective non-drug interventions (NDIs) have been recommended to prevent and treat various conditions^1 2^ including heart disease, low back pain, insomnia and anxiety.^1^,^6^ NDIs encompass a broad spectrum of interventions, including but not limited to nutritional/dietary, psychological and behavioural, physical therapies (including exercise), devices and mobile applications interventions.^7 8^ Some NDIs have been shown to be equivalent, or even superior, in preventing, managing and treating common conditions in primary care.^1 2 9^ For instance, cognitive behavioural therapy (CBT) has demonstrated comparable efficacy to antidepressants in the management of depression.^10^ In 2013, the Royal Australian College of General Practitioners (RACGP) developed the Handbook of Non-Drug Interventions (HANDI) to promote effective, evidence-based NDIs in primary care.^11^

While the effectiveness of NDIs is well-documented, their economic viability remains a critical factor for widespread adoption. NDIs must not only be effective but also affordable to ensure wider adoption and scalability.^12^ Health economic evaluations measure the value of an intervention, in the context of the money spent to achieve the beneficial outcome.^13^ This is of importance for clinical stakeholders, policymakers and wider society to ensure that scarce healthcare resources are used cost-effectively to produce in the pursuit of beneficial healthcare outcomes.^13^

While the existing literature contains numerous focused systematic reviews evaluating the cost-effectiveness of single NDIs for specific conditions, a high-level synthesis that assesses the economic evidence across the full spectrum of guideline-recommended interventions is currently lacking. Therefore, our scope was intentionally broad to reflect the comprehensive nature of the RACGP HANDI guidelines. This approach allows us to assess the collective economic impact of recommended NDIs as a whole, a perspective particularly useful for health systems and insurers evaluating the viability of non-pharmacological care models.

The cost-effectiveness of NDI prescription in primary care is not known. This systematic review aims to synthesise and critically evaluate the economic evaluations of NDIs within primary care to inform guidance or recommendations for practice, healthcare policy and further research. Specifically, we will focus on trial-based economic evaluations to provide empirical evidence on the cost-effectiveness of NDIs, currently recommended in the RACGP HANDI.

## Methods

### Design

We conducted a systematic review of trial-based economic analyses of NDIs included in the RACGP HANDI. We reported this review according to the Preferred Reporting Items for Systematic Reviews and Meta-Analyses statement.^14^ We developed a protocol prospectively and registered it on Open Science Framework (10.17605/OSF.IO/CBQKN).

### Information sources and search strategy

We searched three databases (Medline, CINAHL and PsycINFO) for the available peer-reviewed literature from inception to 5 June 2023, which was updated again on 1 July 2025. We also searched clinical trial registries (clinicaltrials.gov and WHO International Clinical Trials Registry Platform, ICTRP). We used a suite of tools from within the systematic review accelerator (SRA) website^15^ to assist with the search and screening. We used the *word frequency analysis* tool to establish key terms and synonyms^16^ and to assist in the development of the search strategy. We designed the search strategy with the help of a senior information specialist, using both key terms and Medical Subject Heading terms about NDIs, cost effectiveness and randomised controlled trials (RCTs). We used the *Polyglot Search* Translator tool^15^ to translate the search strategy for use with other electronic databases.^16^ The complete search strings for all databases are provided in online supplemental table 1 There were no language or publication date restrictions. We also conducted a forward and backward citation analysis (11 March 2024) of all included studies using the *SpiderCite* tool.^15^

### Eligibility criteria

To be eligible, studies must base their cost-effectiveness analysis on data from RCTs; hence, we excluded studies that based their economic analysis on data from other types of studies or economic models. We included RCTs that enrolled patients of any age, sex or ethnicity to evaluate the cost-effectiveness of NDIs, currently recommended by HANDI (https://www.racgp.org.au/clinical-resources/clinical-guidelines/handi), within a primary care setting. We excluded studies that were conducted in hospitals (online supplemental table 2).

The primary outcome was the incremental cost-utility ratio (ICUR) as part of a cost-utility analysis (CUA) using both the incremental costs and incremental quality-adjusted life-years (QALYs) for the interventions. This is a specific type of incremental cost-effectiveness ratio (ICER). ICERs are a standardised measure that allows for comparison given that analysis will include different interventions and different health outcomes.^17^ ICERs are considered the gold standard for cost-effectiveness measures.^17 18^ Many studies referred to the two ratios interchangeably, especially if they only used ICUR as their main unit of analysis.

Secondary outcomes were ICERs based on incremental costs and incremental health effects (severity of depression, % improvement in anxiety, % those recovered, # of falls prevented, etc) as part of a cost effectiveness analysis (CEA) or net benefit (NB) as part of a cost benefit analysis (CBA).

### Study screening and selection

The titles and abstracts of studies were screened by two reviewers (CD and LA). One review author (CD) retrieved full-texts of potentially eligible records, and the same author (CD) screened the full-texts for inclusion. A random sample of articles (10%) was screened by a second author (LA). To help with the screening process, we used the tools from the SRA such as the *Screenatron* tool to keep track of includes and excludes while we screened the title and abstract articles, and we used the *Disputatron* tool to help identify disputes between the two authors.^15^

### Data extraction

A standardised form (initially piloted on two included studies) was used for data extraction of characteristics of studies and outcomes. We extracted data items in accordance with the items identified by the Consolidated Health Economic Evaluation Reposting Standards checklist.^19^ One author (CD or TA) extracted the following data from included studies, which was then checked by a second author (LA) for accuracy, and conflicts were resolved through discussion. Extracted data items include:

- *Study characteristics*: study authors, year, location, study setting, time and study duration.
- Participants: number of participants and inclusion/exclusion criteria and health condition.
- *Interventions and comparators:* HANDI intervention type, intervention description, type of comparator and comparator description.
- *Outcomes:* economic perspective (societal/health), type of analysis (CUA, CEA or CBA), outcome measures (eg, incremental costs, incremental QALYs, incremental health effects and ICUR/ICER), currency and price year and the discounted rate if used.

### Economic analysis

To standardise the economic data across various studies, we used a web-based tool, CCEMG-EPPI Centre Cost Converter,^20^ to convert the estimates of the incremental costs of interventions from their original currency and price year to the same currency and price year (ie, the 2023 £ using the International Monetary Fund source dataset to calculate the Purchasing Power Parities values as part of the conversion calculation). For studies that reported an ICER or ICUR as ‘Dominated’ or ‘Dominates’ or did not report these metrics, we derived the ICER/ICUR by calculating the ratio of the mean incremental costs to the mean incremental health effects or QALYs as provided by the study.

We graphically presented the standardised point estimates of ICURs on an incremental cost-effectiveness plane which is divided into four quadrants and centred at 0 (online supplemental figure 1); cost-effectiveness plane). The y axis being incremental costs and the x axis being incremental effectiveness measured in QALYs. The *south-east (SE)* quadrant represented studies which were considered most ideal and had HANDI interventions that were less costly and had increased QALYs compared with the comparator (more effective). The *north-aast (NE) quadrant* contained studies that had HANDI interventions that were more costly but had increased QALYs (more effective). The *south-west (SW) quadrant* contained studies that had interventions that were less costly but lowered QALYs (less effective). Finally, the *north-west (NW)* contained studies that had interventions that were more expensive but lowered QALYs (less effective), which would be considered least ideal. We created separate graphical plots for studies adopting solely a societal perspective and those employing a health system perspective. A conservative willingness to pay threshold of £25 000/QALY was applied.^21^,^23^

Owing to the heterogeneity of comparators and the presence of combinations of drug and non-drug usual care comparators in some studies, it was not possible to clearly delineate between these groups. We therefore reported the results of included studies in four summary tables, each corresponding to one of the quadrants based on ICURs, summarising the interventions from all studies within each quadrant. We also displayed the cost-effectiveness quadrant for each condition and treatment group. Studies featuring multiple interventions were represented in more than one quadrant table. Information about the graphing details of the intervention is also within each quadrant table (perspective, intervention and comparator, ICUR using costs in £ (2023) and incremental costs in £ (2023)). For studies that provided both societal and health system perspective data, both sets of details were included in the relevant quadrant table. Additionally, a fifth table summarised studies that reported only secondary outcomes based on CEA and NB from aCBA.

No meta-analyses were performed; hence, we did not formally assess levels of heterogeneity, publication bias or small study effects. We did not contact investigators or study sponsors to provide missing or incomplete data or clarification of data contents.

### Patient and public involvement

No patients or members of the public were involved in the design, conduct, reporting or dissemination plans of this review.

## Results

### Search results

**Figure 1:**
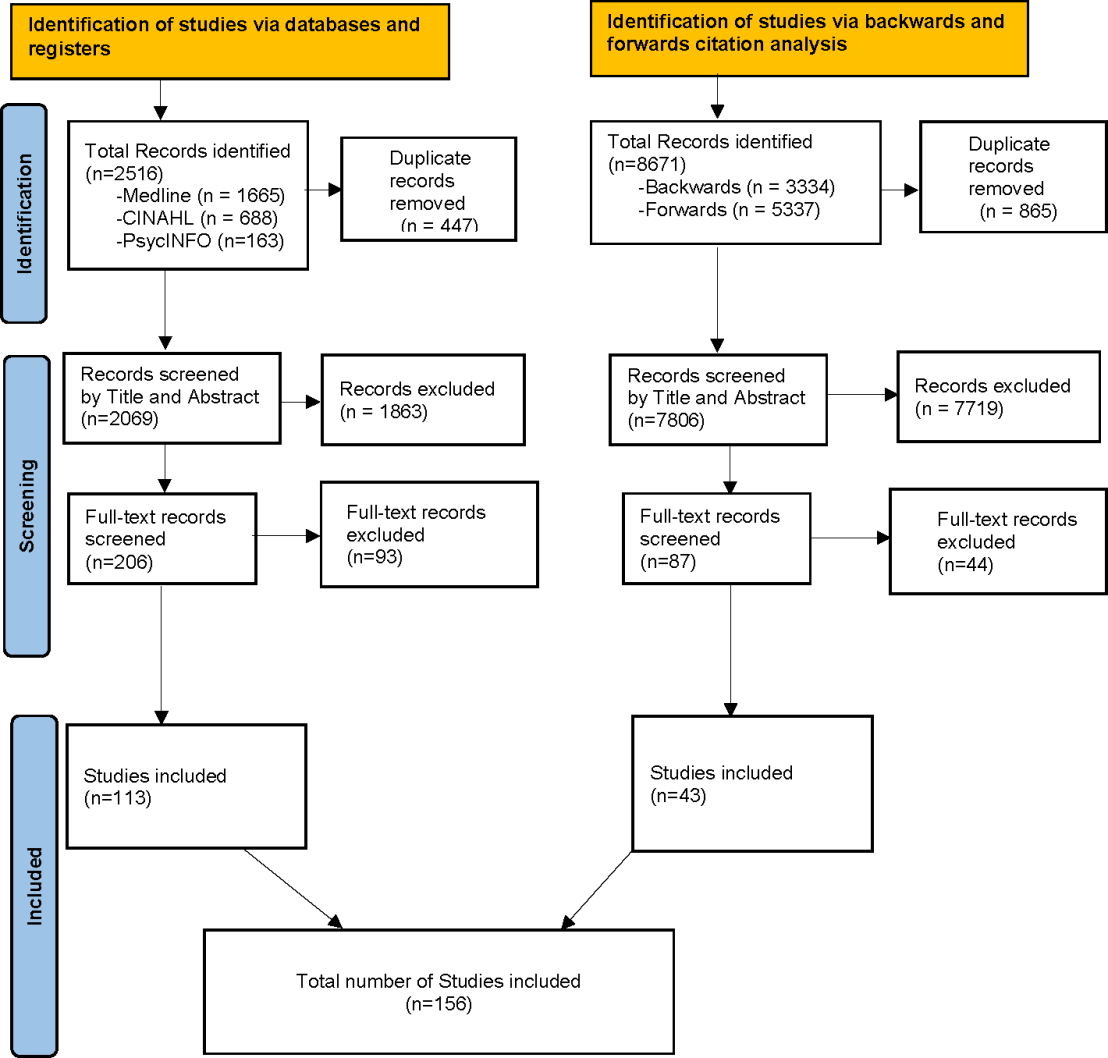
Preferred Reporting Items for Systematic Reviews and Meta-Analyses flow diagram.

A total of 11 187 citations were identified from across all databases, registries and citation analysis, of which 1312 were removed as duplicates. Of the 9875 unique citations, 9582 were excluded after titles and abstracts screening and 137 were excluded after the full-text screening (excluded citations with reasons for exclusions available at online supplemental tables 3 and 4). Overall, a total of 156 studies were included in this review (figure 1).

### Characteristics of included studies

A total of 156 included studies evaluated 176 eligible NDIs. These studies enrolled a total of 66 926 participants (median=214, IQR 139–342), with a median follow-up duration of 12 months (IQR 6–12 months). Most of the included studies were conducted in Europe (69.9%) with 37.2% (n=58) using the € as a currency and 80.8% (n=126) of the studies were conducted from 2010 onwards. Over half of the included studies evaluated behavioural interventions such as internet or computerised CBT (n=83; 53.2%). Exercise-based interventions were the next most common (n=62; 39.7%). Just under 3% of studies had interventions related to nutrition and dietary interventions (n=4, 2.6%). Over half of the studies were also within the mental health category (n=86, 55.1%) while only four studies (2.6%) were part of the diabetes category. Most studies (n=66, 42.3 %) completed the analysis from a societal perspective. The included studies comprised 68 studies (43.6 %, 79 interventions) that conducted both a CEA and a CUA, 62 studies (39.7 %, 71 interventions) that conducted only a CUA, 25 studies (16.0 %, 25 interventions) that conducted only a CEA and one study that strictly conducted a CBA. Table 1 below outlines a summary for overall number of studies and interventions. Additional summary characteristics for overall number of studies and interventions are provided in online supplemental table 5.

**Table 1:** Summary of characteristics of total included studies and total interventions including both CUA and CEA interventions

	Total studies n=156 n (% of total studies)	Total interventions n=176 n (% of total interventions)	CUA interventions n=150 n (% of total interventions)	CEA interventions n=25 + 1 CBA n (% of total interventions)
Treatment				
Behavioural	83 (53.2)	94 (53.4)	79 (52.7)	15 (8.5)
Exercise/physical therapy	62 (39.7)	70 (39.8)	60 (40.0)	10 (5.7)
Exercise+behavioural	1 (0.6)	1 (0.6)	1 (0.7)	0 (0.0)
Device*	5 (3.2)	6 (3.4)	6 (4.0)	0 (0.0)
Nutrition	4 (2.6)	4 (2.3)	3 (2.0)	1 (0.6)
Other	1 (0.6)	1 (5.7)	1 (0.7)	0 (0.0)
Category				
Mental health	86 (55.1)	96 (54.5)	81 (54.0)	15 (8.5)
Musculoskeletal	45 (28.8)	54 (30.7)	44 (29.3)	10 (5.7)
Cardiovascular	20 (12.8)	21 (11.9)	21 (14.0)	0 (0.0)
Diabetes	4 (2.6)	4 (2.3)	3 (2.0)	1 (0.6)
Children	1 (0.6)	1 (0.6)	1 (0.7)	0 (0.0)
Perspective				
Societal	66 (42.3)	79 (44.9)	68 (45.3)	11 (6.3)
Health System	61 (39.1)	72 (40.9)	60 (40.0)	12 (6.8)
Gave both societal and health	23 (14.7)	19 (10.8)	19 (12.7)	0 (0.0)
Payer/employer	6 (3.8)	6 (3.4)	3 (2.0)	3 (1.7)
Currency				
€	58 (37.2)	69 (39.2)	60 (40.0)	9 (5.1)
£	46 (29.5)	51 (29.0)	44 (29.3)	7 (4.0)
US$	30 (19.2)	33 (18.8)	28 (18.7)	5 (2.8)
$C/$A/NZD	20 (12.8)	21 (11.9)	16 (10.7)	5 (2.8)
Other; ¥**/**SGD	2 (1.3)	2 (1.1)	2 (1.3)	0 (0.0)
Region				
Europe	109 (69.9)	15 (8.5)	109 (72.7)	16 (9.1)
North America	31 (19.9)	33 (18.8)	26 (17.3)	7 (4.0)
South America	1 (0.6)	2 (1.1)	2 (1.3)	0 (0.0)
Asia	4 (2.6)	5 (2.8)	5 (3.3)	0 (0.0)
Oceania	11 (7.1)	11 (6.3)	8 (5.3)	3 (1.7)
Type of analysis				
CUA only	62 (39.7)	71 (40.3)	71 (47.3)	0 (0.0)
CUA and CEA	68 (43.6)	79 (44.9)	79 (52.7)	0 (0.0)
Only CEA/CBA	26 (16.7)	26 (14.8)		26 (14.8)

* Device refers to a type of intervention that uses a specific device such as a splint, walking cane, pedometer or mandibular device.

CBA, cost 269 benefit analysis; CUA, cost utility analysis; NZD, New Zealand Dollar; SGD, Singapore Dollar.

### Primary outcome: CUA

A total of 150 interventions and 130 studies reported cost utility analyses and calculated ICURs. These have been summarised by the ICUR quadrant (table 2) and have also been graphically presented in online supplemental figure 2) (health system perspective) and online supplemental figure 3) (societal perspective). The 150 NDIs fell into six different treatment types. Most of the 150 identified interventions (134/150; 89.3%) were clustered in two quadrants of either the dominant SE quadrant (40%; n=60) which cost less and are more effective (higher QALYs) or the NE quadrant (49.3%; n=74) which costs more and are more effective (higher QALYs) compared with the reference or standard intervention (table 2; online supplemental tables 7 and 8). Only 9 (6%) of the 150 CUA interventions found that NDIs cost less and were less effective (table 2; online supplemental table 9). Only 7 out of the 150 CUA interventions (4.7 %) found NDIs cost more and were less effective (table 2; online supplemental table 10).

**Table 2:** Summary of 150 CUA interventions across 130 studies by ICUR quadrant

n=130 total CUA studies and 150 CUA interventions¶				
Total of CUA interventions N (column %) of total CUA intervention	Q 1 * n (row %)	Q 2† n (row %)	Q 3 ‡ n (row %)	Q 4§ n (row %)
Treatment n=150 (100.0)	n=60 (40%)	n=74 (49.3)	n=9 (6.0%)	n=7 (4.7)
Behavioural n=79 (52.7)	33 (41.8)	33 (41.8)	8 (10.1)	5 (6.3)
Exercise/physical therapy n=60 (40.0)	23 (38.3)	34 (56.7)	1 (1.7)	2 (3.3)
Exercise+behavioural n=1 (0.7)	0 (0.0)	1 (100.0)	0 (0.0)	0 (0.0)
Devices** n=6 (4.0)	2 (33.3)	4 (66.7)	0 (0.0)	0 (0.0)
Nutrition n=3 (2.0)	2 (66.7)	1 (33.3)	0 (0.0)	0 (0.0)
Other n=1 (0.7)	0 (0.0)	1 (100.0)	0 (0.0)	0 (0.0)
Category n=150 (100.0)	n=60 (40%)	n=74 (49.3)	n=9 (6.0%)	n=7 (4.7)
Mental health n=81 (54.0)	32 (39.5)	36 (44.4)	8 (9.9)	5 (6.2)
Musculoskeletal n=44 (29.3)	19 (43.2)	23 (52.3)	0 (0.0)	2 (4.5)
Cardiovascular n=21 (14.0)	8 (38.1)	12 (57.1)	1 (4.8)	0 (0.0)
Diabetes n=3 (2.0)	1 (33.3)	2 (66.7)	0 (0.0)	0 (0.0)
Children n=1 (0.7)	0 (0.0)	1 (100.0)	0 (0.0)	0 (0.0)

* Q1=interventions that cost less and are more effective

† Q2=interventions that cost more and are more effective

‡ Q3=interventions that cost less and are less effective

§ Q4 interventions that cost more and are less effective.

¶ Study may have more than one intervention but only used one

** Device refers to a type of intervention that uses a specific device such as a splint, walking cane, pedometer or mandibular device.

CUA, cost utility analysis; ICUR, incremental cost utility ratio.

Of interventions in the NE quadrant, 45/74 (60.8%) are cost effective against the £25 000 willingness to pay (WTP). Overall, 70.7% (106/150) of all interventions are deemed cost-effective against the £25 000 WTP. The overall median ICUR was £2400/QALY (IQR −18986 to 20027). Societal perspective studies reported more in the dominant SE quadrant compared with the studies from a health system perspective (online supplemental figures 2 and 3). Additional characteristics of CUA interventions by ICUR quadrant are summarised in online supplemental table 6. Reported ICUR values by quadrant appear in online supplemental tables 7–10.

**Figure 2:**
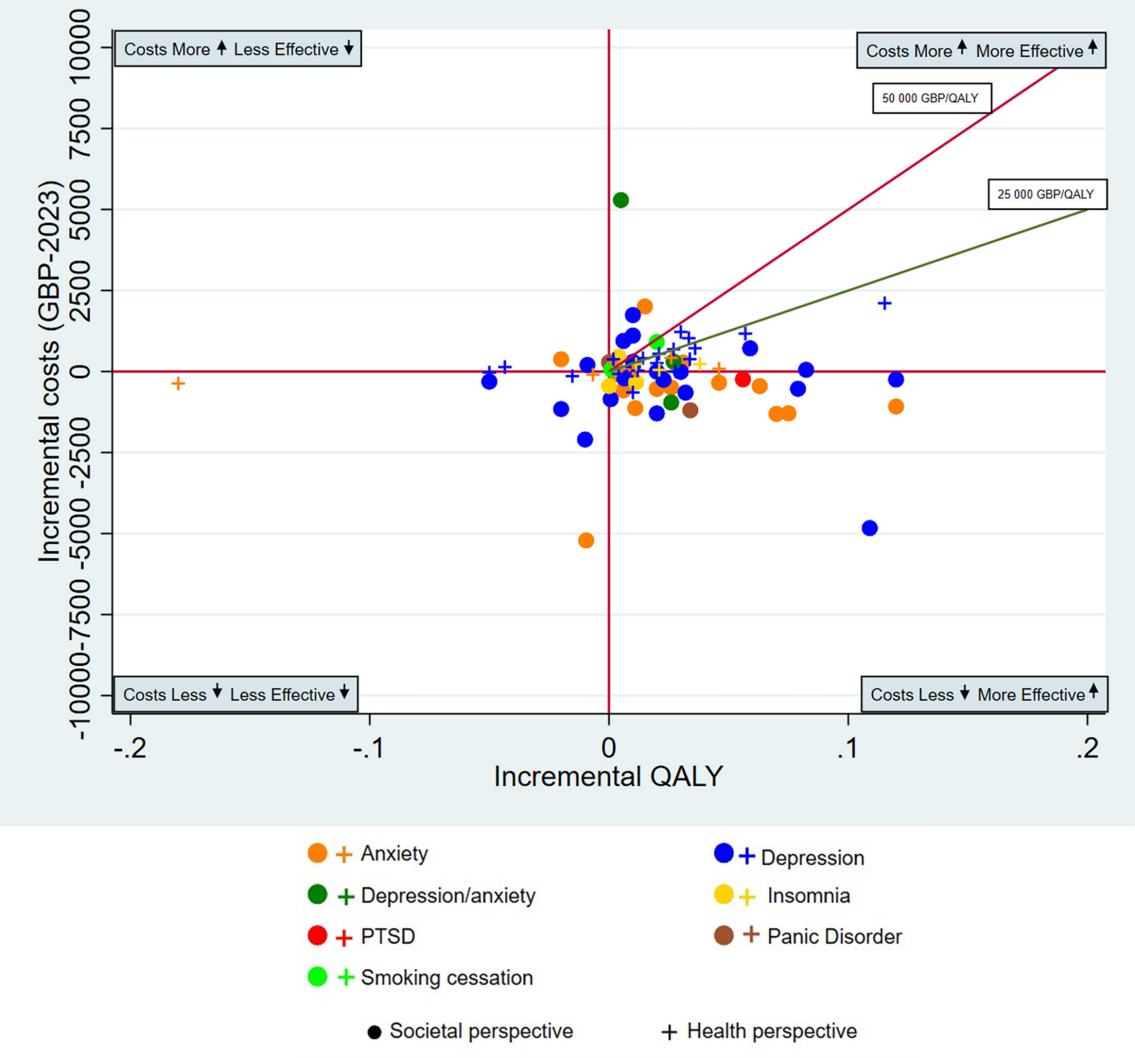
Cost utility analysis of the interventions according to Handbook of Non-Drug Interventions, by mental health conditions and economic perspective. The outlier study by Barnhofer *et al*^31^ is not shown. GBP, British pound sterling; QALY, quality-adjusted life-year; PTSD, post-traumatic stress disorder.

A summary of studies by disease condition was also completed below (tables3^5^) and displayed graphically (figures2  3 and online supplemental figure 4). Detailed results of interventions by quadrant with references are detailed within the (online supplemental material 1) which include online supplemental tables 12–15.

**Table 3:** Summary of CUA non-drug interventions (NDIs) for mental health conditions (n=81 interventions)

Condition	*Interventions	Type of intervention	Details of interventions
Interventions that cost less and are more effective (SE quadrant) (n=32 interventions)			
Health anxiety	3	Behavioural	Internet CBT (unguided) versus control † Internet CBT with therapist support versus control † or+UC versus UC †
Social anxiety disorder (SAD)	3	Behavioural	Internet CBT with therapist support versus group F2F CBT † or versus IDST † Text-based CBT (StudiCare) versus waitlist control †
Anxiety	5	Behavioural	Internet CBT with therapist support versus internet delivered child play †, or UC † School based CBT versus school classroom clinician care † Brief parent delivered CBT versus solution-focussed brief therapy † BACT versus BI CBT †
Depression	11	Behavioural	CBT versus usual GP care ‡, CBT versus antidepressant ‡, 1 day CBT workshop versus WLC †, + UC versus UC †, internet CBT with no therapist support+UGPC versus UGPC † or versus WLC †‡, computer assisted CBT versus F2F CBT †, web-based CDMIs versus UC ‡†, mindfulness CBT versus TAU†
	1	Exercise/physical therapy	Mindful yoga+TAU versus TAU†
Anxiety and/or depression	4	Behavioural	CBT versus UGPC †, BBT versus ARC †, computerised CBT (beating the blues) + UGPC versus UGPC †, MBCT-SH versus CBT-SH ‡
Insomnia	3	Behavioural	Internet-guided CBT versus TAU †, internet CBT with therapist versus group F2F CBT †
Panic disorder	1	Behavioural	Individual CBT with therapist versus UGPC †
PTSD	1	Behavioural	Prolonged exposure therapy versus pharmacotherapy with sertraline †
Interventions that cost more but are more effective (NE quadrant) (n=36 interventions)			
Health anxiety	2	Behavioural	Bibliotherapy CBT versus WLC†*‡, internet CBT (unguided) versus WLC‡ or internet CBT (guided) versus WLC †*‡, internet CBT with therapist support versus behavioural stress management †
SAD	4	Behavioural	CBT with therapist versus WLC †*, internet CBT with therapist support versus group F2F CBT or versus IDST‡ Text-based CBT (StudiCare) versus WLC‡
Anxiety	2	Behavioural	Transdiagnostic CBT group therapy+TAU versus TAU‡† Internet CBT with therapist support versus WLC‡
Depression	16	Behavioural	CBT versus UGPC‡, CBT versus CR‡, Internet CBT versus TAU*†, internet CBT guided by nurse’s vs ODF‡ Internet CBT with own therapist+UGPC versus UGPC‡*, internet CBT with therapist support vs UGPC†*, or versus WLC‡,†*, Group F2F CBT versus UGPC†, Preventative Cognitive therapy (supported self-help) versus UC‡†*, Internet CBT+problem solving therapy versus Enhanced UC‡†, Internet problem-solving therapy versus WLC+self help book*†, unguided internet CBT versus WLC ‡, computer-assisted CBT versus TAU*‡
	4	Exercise/physical therapy	Physical exercise+TAU versus TAU‡*, physical exercise versus TAU‡†* supervised walking programme versus UGPC‡, preferred intensity exercise+TAU versus TAU*‡
Anxiety and/or depression	2	Behavioural	CBT versus UGPC†, concise care (SSRI or CBT up to only 7 weeks) versus standard care*†
Insomnia	3	Behavioural	Internet guided CBT versus TAU‡, internet CBT with therapist support+UGPC versus UGPC+WLC*, CBT workshop versus WLC†*
Smoking cessation	2	Behavioural	Video-based computer tailored programme versus control†*, computer tailored programme+counselling versus UC†*
Interventions that cost less but were less effective (n=8 interventions)			
Health anxiety	1	Behavioural	Internet CBT (self-help with therapist support) versus individual F2F CBT†*
SAD	1	Behavioural	Internet CBT (with therapist support) versus group F2F CBT ‡
Anxiety	2	Behavioural	Online parent-led CBT (with support) versus TAU ‡, school-based CBT versus school clinician care †
Depression	4	Behavioural	Internet CBT with therapist support versus UGPC ‡† CBT with therapist versus fluoxetine *, computerised CBT (MoodGym) ‡ (Colour your life) † versus UC*
Interventions that cost more and are less effective (n=5 interventions)			
Anxiety	1	Behavioural	Family CBT versus individual CBT†
Depression	2	Behavioural	Computerised CBT (Beating the Blues) ‡ versus UC, classroom-based CBT versus usual school curriculum delivered by teachers †
Smoking cessation	2	Behavioural	Internet-based multiple computer-tailored programme versus UC †*, text-based computer-tailored programme versus control †*

* Not cost effective based on mean ICUR>£25 000/QALY

† Societal perspective

‡ Health system perspective

ARC, assisted referral to community outpatient mental healthcare; BACT, blended acceptance and commitment therapy; BBT, brief behavioural therapy; BI, brief individual; BSC, best supportive care; CBT, cognitive behavioural therapy; CBT-SH, cognitive behavioural therapy-self-help; CDMI, complaint-directed min-interventions; F2F, face-to-face; GP, general practitioner; ICUR, incremental cost utility ratio; IDST, internet delivered supportive therapy; MBCT-SH, mindfulness-based cognitive therapy-self-help; ODF, online discussion forum; SPR, standard physical rehabilitation; TAU, treatment as usual; UC, usual care; UGPC, usual GP care; WLC, wait-list contro.

**Table 4:** Summary of CUA NDIs for musculoskeletal conditions (n=44 interventions)

Condition	*Interventions	Type of intervention	Details of interventions
Interventions that cost less and are more effective (SE quadrant) (n=19 interventions)			
Low back pain	2	Behavioural	CBT+SPR versus SPR †, CBT (exposure in vivo) versus graded activity †
	4	Exercise/physical therapy	Yoga versus UC‡ or waitlist control†, Pilates once per week versus control (booklet) †, exercise programme versus control †
Hip and/or knee OA/PFPS	8	Exercise/physical therapy	Exercise versus UC ‡†, group water-based exercise vs control †, F2F physio+web app versus usual physio †‡, class-based exercise programme+home-based versus Home-based ‡, supervised exercise programme versus UC†, strength exercise versus UC ‡†, aerobic exercise versus UC†
Fall prevention	4	Exercise/physical therapy	Otago exercise programme+UC versus UC ‡, Resistance exercise 1×, or 2× per week versus balance and tone ‡, Tai J Quan versus stretching‡
CFS	1	Exercise/physical therapy	Graded exercise therapy (GET) versus specialist medical care (SMC) †
Interventions that cost more but are more effective (NE quadrant) (n=23 interventions)			
Low back pain	1	Behavioural	Group F2F CBT+UGPC versus UGPC†
	6	Exercise/physical therapy	Yoga versus UC‡ or waitlist control‡, multimodal therapy (exercise training+behavioural support) versus TAU §, E-exercise (smartphone app integrated into F2F physio) versus F2F physio‡†, Pilates 2×/Pilates 3× per week versus control (booklet) †‡, BOOST programme versus BPA
	1	Exercise+behavioural	Group exercise+Group F2F CBT versus Usual GP care‡
Hip and/or knee OA/knee pain	7	Exercise/physical therapy	Strengthening exercise versus standard care (leaflet),‡* supervised exercise therapy with pain coping skills versus just pain coping skills†, Hand exercises versus control‡, aquatic classes verssus control†, exercise physiotherapy versus usual GP care†‡, Physical therapy versus glucocorticoid injections‡, aerobic exercise versus UC‡
OA	2	Device	Splint versus SM‡*, knee brace+UC versus UC†
Fall prevention	4	Exercise/physical therapy	E-health exercise programme+UC versus UC‡*, Otago exercise programme+UC versus UC (‡women), multifactorial fall prevention programme versus UC*‡, physiotherapy (Exergames) versus SC (leaflet) ‡
CFS	1	Exercise/physical therapy	GET versus SMC ‡
Various	1	Exercise/physical therapy	National Exercise Referral scheme versus UC‡
Interventions that cost less but were less effective (n=0 interventions)			
Interventions that cost more and are less effective (n=2 interventions)			
Knee OA	1	Exercise/physical therapy	Individually tailored or targeted exercise adherence versus usual physical therapy‡*
Chronic low back pain	1	Exercise/physical therapy	Active physical therapy+graded activity with problem solving versus graded activity with problem solving†*

* Not cost effective based on mean ICUR>£25 000/QALY

† Societal perspective

‡ Health system perspective

§ Payer perspective

BSC, best supportive care; GP, general practitioner; NDI, non-drug intervention; SPR, standard physical rehabilitation; TAU, treatment as usual.

**Table 5:** Summary of CUA non-drug interventions (NDIs) for cardiovascular/diabetes conditions (n=24 interventions)

Condition	*Interventions	Type of intervention	Details of interventions
Interventions that cost less and are more effective *(SE quadrant)* (n=9 interventions)			
CAD with or without ACS	3	Exercise/physical therapy	Internet-based tele-rehab versus usual rehab†, exercise-based cardiac rehab versus usual GP Care‡, CRPP (exercise+education) versus control (no exercise)*
Stroke	1	Exercise/physical therapy	Exercise programme versus control (balance and tone) ‡
Chronic obstructive pulmonary disease	1	Exercise/physical therapy	Home-based exercise rehab+usual GP care versus usual GP care‡†
High blood pressure prevention	1	Nutrition	Salt substitute versus control (regular salt) †u
OSA/OSAHS	2	Device	CPAP+BSC versus BSC‡, boil and bite MAD or patient moulded MAD versus control *
Type 2 diabetes	1	Nutrition	Weight management (Counterweight Plus) + TAU versus TAU ‡
Interventions that cost more but are more effective (NE quadrant) (n=14 interventions)			
CD or CVD	2	Exercise/physical therapy	Progressively autonomous physical activity versus standard supervised physical activity‡, Lifestyle modification programme+UC versus UC†
Heart Failure	3	Exercise/physical therapy	Rehabilitation group versus control‡* Supervised centre-based exercise training+disease management programme (UC) versus UC‡* Exercise training (HF-ACTION)+usual GP care versus usual GP care†*
Intermittent claudication	2	Exercise/physical therapy	Home-based structured exercise versus walk advice, supervised exercise therapy versus unsupervised walking advice†*
Stroke	1	Exercise/physical therapy	High Intensity exercise versus conventional physical therapy‡
Stroke prevention	1	Nutrition	Salt substitute versus control (regular salt) ‡
COPD	1	Exercise/physical therapy	Exercise+education versus TAU‡*
OSA/OSAHS	2	Device	MAD versus CPAP†* Bespoke MAD versus control‡*
Type 2 diabetes	2	Exercise/physical therapy	WBV-based exercise therapy+TAU versus TAU‡, resistance exercise versus WTC* and aerobic exercise versus WTC†
Interventions that cost less but were less effective (n=1 interventions)			
Intermittent claudication	1	Exercise/physical therapy	Supervised exercise therapy versus endovascular revascularisation †
Interventions that cost more and are less effective (n=0 interventions)			

* Not cost effective based on mean ICUR>£25 000/QALY

† Societal perspective

‡ Health system perspective

ACS, acute coronary syndrome; CAD, coronary artery disease; CD, coronary disease; CPAP, continuous positive airway pressure; CVD, cardiovascular disease; F2F, face-to-face; MAD, mandibular advancement device; OSA, obstructive sleep apnoea; OSAHS, obstructive sleep apnoea-hypopnea syndrome; TAU, treatment as usual; UC, usual care; WBV, whole body vibration; WTC, waitlist control.

**Figure 3:**
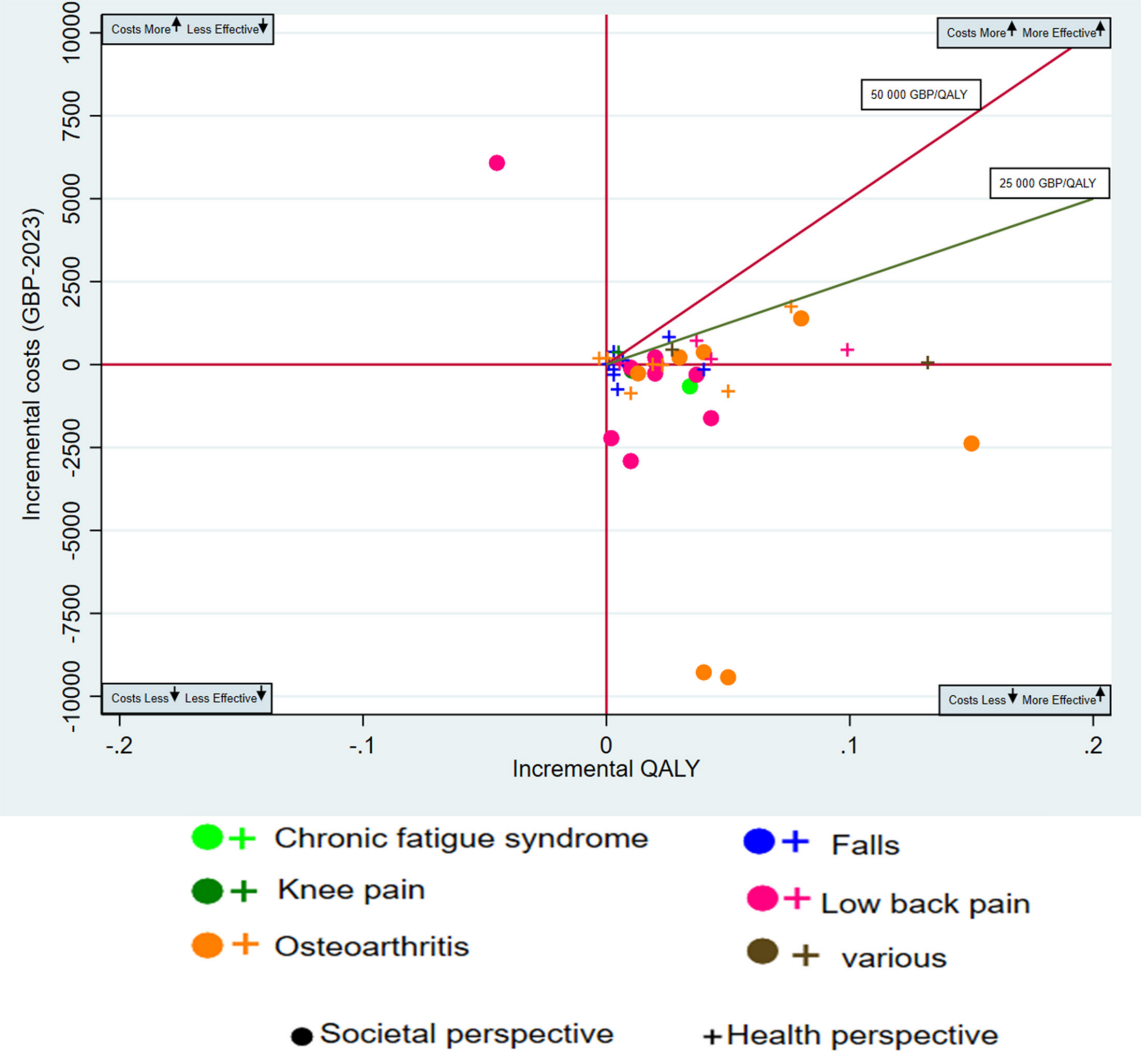
Cost utility analysis of the interventions according to Handbook of Non-Drug Interventions, by musculoskeletal conditions and perspective. The outlier study by Patrick *et al*^32^ is not shown. GBP, British pound sterling; QALY, quality-adjusted life-year.

### Distribution of HANDI interventions (NDIs) across disease conditions

#### Mental health conditions

*Overall, 64% of non-drug interventions for mental health conditions were cost-effective*. There were a total of 81 NDIs for mental conditions (figure 2, table 3). This represents 54% of the total CUA NDIs (81/150). There were NDIs for seven different mental health conditions: health anxiety, social anxiety disorder, anxiety, depression, insomnia, panic disorder, post-traumatic stress disorder, along with those for smoking cessation. Of these, 32 interventions (32/81=39.5%) fell in the dominant most favourable SE quadrant that cost less and are more effective (higher QALYs). There were 36 interventions (44.4%) that cost more but were more effective (NE quadrant), eight interventions (9.9%) that cost less but were less effective and five interventions (6.2%) that cost more and were less effective. Overall, approximately 64% (52/81) of NDIs for mental health conditions were considered cost effective at a WTP threshold of £25 000/QALY. The median ICUR for mental health conditions was £2669/QALY (IQR −18 672 to 21 293). Most interventions for mental health conditions were behavioural (76/81=93.8%), while only five out of the 81 (6.2%) were exercise/physical therapy interventions. Table 3 and figure 2 below outline the CUA interventions for mental health conditions by condition and economic perspective (societal or health). Most behavioural interventions for mental health conditions were a form of CBT. All four NDIs included for smoking cessation were not found to be cost effective (mean ICUR>£25 000/QALY). Many exercise interventions for depression (3 out of 5) were also not found to be cost effective. This included ‘12 supervised exercise sessions using the patient’s preferred exercise intensity’ for those aged 14–17 years with depression.^24^ Although mindful yoga versus treatment as usual and a supervised walking programme versus usual GP care were both deemed cost effective (mean ICUR<£25 000/QALY) for depression.

#### Musculoskeletal conditions

*Overall, 86% of non-drug interventions for musculoskeletal conditions were cost effective*. A total of 44 out of the 150 CUA NDIs (29%) were for musculoskeletal conditions (figure 3, table 4). The interventions for musculoskeletal conditions spanned five different conditions (low back pain, hip and/or knee osteoarthritis (OA), falls prevention, chronic fatigue syndrome and various other conditions).

Of these NDIs for musculoskeletal conditions, 19 interventions (43.2%) fell into the most favourable quadrant (SE) that cost less and were more effective, 23 (52.3%) cost more but were more effective (NE quadrant), none (0%) cost less and were less effective and two (5%) cost more and were less effective. Most interventions for musculoskeletal conditions were exercise/physical therapy interventions (38/44=86.3%), three were behavioural, two were devices and one combined exercise with a behavioural intervention (CBT). Overall, 38 out of the 44 NDIs (86.4%) for musculoskeletal conditions are considered to be cost effective at a WTP of £25 000/QALY. Only six different interventions for musculoskeletal conditions were not considered cost effective. Two of these were exercises for falls, one was a splint for thumb OA compared with self-management, strengthening exercises for knee pain, an individually tailored exercise for knee OA and physical therapy for low back pain. The median ICUR for musculoskeletal conditions was £447/QALY (IQR −28 990 to 10 085). Table 4 and figure 3 below display the CUA of HANDI interventions for musculoskeletal conditions for each condition and by economic perspective.

#### Cardiovascular/diabetes conditions

*Overall, 67% of non-drug interventions for cardiovascular/diabetes conditions were cost-effective table 5, online supplemental figure 4*. There were a total of 24 CUA NDIs (16%) that were for cardiovascular or diabetes conditions. Of these 24 interventions, nine (37.5%) fell into the most favourable SE quadrant of those that cost less and are more effective, 14 (58%) were interventions that cost more but were more effective (NE quadrant), one (4%) cost less but was less effective and none (0%) cost more and were less effective. Most of these interventions (17/24=70.8%) were exercise/physical therapy interventions such as exercise. Others included devices (4/24=16.7%) and nutritional interventions (3/24=12.5%). Interventions in this category spanned conditions such as stroke (including stroke prevention), high blood pressure, chronic obstructive pulmonary disease (COPD), coronary artery disease with or without acute coronary syndrome, obstructive sleep apnoea (OSA)/OSA-hypopnoea syndrome (OSAHS), heart failure, intermittent claudication and type 2 diabetes. Overall, 16 out of the 24 NDIs (66.7%) for cardiovascular/diabetes conditions were considered cost-effective above the WTP threshold of £25 000/QALY.

Only eight NDIs for cardiovascular/diabetes conditions were not considered cost effective against the WTP threshold. All three exercise programmes/rehab or training interventions for those with heart failure were not considered cost-effective. The supervised exercise therapy compared with unsupervised walking advice for those with intermittent claudication was also not deemed cost-effective. For those with OSA, a mandibular advancement device (MAD) compared with continuous positive airway pressure and for those with OSAHS, the Bespoke MAD device versus control was not cost effective. Additionally, for those with COPD, an exercise and education intervention compared with control was not deemed cost-effective, along with resistance exercise versus waitlist control for those with type 2 diabetes. The median ICUR for cardiovascular/diabetes conditions was £4140/QALY (IQR −16 066 to 35087). See table 5 below and online supplemental figure 4, which displays the cardiovascular/diabetes CUA interventions by condition and economic perspective.

#### Children’s conditions

There was only one intervention^25^ out of the 150 CUA interventions (0.7%) that fell into the children’s condition category. This intervention involved applying daily emollients for the first year to prevent eczema in children at risk of the condition compared with just the standard advice (control). This intervention fell into the NE quadrant and thus cost more than the control but resulted in a higher quality of life (more effective). Unfortunately, this intervention did not meet the WTP threshold of at least £25 000/ QALY. The ICUR, which was from a health economic perspective, was quite high above this threshold at £92 176/QALY.

## Secondary analysis: CEA

(See online supplemental table 11 and Secondary Analysis: Cost-effectiveness (CEA) in the online supplemental material 1).

## Discussion

This is the first systematic review to specifically evaluate the health economic impact of NDIs included in the RACGP HANDI. All NDIs appearing within the HANDI have demonstrated clinical efficacy.^11^ This review demonstrates that these interventions are also generally cost-effective with 40% of reported ICURs in the dominant SE quadrant of the cost-effectiveness plane, indicating they were both cheaper and more effective than alternatives. Furthermore, 49.3% of reported ICURs fell in the NE quadrant, with approximately 60.8% of these cost effective when assessed at the WTP threshold of £25 000. Overall, 89% of all interventions either fell in the dominant SE quadrant or the second most favourable NE quadrant, and 70% were considered cost effective at the WTP threshold of £25 000. In the majority of studies, NDIs were compared with usual care (which often involved drugs) or waiting list controls. The very few reported ICURs finding HANDI interventions to be in the SW (cost less and less effective) quadrant or the NW (cost more and less effective) quadrant of the cost effectiveness plane, related to studies that compared multiple NDIs (such as comparing different forms of CBT) or with interventions that included some NDI. Interestingly, there were also a few commonalities within those NDIs that were not cost-effective. All four NDI’s that were for smoking cessation were not cost effective. Many exercise/physical therapy interventions for those with depression (3 out of the 5) were also below the WTP threshold. All three NDIs (exercise/rehab/training programmes) for those with heart failure were also not considered cost-effective at the WTP threshold of £25 000/QALY. Non-drug interventions for musculoskeletal conditions had the highest percentage (86%) that were deemed cost-effective compared with the proportion of NDIs that were cost effective for mental health conditions (64%) and cardiovascular/diabetes conditions (67%).

This review highlights that a clear majority of included studies reported NDIs to be more effective than their comparator with regards to quality of life. This finding is both expected and consistent with previous reviews into the clinical efficacy of NDIs measured with a variety of condition-specific outcomes.^1^,^3^ With respect to the cost-effectiveness of non-pharmacological interventions in primary care, no prior reviews exist and, furthermore, limited review data in comparable fields are available for comparison. A lack of cost-effectiveness data has been cited with relation to the non-pharmacological management of hypertension,^26^ while NDIs appear to be cost effective in reducing dementia-related nursing home admissions.^27^ Given the importance of cost and cost-effectiveness to the value proposition posed by NDIs, it is imperative that more research be undertaken in this area, so that the societal benefits and policy impacts may be fully appreciated. This review demonstrates that clinically efficacious NDIs within the HANDI are also cost-effective. Primary care providers and policy makers should therefore consider interventions within the HANDI as both effective and efficient, first-line treatment options when clinically appropriate, in many situations spanning cardiovascular and musculoskeletal disease, diabetes and mental health.

The forward-backward citation analysis proved to be a useful supplemental component of our strategy, identifying an additional 43 articles for inclusion that were not retrieved through the initial database search. Our experience aligns with evidence demonstrating that citation searching is a highly effective supplementary method for enhancing evidence retrieval, especially for a broad review topic such as ours—often identifying relevant studies missed by keyword-based searches alone.^28^,^30^

## Limitations

Our review is limited to the mean ICUR base-case results of each intervention and does not synthesise decision uncertainty, a choice made due to our broad scope and the inconsistent reporting in the source material.

Across the 156 included studies with a total of 176 interventions, the degree of heterogeneity of interventions and of comparators precluded meta-analysis. More targeted systematic review/s with narrow inclusion criteria with respect to intervention, control and outcome are required to better quantify analysis of pooled data. Some included studies compared different types of NDIs, and in others with a usual care comparator, sometimes usual care included non-drug components. These accounted for most of the small number of studies that reported less favourable ICURs. Exclusion of these studies may have resulted in a purer comparison of drug versus NDIs. In this first synthesis of the literature in this field, we chose to include these studies to provide a fuller synthesis of the HANDI cost-effectiveness literature.

A necessary limitation of our broad mapping approach is the inherent trade-off between scope and clinical specificity. Consequently, while our findings provide a valuable overview for policymakers and researchers by identifying evidence gaps, they are not intended to provide granular cost-effectiveness estimates to directly inform clinical decisions for specific patient subgroups or conditions. Such recommendations would require dedicated, focused systematic reviews and our work helps to highlight where these are most urgently needed.

## Conclusion

HANDI NDIs are generally cost-effective compared with a variety of alternatives such as usual care or waiting list controls, with more than two-thirds showing cost effectiveness below a WTP threshold of £25 000/QALY, with four out of 10 NDIs showing dominance. This finding is consistent across all non-drug treatment categories and condition categories. HANDI NDIs warrant use as first line treatment when clinically appropriate.

## Supplementary material

10.1136/fmch-2025-003312 online supplemental file 1
