# Supplementary material for: Trial-based economic evaluations of non-drug interventions in the Royal Australian College of General Practitioners (RACGP) Handbook of Non-Drug Interventions in primary care: a systemic review
Source: Fam Med Community Health. 2025 Aug 31;13(3):e003312. doi: 10.1136/fmch-2025-003312 (PMC12406924; doi:10.1136/fmch-2025-003312)
Supplement: online supplemental file 1 [file fmch-13-3-s001.docx]

**Title:** A Systematic Review of Trial-Based Economic Evaluations of Non-Drug Interventions (NDIs) in the Handbook of Non-Drug Interventions (HANDI) in Primary care

**Supplementary materials**

- **Supplementary Table 1.** Search strings
- **Supplementary Table 2** Inclusion and exclusion criteria
- **Supplementary Table 3** List of excluded studies from original search with reasons for exclusion (n = 86)
- **Supplementary Table 4** List of excluded studies from backwards/forwards citation analysis with reasons for exclusion (n = 44)
- **Supplementary Figure 1-** Cost effectiveness plane
- **Supplementary Table 5** –Additional characteristics of total included studies and total interventions
- **Supplementary Table 6-** Additional characteristics of CUA interventions by ICUR quadrant
- **Supplementary Figure 2-** Cost utility analysis of HANDI interventions based on the Health System perspective
- **Supplementary Figure 3-** Cost utility analysis of HANDI interventions based on the Societal perspective
- **Supplementary Table 7.** Characteristics of studies and CUA interventions that cost less and are more effective (60 interventions)
- **Supplementary Table 8**. Characteristics of studies and CUA interventions that cost more but are more effective (74 interventions)
- **Supplementary Table 9**. Characteristics of studies and CUA interventions that cost less and are less effective (9 interventions)
- **Supplementary Table 10**. Characteristics of studies and CUA interventions that cost more and less effective (7 interventions)
- **Supplementary Table 11**. Characteristics of studies only completing cost effectiveness Analyses (CEA) using ICER’s (25 CEA) + 1 that used CBA
- **Supplementary Figure 4-** Cost utility analysis of HANDI interventions by cardiovascular/diabetes conditions and by economic perspective
- **Supplementary Material 1**: Detailed results with references for interventions by quadrant (**with Supplementary Tables 12-15**)
- **-**Primary Outcome- CUA
- **-**Secondary Outcomes-CEA

| **Supplementary Table 1. Search strings MEDLINE (Ovid)**  ("Non-Drug Intervention".tw. OR "Non-drug interventions".tw. OR NDI.tw. OR "Non-Drug Therap*".tw. OR "Non-pharmacological treatment".tw. OR "non-pharmacological therapy".tw.)  OR  ("Mothers Kiss".tw. AND (exp "foreign body"/ OR "Nasal foreign body".tw. OR "foreign body, nose".tw. OR exp "Nasal cavity"/))  OR  ((Autoinflation.tw. OR exp insufflation/ OR exp "Valsalva Maneuver"/) AND ("glue ear".tw. OR grommets.tw. OR exp "Otitis Media with Effusion"/ OR exp "Acoustic Impedance Tests"/ OR exp "Eustachian Tube"/ OR exp "Hearing Loss"/))  OR  (("Apple juice".tw. OR exp "Fruit and Vegetable Juices"/) AND (rehydration.tw. OR exp "rehydration Solutions"/ OR exp dehydration/ OR exp electrolytes/ OR exp "Fluid Therapy"/ OR exp "Gastroenteritis"/))  OR  (("pulled elbow".tw. OR exp "elbow injuries"/ OR exp radius/) AND (exp "Manipulation, Orthopedic"/ OR sublaxation.tw. OR "radial head".tw. OR exp "Sprains and Strains"/ OR exp supination/))  OR  ((exp "pain management"/ OR exp pain/ OR exp "acute pain"/ OR exp psychotherapy/ OR "combined modality theory".tw.) AND (exp immunization/ OR Immunisation.tw. OR exp Vaccination/ OR exp injections/ OR exp Needles/) AND (children.tw. OR exp child/ OR exp Infant/ OR exp "infant care"/))  OR  ((exp "pain management"/ OR exp pain/ OR exp "acute pain"/ OR exp psychotherapy/ OR "combined modality theory".tw.) AND (exp immunization/ OR Immunisation.tw. OR exp Vaccination/ OR exp injections/ OR exp Needles/) AND (children.tw. OR exp child/ OR exp Infant/ OR exp "infant care"/))  OR  ((alarms.tw. OR exp "clinical alarms"/ OR exp "Cognitive Behavioral Therapy"/) AND (bedwetting.tw. OR exp "nocturnal enuresis"/ OR exp enuresis/) AND (exp child/ OR Children.tw.))  OR  (("infant sleep".tw. OR "sleep programs".tw. OR exp "Behavior therapy"/ OR exp "infant care"/) AND ("maternal mood".tw. OR "maternal mental health".tw. OR exp "Depression, Postpartum"/ OR exp "Mother-Child Relations"/ OR exp Mothers/ OR exp "Mental Health"/))  OR  (("early exposure".tw. OR "early introduction".tw.) AND ("allergenic food*".tw. OR allergy.tw. OR egg.tw. OR peanut.tw.) AND (prevention.tw. OR "allergy prevention".tw.) AND (exp child/ OR exp infant/))  OR  ((reflux.tw. OR "infant reflux".tw. OR exp "gastroesophageal reflux"/) AND ("feed thickener".tw. OR exp "Food Additives"/ OR exp "infant formula"/))  OR  ((exp eczema/ OR "atopic eczema".tw. OR exp "Dermatitis, atopic"/) AND (Moisturiser.tw. OR exp probiotics/ OR exp lactobacillus/) AND ("high-risk infants".tw. OR exp infants/ OR exp pregnancy/ OR exp "prenatal care"/))  OR  (("wet combing".tw. OR conditioner.tw.) AND ("head lice".tw. OR exp pediculus/ OR exp "Scalp Dermatoses"/ OR exp "lice infestations"/ OR exp Pediculus/))  OR  ((exp honey/) AND (exp cough/ OR exp "Respiratory Tract Infections"/ OR URTI.tw. OR "upper respiratory tract infection".tw. OR exp "antitussive agents"/) AND (exp child/ OR children.tw.))  OR  ((exp exercise/ OR exp "exercise therapy"/ OR "exercise rehabilitation".tw.) AND (exp "Heart failure"/ OR "heart failure symptoms".tw. OR exp "cardiac rehabilitation"/))  OR  ((exp exercise/ OR exp "exercise therapy"/ OR exp walking/) AND (exp "Peripheral Arterial Disease"/ OR exp "intermittent claudication"/ OR claudication.tw.))  OR  ((exp "Valsalva Maneuver"/ OR "modified valsalva".tw.) AND (exp "Tachycardia, Supraventricular"/ OR SVT.tw. OR "sinus rhythm".tw.))  OR  (("fitness training".tw. OR exp exercise/ OR "aerobic exercise".tw. OR exp "resistance training"/ OR combined aerobic and resistance training.tw. OR exp "activities of daily living"/ OR exp "exercise therapy"/ OR exp "Physical fitness"/) AND (exp stroke/ OR "cerebrovascular accident".tw. OR CVA.tw. OR exp "Stroke rehabilitation"/))  OR  ((exp "diet, Mediterranean"/ OR exp "diet, fat-restricted"/) AND (exp "Cardiovascular diseases"/ OR "Coronary Disease".tw. OR "CVD".tw. OR "Cardiovascular disease risk".tw. OR exp "Myocardial infarction"/))  OR  ((exp "preoperative exercise"/ OR "respiratory pre-rehabilitation".tw. OR "preoperative inspiratory muscle training".tw. OR exp "Breathing exercises"/ OR exp "Preoperative care"/) AND (exp Surgery/ OR "before surgery".tw. OR "Surgical procedure".tw. OR exp "Cardiac surgical procedure"/ OR exp "Elective Surgical Procedures"/) AND ("Postoperative pneumonia".tw. OR exp "postoperative complications"/ OR exp "pulmonary atelectasis"/))  OR  ((exp Lung/ OR pulmonary.tw.) AND (exp Rehabilitation/ OR rehab.tw. OR exp "Exercise Tolerance"/) AND (exp "Pulmonary Disease, Chronic Obstructive"/ OR COPD.tw.))  OR  ((exp "Sleep Apnea, Obstructive"/ OR "Obstructive sleep apnoea".tw. OR exp "Disorders of Excessive Somnolence"/) AND (exp "Continuous Positive Airway Pressure"/ OR exp "Positive-Pressure Respiration"/ OR exp "Occlusal Splints"/ OR CPAP.tw. OR "Mandibular Splint".tw. OR exp "Mandibular Advancement"/))  OR  ((exp "Diabetes Mellitus"/ OR Diabetes.tw. OR exp "Diabetes Mellitus, Type 2"/ OR exp "Insulin Resistance"/) AND (exp Exercise/ OR exp "Exercise Therapy"/ OR exp "Exercise Tolerance"/ OR "aerobic training".tw. OR "Resistance training".tw. OR exp "Caloric Restriction"/ OR "Very low energy diet".tw. OR exp "Diet, Reducing"/ OR "Viscous fibre".tw. OR exp "Dietary Fiber"/ OR exp "Dietary Supplements"/))  OR  ((exp Depression/ OR exp "Depressive Disorder"/ OR exp Anxiety/ OR exp "Anxiety Disorders"/) AND (exp Bibliotherapy/ OR books.tw. OR exp Exercise/ OR "aerobic training".tw. OR "Resistance training".tw. OR "group exercise".tw. OR exp "Cognitive Behavioral Therapy"/ OR "Online Cognitive Behavioral therapy".tw. OR CBT.tw. OR iCBT.tw. OR exp "Therapy, Computer-Assisted"/ OR exp Pschotherapy/))  OR  ((exp "Sleep initiation and Maintenance Disorders"/ OR Insomnia.tw. OR "Chronic Insomnia".tw. OR exp Sleep/) AND (exp Music/ OR exp "Music Therapy"/ OR exp Ascultation/ OR "Brief behavioral therapy".tw. OR "aerobic training".tw. OR exp "Cognitive Behavioral Therapy"/ OR CBT.tw. OR "sleep restriction".tw. OR exp "Behavioral Therapy"/ OR exp "Behavior Therapy"/))  OR  ((exp "Depression, Postpartum"/ OR "Postnatal depression".tw. OR PND.tw.) AND (exp Counseling/ OR exp Psychotherapy/ OR exp "Psychotherapy, Psychodynamic"/ OR exp "Interpersonal Psychotherapy"/ OR CBT.tw. OR IPT.tw.))  OR  ((exp "Panic Disorder"/ OR exp agoraphobia/) AND (exp "Cognitive Behavioral Therapy"/ OR CBT.tw. OR exp Psychotherapy/))  OR  ((exp "Back Pain"/ OR exp "Chronic Pain"/ OR exp "acute pain"/ OR exp "Low back Pain"/) AND (exp "Cognitive Behavioral Therapy"/ OR CBT.tw. OR exp Mindfulness/ OR exp "Mind-Body Therapies"/ OR exp Psychotherapy/ OR exp "Stress, Psychological"/ OR exp Yoga/))  OR  ((exp "Stress Disorders, Post Traumatic"/ OR PTSD.tw.) AND (exp "Eye Movement Desensitization Reprocessing"/ OR exp "cognitive behavioral therapy"/ OR exp "Implosive Therapy"/ OR "Trauma-focused psychological therapy".tw. OR "Dual attention psychological therapy".tw. OR EMDR.tw.))  OR  ((exp "Osteoarthritis, Hip"/ OR exp "Osteoarthritis, Knee"/ OR "Osteoarthritis, Hand".tw. OR (osteoarthritis AND pain).tw.) AND (exp "Aquatic Therapy"/ OR "Aquatic exercise".tw. OR exp Balneology/ OR exp Hydrotherapy/ OR exp Swimming/ OR exp Splints/ OR exp braces/ OR "joint protection strategies".tw. OR taping.tw. OR exp canes/ OR "walking stick".tw. OR exp "exercise therapy"/ OR exercises.tw. OR "strength exercise".tw. OR "aerobic exercise".tw. OR exp "Range of Motion"/ OR exp "Resistance Training"/ OR exp Walking/ OR exp adaptation/ OR exp "Combined modality therapy"/))  OR  (("Perineal massage".tw. OR exp Massage/) AND (antenatal.tw. OR prenatal.tw. OR exp Perineum/) AND (exp "delivery, obstetric"/ OR birth.tw. OR exp episiotomy/ OR exp "Obstetric Labor Complications"/))  OR  ((exp "Exercise therapy"/ OR exercises.tw. OR "muscle strengthening".tw. OR exp braces/) AND (exp "Ankle injuries"/ OR "sprain, ankle".tw.) AND (recurrent.tw. OR "recurrent injury".tw. OR "recurrent sprain".tw.))  OR  ((exp "Accidental Falls"/ OR "falls prevention".tw. OR exp "Accident Prevention"/ OR exp "Accidents, Home"/) AND (exp exercise/ OR exp "Exercise Therapy"/ OR exp "Resistance Training"/))  OR  ((exp "Patellofemoral Pain Syndrome"/ OR PFP.tw.) AND (exp exercise/ OR exp "Exercise Therapy"/ OR exp "Muscle Strength"/))  OR  ((exp Vertigo/ OR exp "Benign Paroxysmal Positional Vertigo"/) AND ("Epley manoeuvre".tw. OR exp "patient positioning"/))  OR  ((exp "Gastroesophageal Reflux"/ OR GERD.tw.) AND ("bed head elevation".tw. OR "Elevation of the bed head".tw.))  OR  ((exp "FODMAP Diet"/ OR "Low FODMAP".tw. OR exp "Diet therapy"/ OR exp "Diet, Carbohydrate-restricted"/) AND (exp "Irritable Bowel Syndrome"/ OR exp "gastrointestinal tract"/))  OR  ((Salt.tw. OR exp "Sodium Chloride"/ OR exp potassium/ OR salt-substitute.tw. OR "table salt".tw. OR exp "Diet, Sodium-Restricted"/) AND (exp "Cardiovascular Diseases"/ OR exp Hyperkalemia/ OR exp Hypokalemia/ OR exp Hypertension/ OR exp Stroke/ OR exp "Blood Pressure"/))  AND  (Cost*.tw. ADJ3 (Benefit* OR Effectiveness OR Utilit* OR saving* OR minimization OR analys* OR evaluat*).tw. OR (Economic.tw. ADJ3 (Evaluat* OR analys*).tw.) OR (Marginal ADJ3 Analys*).tw. OR (ICER OR "Incremental cost effectiveness ratio" OR "incremental cost-effectiveness ratios").tw. OR exp "Costs and Cost Analysis"/)  AND  (("Randomized controlled trial" OR "controlled clinical trial").pt. OR randomized.tw. OR randomised.tw. OR placebo.tw. OR "Drug Therapy".fs. OR randomly.tw. OR trial.tw. OR trials.ti. OR groups.tw.) NOT (exp Animals/ NOT (exp Animals/ AND exp Humans/)) NOT (("Case Reports" OR Editorial OR Letter OR Meta-Analysis OR "Observational Study" OR "Systematic Review").pt. OR "Case Report".ti. OR "Case series".ti. OR Meta-Analysis.ti. OR "Meta Analysis".ti. OR "Systematic Review".ti.) |
| --- |
| **CINAHL (Ebsco)**  ((TI "Non-Drug Intervention" OR AB "Non-Drug Intervention"") OR (TI "Non-drug interventions" OR AB "Non-drug interventions") OR (TI NDI OR AB NDI) OR (TI "Non-Drug Therap*" OR AB "Non-Drug Therap*") OR (TI "Non-pharmacological treatment" OR AB "Non-pharmacological treatment") OR (TI "non-pharmacological therapy" OR AB "non-pharmacological therapy"))  OR  ((TI "Mothers Kiss" OR AB "Mothers Kiss") AND ((MH "foreign body+") OR (TI "Nasal foreign body" OR AB "Nasal foreign body") OR (TI "foreign body, nose" OR AB "foreign body, nose") OR (MH "Nasal cavity+")))  OR  (((TI Autoinflation OR AB Autoinflation) OR (MH insufflation+) OR (MH "Valsalva Maneuver+")) AND ((TI "glue ear" OR AB "glue ear") OR (TI grommets OR AB grommets) OR (MH "Otitis Media with Effusion+") OR (MH "Acoustic Impedance Tests+") OR (MH "Eustachian Tube+") OR (MH "Hearing Loss+")))  OR  (((TI "Apple juice" OR AB "Apple juice") OR (MH "Fruit and Vegetable Juices+")) AND ((TI rehydration OR AB rehydration) OR (MH "rehydration Solutions+") OR (MH dehydration+) OR (MH electrolytes+) OR (MH "Fluid Therapy+") OR (MH Gastroenteritis+)))  OR  (((TI "pulled elbow" OR AB "pulled elbow") OR (MH "elbow injuries+") OR (MH radius+)) AND ((MH "Manipulation, Orthopedic+") OR (TI sublaxation OR AB sublaxation) OR (TI "radial head" OR AB "radial head") OR (MH "Sprains and Strains+") OR (MH supination+)))  OR  (((MH "pain management+") OR (MH pain+) OR (MH "acute pain+") OR (MH psychotherapy+) OR (TI "combined modality theory" OR AB "combined modality theory")) AND ((MH immunization+) OR (TI Immunisation OR AB Immunisation) OR (MH Vaccination+) OR (MH injections+) OR (MH Needles+)) AND ((TI children OR AB children) OR (MH child+) OR (MH Infant+) OR (MH "infant care+")))  OR  (((MH "pain management+") OR (MH pain+) OR (MH "acute pain+") OR (MH psychotherapy+) OR (TI "combined modality theory" OR AB "combined modality theory")) AND ((MH immunization+) OR (TI Immunisation OR AB Immunisation) OR (MH Vaccination+) OR (MH injections+) OR (MH Needles+)) AND ((TI children OR AB children) OR (MH child+) OR (MH Infant+) OR (MH "infant care+")))  OR  (((TI alarms OR AB alarms) OR (MH "clinical alarms+") OR (MH "Cognitive Behavioral Therapy+")) AND ((TI bedwetting OR AB bedwetting) OR (MH "nocturnal enuresis+") OR (MH enuresis+)) AND ((MH child+) OR (TI Children OR AB Children)))  OR  (((TI "infant sleep" OR AB "infant sleep") OR (TI "sleep programs" OR AB "sleep programs") OR (MH "Behavior therapy+") OR (MH "infant care+")) AND ((TI "maternal mood" OR AB "maternal mood") OR (TI "maternal mental health" OR AB "maternal mental health") OR (MH "Depression, Postpartum+") OR (MH "Mother-Child Relations+") OR (MH Mothers+) OR (MH "Mental Health+")))  OR  (((TI "early exposure" OR AB "early exposure") OR (TI "early introduction" OR AB "early introduction")) AND ((TI "allergenic food*" OR AB "allergenic food*") OR (TI allergy OR AB allergy) OR (TI egg OR AB egg) OR (TI peanut OR AB peanut)) AND ((TI prevention OR AB prevention) OR (TI "allergy prevention" OR AB "allergy prevention")) AND ((MH child+) OR (MH infant+)))  OR  (((TI reflux OR AB reflux) OR (TI "infant reflux" OR AB "infant reflux") OR (MH "gastroesophageal reflux+")) AND ((TI "feed thickener" OR AB "feed thickener") OR (MH "Food Additives+") OR (MH "infant formula+")))  OR  (((MH eczema+) OR (TI "atopic eczema" OR AB "atopic eczema") OR (MH "Dermatitis, atopic+")) AND ((TI Moisturiser OR AB Moisturiser) OR (MH probiotics+) OR (MH lactobacillus+)) AND ((TI "high-risk infants" OR AB "high-risk infants") OR (MH infants+) OR (MH pregnancy+) OR (MH "prenatal care+")))  OR  (((TI "wet combing" OR AB "wet combing") OR (TI conditioner OR AB conditioner)) AND ((TI "head lice" OR AB "head lice") OR (MH pediculus+) OR (MH "Scalp Dermatoses+") OR (MH "lice infestations+") OR (MH Pediculus+)))  OR  (((MH honey+)) AND ((MH cough+) OR (MH "Respiratory Tract Infections+") OR (TI URTI OR AB URTI) OR (TI "upper respiratory tract infection" OR AB "upper respiratory tract infection") OR (MH "antitussive agents+")) AND ((MH child+) OR (TI children OR AB children)))  OR  (((MH exercise+) OR (MH "exercise therapy+") OR (TI "exercise rehabilitation" OR AB "exercise rehabilitation")) AND ((MH "Heart failure+") OR (TI "heart failure symptoms" OR AB "heart failure symptoms") OR (MH "cardiac rehabilitation+")))  OR  (((MH exercise+) OR (MH "exercise therapy+") OR (MH walking+)) AND ((MH "Peripheral Arterial Disease+") OR (MH "intermittent claudication+") OR (TI claudication OR AB claudication)))  OR  (((MH "Valsalva Maneuver+") OR (TI "modified valsalva" OR AB "modified valsalva")) AND ((MH "Tachycardia, Supraventricular+") OR (TI SVT OR AB SVT) OR (TI "sinus rhythm" OR AB "sinus rhythm")))  OR  (((TI "fitness training" OR AB "fitness training") OR (MH exercise+) OR (TI "aerobic exercise" OR AB "aerobic exercise") OR (MH "resistance training+") OR "combined aerobic" AND (TI "resistance training" OR AB "resistance training") OR (MH "activities of daily living+") OR (MH "exercise therapy+") OR (MH "Physical fitness+")) AND ((MH stroke+) OR (TI "cerebrovascular accident" OR AB "cerebrovascular accident") OR (TI CVA OR AB CVA) OR (MH "Stroke rehabilitation+")))  OR  (((MH "diet, Mediterranean+") OR (MH "diet, fat-restricted+")) AND ((MH "Cardiovascular diseases+") OR (TI "Coronary Disease" OR AB "Coronary Disease") OR (TI CVD OR AB CVD) OR (TI "Cardiovascular disease risk" OR AB "Cardiovascular disease risk") OR (MH "Myocardial infarction+")))  OR  (((MH "preoperative exercise+") OR (TI "respiratory pre-rehabilitation" OR AB "respiratory pre-rehabilitation") OR (TI "preoperative inspiratory muscle training" OR AB "preoperative inspiratory muscle training") OR (MH "Breathing exercises+") OR (MH "Preoperative care+")) AND ((MH Surgery+) OR (TI "before surgery" OR AB "before surgery") OR (TI "Surgical procedure" OR AB "Surgical procedure") OR (MH "Cardiac surgical procedure+") OR (MH "Elective Surgical Procedures+")) AND ((TI "Postoperative pneumonia" OR AB "Postoperative pneumonia") OR (MH "postoperative complications+") OR (MH "pulmonary atelectasis+")))  OR  (((MH Lung+) OR (TI pulmonary OR AB pulmonary)) AND ((MH Rehabilitation+) OR (TI rehab OR AB rehab) OR (MH "Exercise Tolerance+")) AND ((MH "Pulmonary Disease, Chronic Obstructive+") OR (TI COPD OR AB COPD)))  OR  (((MH "Sleep Apnea, Obstructive+") OR (TI "Obstructive sleep apnoea" OR AB "Obstructive sleep apnoea") OR (MH "Disorders of Excessive Somnolence+")) AND ((MH "Continuous Positive Airway Pressure+") OR (MH "Positive-Pressure Respiration+") OR (MH "Occlusal Splints+") OR (TI CPAP OR AB CPAP) OR (TI "Mandibular Splint" OR AB "Mandibular Splint") OR (MH "Mandibular Advancement+")))  OR  (((MH "Diabetes Mellitus+") OR (TI Diabetes OR AB Diabetes) OR (MH "Diabetes Mellitus, Type 2+") OR (MH "Insulin Resistance+")) AND ((MH Exercise+) OR (MH "Exercise Therapy+") OR (MH "Exercise Tolerance+") OR (TI "aerobic training" OR AB "aerobic training") OR (TI "Resistance training" OR AB "Resistance training") OR (MH "Caloric Restriction+") OR (TI "Very low energy diet" OR AB "Very low energy diet") OR (MH "Diet, Reducing+") OR (TI "Viscous fibre" OR AB "Viscous fibre") OR (MH "Dietary Fiber+") OR (MH "Dietary Supplements+")))  OR  (((MH Depression+) OR (MH "Depressive Disorder+") OR (MH Anxiety+) OR (MH "Anxiety Disorders+")) AND ((MH Bibliotherapy+) OR (TI books OR AB books) OR (MH Exercise+) OR (TI "aerobic training" OR AB "aerobic training") OR (TI "Resistance training" OR AB "Resistance training") OR (TI "group exercise" OR AB "group exercise") OR (MH "Cognitive Behavioral Therapy+") OR (TI "Online Cognitive Behavioral therapy" OR AB "Online Cognitive Behavioral therapy") OR (TI CBT OR AB CBT) OR (TI iCBT OR AB iCBT) OR (MH "Therapy, Computer-Assisted+") OR (MH Pschotherapy+)))  OR  (((MH "Sleep initiation and Maintenance Disorders+") OR (TI Insomnia OR AB Insomnia) OR (TI "Chronic Insomnia" OR AB "Chronic Insomnia") OR (MH Sleep+)) AND ((MH Music+) OR (MH "Music Therapy+") OR (MH Ascultation+) OR (TI "Brief behavioral therapy" OR AB "Brief behavioral therapy") OR (TI "aerobic training" OR AB "aerobic training") OR (MH "Cognitive Behavioral Therapy+") OR (TI CBT OR AB CBT) OR (TI "sleep restriction" OR AB "sleep restriction") OR (MH "Behavioral Therapy+") OR (MH "Behavior Therapy+")))  OR  (((MH "Depression, Postpartum+") OR (TI "Postnatal depression" OR AB "Postnatal depression") OR (TI PND OR AB PND)) AND ((MH Counseling+) OR (MH Psychotherapy+) OR (MH "Psychotherapy, Psychodynamic+") OR (MH "Interpersonal Psychotherapy+") OR (TI CBT OR AB CBT) OR (TI IPT OR AB IPT)))  OR  (((MH "Panic Disorder+") OR (MH agoraphobia+)) AND ((MH "Cognitive Behavioral Therapy+") OR (TI CBT OR AB CBT) OR (MH Psychotherapy+)))  OR  (((MH "Back Pain+") OR (MH "Chronic Pain+") OR (MH "acute pain+") OR (MH "Low back Pain+")) AND ((MH "Cognitive Behavioral Therapy+") OR (TI CBT OR AB CBT) OR (MH Mindfulness+) OR (MH "Mind-Body Therapies+") OR (MH Psychotherapy+) OR (MH "Stress, Psychological+") OR (MH Yoga+)))  OR  (((MH "Stress Disorders, Post Traumatic+") OR (TI PTSD OR AB PTSD)) AND ((MH "Eye Movement Desensitization Reprocessing+") OR (MH "cognitive behavioral therapy+") OR (MH "Implosive Therapy+") OR (TI "Trauma-focused psychological therapy" OR AB "Trauma-focused psychological therapy") OR (TI "Dual attention psychological therapy" OR AB "Dual attention psychological therapy") OR (TI EMDR OR AB EMDR)))  OR  (((MH "Osteoarthritis, Hip+") OR (MH "Osteoarthritis, Knee+") OR (TI "Osteoarthritis, Hand" OR AB "Osteoarthritis, Hand") OR ((TI osteoarthritis OR AB osteoarthritis) AND (TI pain OR AB pain))) AND ((MH "Aquatic Therapy+") OR (TI "Aquatic exercise" OR AB "Aquatic exercise") OR (MH Balneology+) OR (MH Hydrotherapy+) OR (MH Swimming+) OR (MH Splints+) OR (MH braces+) OR (TI "joint protection strategies" OR AB "joint protection strategies") OR (TI taping OR AB taping) OR (MH canes+) OR (TI "walking stick" OR AB "walking stick") OR (MH "exercise therapy+") OR (TI exercises OR AB exercises) OR (TI "strength exercise" OR AB "strength exercise") OR (TI "aerobic exercise" OR AB "aerobic exercise") OR (MH "Range of Motion+") OR (MH "Resistance Training+") OR (MH Walking+) OR (MH adaptation+) OR (MH "Combined modality therapy+")))  OR  (((TI "Perineal massage" OR AB "Perineal massage") OR (MH Massage+)) AND ((TI antenatal OR AB antenatal) OR (TI prenatal OR AB prenatal) OR (MH Perineum+)) AND ((MH "delivery, obstetric+") OR (TI birth OR AB birth) OR (MH episiotomy+) OR (MH "Obstetric Labor Complications+")))  OR  (((MH "Exercise therapy+") OR (TI exercises OR AB exercises) OR (TI "muscle strengthening" OR AB "muscle strengthening") OR (MH braces+)) AND ((MH "Ankle injuries+") OR (TI "sprain, ankle" OR AB "sprain, ankle")) AND ((TI recurrent OR AB recurrent) OR (TI "recurrent injury" OR AB "recurrent injury") OR (TI "recurrent sprain" OR AB "recurrent sprain")))  OR  (((MH "Accidental Falls+") OR (TI "falls prevention" OR AB "falls prevention") OR (MH "Accident Prevention+") OR (MH "Accidents, Home+")) AND ((MH exercise+) OR (MH "Exercise Therapy+") OR (MH "Resistance Training+")))  OR  (((MH "Patellofemoral Pain Syndrome+") OR (TI PFP OR AB PFP)) AND ((MH exercise+) OR (MH "Exercise Therapy+") OR (MH "Muscle Strength+")))  OR  (((MH Vertigo+) OR (MH "Benign Paroxysmal Positional Vertigo+")) AND ((TI "Epley manoeuvre" OR AB "Epley manoeuvre") OR (MH "patient positioning+")))  OR  (((MH "Gastroesophageal Reflux+") OR (TI GERD OR AB GERD)) AND ((TI "bed head elevation" OR AB "bed head elevation") OR (TI "Elevation of the bed head" OR AB "Elevation of the bed head")))  OR  (((MH "FODMAP Diet+") OR (TI "Low FODMAP" OR AB "Low FODMAP") OR (MH "Diet therapy+") OR (MH "Diet, Carbohydrate-restricted+")) AND ((MH "Irritable Bowel Syndrome+") OR (MH "gastrointestinal tract+")))  OR  (((TI Salt OR AB Salt) OR (MH "Sodium Chloride+") OR (MH potassium+) OR (TI salt-substitute OR AB salt-substitute) OR (TI "table salt" OR AB "table salt") OR (MH "Diet, Sodium-Restricted+")) AND ((MH "Cardiovascular Diseases+") OR (MH Hyperkalemia+) OR (MH Hypokalemia+) OR (MH Hypertension+) OR (MH Stroke+) OR (MH "Blood Pressure+")))  AND  ((TI Cost* OR AB Cost*) N3 ((TI Benefit* OR AB Benefit*) OR (TI Effectiveness OR AB Effectiveness) OR (TI Utilit* OR AB Utilit*) OR (TI saving* OR AB saving*) OR (TI minimization OR AB minimization) OR (TI analys* OR AB analys*) OR (TI evaluat* OR AB evaluat*)) OR ((TI Economic OR AB Economic) N3 ((TI Evaluat* OR AB Evaluat*) OR (TI analys* OR AB analys*))) OR ((TI Marginal OR AB Marginal) N3 (TI Analys* OR AB Analys*)) OR ((TI ICER OR AB ICER) OR (TI "Incremental cost effectiveness ratio" OR AB "Incremental cost effectiveness ratio") OR (TI "incremental cost-effectiveness ratios" OR AB "incremental cost-effectiveness ratios")) OR (MH "Costs and Cost Analysis+"))  AND  (((PT "Randomized controlled trial") OR (PT "controlled clinical trial")) OR (TI randomized OR AB randomized) OR (TI randomised OR AB randomised) OR (TI placebo OR AB placebo) OR "Drug Therapy" OR (TI randomly OR AB randomly) OR (TI trial OR AB trial) OR (TI trials) OR (TI groups OR AB groups)) NOT ((MH Animals+) NOT ((MH Animals+) AND (MH Humans+))) NOT (((PT "Case Reports") OR (PT Editorial) OR (PT Letter) OR (PT Meta-Analysis) OR (PT "Observational Study") OR (PT "Systematic Review")) OR (TI "Case Report") OR (TI "Case series") OR (TI Meta-Analysis) OR (TI "Meta Analysis") OR (TI "Systematic Review")) |
| **PsychInfo (Ovid)**  ("Non-Drug Intervention".ti,ab. OR "Non-drug interventions".ti,ab. OR NDI.ti,ab. OR "Non-Drug Therap*".ti,ab. OR "Non-pharmacological treatment".ti,ab. OR "non-pharmacological therapy".ti,ab.)  OR  ("Mothers Kiss".ti,ab. AND (exp "foreign body"/ OR "Nasal foreign body".ti,ab. OR "foreign body, nose".ti,ab. OR exp "Nasal cavity"/))  OR  ((Autoinflation.ti,ab. OR exp insufflation/ OR exp "Valsalva Maneuver"/) AND ("glue ear".ti,ab. OR grommets.ti,ab. OR exp "Otitis Media with Effusion"/ OR exp "Acoustic Impedance Tests"/ OR exp "Eustachian Tube"/ OR exp "Hearing Loss"/))  OR  (("Apple juice".ti,ab. OR exp "Fruit and Vegetable Juices"/) AND (rehydration.ti,ab. OR exp "rehydration Solutions"/ OR exp dehydration/ OR exp electrolytes/ OR exp "Fluid Therapy"/ OR exp Gastroenteritis/))  OR  (("pulled elbow".ti,ab. OR exp "elbow injuries"/ OR exp radius/) AND (exp "Manipulation, Orthopedic"/ OR sublaxation.ti,ab. OR "radial head".ti,ab. OR exp "Sprains and Strains"/ OR exp supination/))  OR  ((exp "pain management"/ OR exp pain/ OR exp "acute pain"/ OR exp psychotherapy/ OR "combined modality theory".ti,ab.) AND (exp immunization/ OR Immunisation.ti,ab. OR exp Vaccination/ OR exp injections/ OR exp Needles/) AND (children.ti,ab. OR exp child/ OR exp Infant/ OR exp "infant care"/))  OR  ((exp "pain management"/ OR exp pain/ OR exp "acute pain"/ OR exp psychotherapy/ OR "combined modality theory".ti,ab.) AND (exp immunization/ OR Immunisation.ti,ab. OR exp Vaccination/ OR exp injections/ OR exp Needles/) AND (children.ti,ab. OR exp child/ OR exp Infant/ OR exp "infant care"/))  OR  ((alarms.ti,ab. OR exp "clinical alarms"/ OR exp "Cognitive Behavioral Therapy"/) AND (bedwetting.ti,ab. OR exp "nocturnal enuresis"/ OR exp enuresis/) AND (exp child/ OR Children.ti,ab.))  OR  (("infant sleep".ti,ab. OR "sleep programs".ti,ab. OR exp "Behavior therapy"/ OR exp "infant care"/) AND ("maternal mood".ti,ab. OR "maternal mental health".ti,ab. OR exp "Depression, Postpartum"/ OR exp "Mother-Child Relations"/ OR exp Mothers/ OR exp "Mental Health"/))  OR  (("early exposure".ti,ab. OR "early introduction".ti,ab.) AND ("allergenic food*".ti,ab. OR allergy.ti,ab. OR egg.ti,ab. OR peanut.ti,ab.) AND (prevention.ti,ab. OR "allergy prevention".ti,ab.) AND (exp child/ OR exp infant/))  OR  ((reflux.ti,ab. OR "infant reflux".ti,ab. OR exp "gastroesophageal reflux"/) AND ("feed thickener".ti,ab. OR exp "Food Additives"/ OR exp "infant formula"/))  OR  ((exp eczema/ OR "atopic eczema".ti,ab. OR exp "Dermatitis, atopic"/) AND (Moisturiser.ti,ab. OR exp probiotics/ OR exp lactobacillus/) AND ("high-risk infants".ti,ab. OR exp infants/ OR exp pregnancy/ OR exp "prenatal care"/))  OR  (("wet combing".ti,ab. OR conditioner.ti,ab.) AND ("head lice".ti,ab. OR exp pediculus/ OR exp "Scalp Dermatoses"/ OR exp "lice infestations"/ OR exp Pediculus/))  OR  ((exp honey/) AND (exp cough/ OR exp "Respiratory Tract Infections"/ OR URTI.ti,ab. OR "upper respiratory tract infection".ti,ab. OR exp "antitussive agents"/) AND (exp child/ OR children.ti,ab.))  OR  ((exp exercise/ OR exp "exercise therapy"/ OR "exercise rehabilitation".ti,ab.) AND (exp "Heart failure"/ OR "heart failure symptoms".ti,ab. OR exp "cardiac rehabilitation"/))  OR  ((exp exercise/ OR exp "exercise therapy"/ OR exp walking/) AND (exp "Peripheral Arterial Disease"/ OR exp "intermittent claudication"/ OR claudication.ti,ab.))  OR  ((exp "Valsalva Maneuver"/ OR "modified valsalva".ti,ab.) AND (exp "Tachycardia, Supraventricular"/ OR SVT.ti,ab. OR "sinus rhythm".ti,ab.))  OR  (("fitness training".ti,ab. OR exp exercise/ OR "aerobic exercise".ti,ab. OR exp "resistance training"/ OR "combined aerobic" AND "resistance training".ti,ab. OR exp "activities of daily living"/ OR exp "exercise therapy"/ OR exp "Physical fitness"/) AND (exp stroke/ OR "cerebrovascular accident".ti,ab. OR CVA.ti,ab. OR exp "Stroke rehabilitation"/))  OR  ((exp "diet, Mediterranean"/ OR exp "diet, fat-restricted"/) AND (exp "Cardiovascular diseases"/ OR "Coronary Disease".ti,ab. OR CVD.ti,ab. OR "Cardiovascular disease risk".ti,ab. OR exp "Myocardial infarction"/))  OR  ((exp "preoperative exercise"/ OR "respiratory pre-rehabilitation".ti,ab. OR "preoperative inspiratory muscle training".ti,ab. OR exp "Breathing exercises"/ OR exp "Preoperative care"/) AND (exp Surgery/ OR "before surgery".ti,ab. OR "Surgical procedure".ti,ab. OR exp "Cardiac surgical procedure"/ OR exp "Elective Surgical Procedures"/) AND ("Postoperative pneumonia".ti,ab. OR exp "postoperative complications"/ OR exp "pulmonary atelectasis"/))  OR  ((exp Lung/ OR pulmonary.ti,ab.) AND (exp Rehabilitation/ OR rehab.ti,ab. OR exp "Exercise Tolerance"/) AND (exp "Pulmonary Disease, Chronic Obstructive"/ OR COPD.ti,ab.))  OR  ((exp "Sleep Apnea, Obstructive"/ OR "Obstructive sleep apnoea".ti,ab. OR exp "Disorders of Excessive Somnolence"/) AND (exp "Continuous Positive Airway Pressure"/ OR exp "Positive-Pressure Respiration"/ OR exp "Occlusal Splints"/ OR CPAP.ti,ab. OR "Mandibular Splint".ti,ab. OR exp "Mandibular Advancement"/))  OR  ((exp "Diabetes Mellitus"/ OR Diabetes.ti,ab. OR exp "Diabetes Mellitus, Type 2"/ OR exp "Insulin Resistance"/) AND (exp Exercise/ OR exp "Exercise Therapy"/ OR exp "Exercise Tolerance"/ OR "aerobic training".ti,ab. OR "Resistance training".ti,ab. OR exp "Caloric Restriction"/ OR "Very low energy diet".ti,ab. OR exp "Diet, Reducing"/ OR "Viscous fibre".ti,ab. OR exp "Dietary Fiber"/ OR exp "Dietary Supplements"/))  OR  ((exp Depression/ OR exp "Depressive Disorder"/ OR exp Anxiety/ OR exp "Anxiety Disorders"/) AND (exp Bibliotherapy/ OR books.ti,ab. OR exp Exercise/ OR "aerobic training".ti,ab. OR "Resistance training".ti,ab. OR "group exercise".ti,ab. OR exp "Cognitive Behavioral Therapy"/ OR "Online Cognitive Behavioral therapy".ti,ab. OR CBT.ti,ab. OR iCBT.ti,ab. OR exp "Therapy, Computer-Assisted"/ OR exp Pschotherapy/))  OR  ((exp "Sleep initiation and Maintenance Disorders"/ OR Insomnia.ti,ab. OR "Chronic Insomnia".ti,ab. OR exp Sleep/) AND (exp Music/ OR exp "Music Therapy"/ OR exp Ascultation/ OR "Brief behavioral therapy".ti,ab. OR "aerobic training".ti,ab. OR exp "Cognitive Behavioral Therapy"/ OR CBT.ti,ab. OR "sleep restriction".ti,ab. OR exp "Behavioral Therapy"/ OR exp "Behavior Therapy"/))  OR  ((exp "Depression, Postpartum"/ OR "Postnatal depression".ti,ab. OR PND.ti,ab.) AND (exp Counseling/ OR exp Psychotherapy/ OR exp "Psychotherapy, Psychodynamic"/ OR exp "Interpersonal Psychotherapy"/ OR CBT.ti,ab. OR IPT.ti,ab.))  OR  ((exp "Panic Disorder"/ OR exp agoraphobia/) AND (exp "Cognitive Behavioral Therapy"/ OR CBT.ti,ab. OR exp Psychotherapy/))  OR  ((exp "Back Pain"/ OR exp "Chronic Pain"/ OR exp "acute pain"/ OR exp "Low back Pain"/) AND (exp "Cognitive Behavioral Therapy"/ OR CBT.ti,ab. OR exp Mindfulness/ OR exp "Mind-Body Therapies"/ OR exp Psychotherapy/ OR exp "Stress, Psychological"/ OR exp Yoga/))  OR  ((exp "Stress Disorders, Post Traumatic"/ OR PTSD.ti,ab.) AND (exp "Eye Movement Desensitization Reprocessing"/ OR exp "cognitive behavioral therapy"/ OR exp "Implosive Therapy"/ OR "Trauma-focused psychological therapy".ti,ab. OR "Dual attention psychological therapy".ti,ab. OR EMDR.ti,ab.))  OR  ((exp "Osteoarthritis, Hip"/ OR exp "Osteoarthritis, Knee"/ OR "Osteoarthritis, Hand".ti,ab. OR (osteoarthritis.ti,ab. AND pain.ti,ab.)) AND (exp "Aquatic Therapy"/ OR "Aquatic exercise".ti,ab. OR exp Balneology/ OR exp Hydrotherapy/ OR exp Swimming/ OR exp Splints/ OR exp braces/ OR "joint protection strategies".ti,ab. OR taping.ti,ab. OR exp canes/ OR "walking stick".ti,ab. OR exp "exercise therapy"/ OR exercises.ti,ab. OR "strength exercise".ti,ab. OR "aerobic exercise".ti,ab. OR exp "Range of Motion"/ OR exp "Resistance Training"/ OR exp Walking/ OR exp adaptation/ OR exp "Combined modality therapy"/))  OR  (("Perineal massage".ti,ab. OR exp Massage/) AND (antenatal.ti,ab. OR prenatal.ti,ab. OR exp Perineum/) AND (exp "delivery, obstetric"/ OR birth.ti,ab. OR exp episiotomy/ OR exp "Obstetric Labor Complications"/))  OR  ((exp "Exercise therapy"/ OR exercises.ti,ab. OR "muscle strengthening".ti,ab. OR exp braces/) AND (exp "Ankle injuries"/ OR "sprain, ankle".ti,ab.) AND (recurrent.ti,ab. OR "recurrent injury".ti,ab. OR "recurrent sprain".ti,ab.))  OR  ((exp "Accidental Falls"/ OR "falls prevention".ti,ab. OR exp "Accident Prevention"/ OR exp "Accidents, Home"/) AND (exp exercise/ OR exp "Exercise Therapy"/ OR exp "Resistance Training"/))  OR  ((exp "Patellofemoral Pain Syndrome"/ OR PFP.ti,ab.) AND (exp exercise/ OR exp "Exercise Therapy"/ OR exp "Muscle Strength"/))  OR  ((exp Vertigo/ OR exp "Benign Paroxysmal Positional Vertigo"/) AND ("Epley manoeuvre".ti,ab. OR exp "patient positioning"/))  OR  ((exp "Gastroesophageal Reflux"/ OR GERD.ti,ab.) AND ("bed head elevation".ti,ab. OR "Elevation of the bed head".ti,ab.))  OR  ((exp "FODMAP Diet"/ OR "Low FODMAP".ti,ab. OR exp "Diet therapy"/ OR exp "Diet, Carbohydrate-restricted"/) AND (exp "Irritable Bowel Syndrome"/ OR exp "gastrointestinal tract"/))  OR  ((Salt.ti,ab. OR exp "Sodium Chloride"/ OR exp potassium/ OR salt-substitute.ti,ab. OR "table salt".ti,ab. OR exp "Diet, Sodium-Restricted"/) AND (exp "Cardiovascular Diseases"/ OR exp Hyperkalemia/ OR exp Hypokalemia/ OR exp Hypertension/ OR exp Stroke/ OR exp "Blood Pressure"/))  AND  (Cost*.ti,ab. ADJ3 (Benefit*.ti,ab. OR Effectiveness.ti,ab. OR Utilit*.ti,ab. OR saving*.ti,ab. OR minimization.ti,ab. OR analys*.ti,ab. OR evaluat*.ti,ab.) OR (Economic.ti,ab. ADJ3 (Evaluat*.ti,ab. OR analys*.ti,ab.)) OR (Marginal.ti,ab. ADJ3 Analys*.ti,ab.) OR (ICER.ti,ab. OR "Incremental cost effectiveness ratio".ti,ab. OR "incremental cost-effectiveness ratios".ti,ab.) OR exp "Costs and Cost Analysis"/)  AND  (("Randomized controlled trial".pt. OR "controlled clinical trial".pt.) OR randomized.ti,ab. OR randomised.ti,ab. OR placebo.ti,ab. OR "Drug Therapy" OR randomly.ti,ab. OR trial.ti,ab. OR trials.ti. OR groups.ti,ab.) NOT (exp Animals/ NOT (exp Animals/ AND exp Humans/)) NOT (("Case Reports".pt. OR Editorial.pt. OR Letter.pt. OR Meta-Analysis.pt. OR "Observational Study".pt. OR "Systematic Review".pt.) OR "Case Report".ti. OR "Case series".ti. OR Meta-Analysis.ti. OR "Meta Analysis".ti. OR "Systematic Review".ti.) |

**Supplementary Table 2** Inclusion and exclusion criteria

|  | **Include** | **Exclude** |
| --- | --- | --- |
| **Participants** | Individuals of any age, sex, or ethnicity | Individuals in institutions (e.g., acute care, hospitals, prisons), and those receiving ongoing, high-level outpatient treatment |
| **Interventions** | Cost effectiveness evaluations of any Non-Drug Intervention(s) (NDIs) in the RACGP Handbook of Non-Drug Interventions (HANDI) prescribed to patients | Complex treatments or interventions where the NDI is used alongside another treatment and the effect of the NDI cannot be distinguished.  Use of the NDI without consultation with a primary health practitioner. |
| **Control/Comparator** | Usual care, no, care or use of pharmaceutical intervention |  |
| **Outcome** | Incremental Cost Effectiveness Ratios (ICERs) and any economic evaluation (cost-benefit, cost-utility, cost-effectiveness analyses) | Economic Models based on non-trial data. |
| **Setting** | Primary care settings, including telehealth and internet-based consultations (e.g., General Practice, community-based allied health clinics, community health clinics) | Institutionalized health settings (e.g., acute care, hospitals) |
| **Study Design** | Randomized Control Trials | Observational studies, Cohort Studies, Case-Control studies, Cross-sectional studies, Surveys, Systematic reviews, Scoping reviews, |

**Supplementary Table 3** List of excluded studies from original search with reasons for exclusion

(n =93)

|  | **Reference** | **Reason to exclude** |  |
| --- | --- | --- | --- |
| 1. | Abdelaziz EM, Elsharkawy NB, Mohamed SM. Efficacy of Internet-based cognitive behavioral therapy on sleeping difficulties in menopausal women: A randomized controlled trial. Perspect Psychiatr Care. 2022;58(4):1907-17. | No economic analysis | |
| 2. | Aboagye E, Karlsson ML, Hagberg J, Jensen I. Cost-effectiveness of early interventions for non-specific low back pain: a randomized controlled study investigating medical yoga, exercise therapy and self-care advice. J Rehabil Med. 2015;47(2):167-73. | Intervention not in HANDI | |
| 3. | Acarturk C, Konuk E, Cetinkaya M, Senay I, Sijbrandij M, Cuijpers P, et al. EMDR for Syrian refugees with posttraumatic stress disorder symptoms: Results of a pilot randomized controlled trial. European Journal of Psychotraumatology Vol 6 2015, ArtID 27414. 2015;6. | No economic analysis | |
| 4. | Alhambra-Borras T, Dura-Ferrandis E, Ferrando-Garcia M. Effectiveness and Estimation of Cost-Effectiveness of a Group-Based Multicomponent Physical Exercise Programme on Risk of Falling and Frailty in Community-Dwelling Older Adults. International Journal of Environmental Research & Public Health [Electronic Resource]. 2019;16(12). | Non trial data analysis | |
| 5. | An J, Glick HA, Sawyer AM, Arguelles J, Bae CJ, Keenan BT, et al. Association Between Positive Airway Pressure Adherence and Health Care Costs Among Individuals With OSA. Chest. 2023;163(6). | Wrong intervention type | |
| 6. | Baldassarre ME, Di Mauro A, Pignatelli MC, Fanelli M, Salvatore S, Di Nardo G, et al. Magnesium Alginate in Gastro-Esophageal Reflux: A Randomized Multicenter Cross-Over Study in Infants. International Journal of Environmental Research & Public Health [Electronic Resource]. 2019;17(1). | No distinguishable comparison group as both part of HANDI | |
| 7. | Bello AI, Quartey J, Lartey M. Efficacy of Behavioural Graded Activity Compared with Conventional Exercise Therapy in Chronic Non-Specific Low Back Pain: Implication for Direct Health Care Cost. Ghana Medical Journal. 2015;49(3). | No distinguishable comparison group as both part of HANDI | |
| 8. | Benz F, Grolig L, Hannibal S, Buntrock C, Cuijpers P, Domschke K, et al. Investigating non-inferiority of internet-delivered versus face-to-face cognitive behavioural therapy for insomnia (CBT-I): a randomised controlled trial (iSleep well). Trials. 2024;25(1):371. | Wrong study type-“protocol” | |
| 9. | Bergstrom J. Internet-based treatment for depression and panic disorder: From development to deployment. Dissertation Abstracts International: Section B: The Sciences and Engineering. 2022;83(2-B). | Wrong study type | |
| 10. | Bohingamu Mudiyanselage S, Stevens J, Watts JJ, Toscano J, Kotowicz MA, Steinfort CL, et al. Personalised telehealth intervention for chronic disease management: A pilot randomised controlled trial. Journal of Telemedicine & Telecare. 2019;25(6). | Intervention not in HANDI | |
| 11. | Botella C, Garcia-Palacios A. The possibility of reducing therapist contact and total length of therapy in the treatment of panic disorder. Behavioural and Cognitive Psychotherapy. 1999;27(3). | No economic analysis | |
| 12. | Bothelius K, Kyhle K, Espie CA, Broman JE. Manual-guided cognitive-behavioural therapy for insomnia delivered by ordinary primary care personnel in general medical practice: a randomized controlled effectiveness trial. Journal of Sleep Research. 2013;22(6). | No economic analysis | |
| 13. | Brun JF, Bordenave S, Mercier J, Jaussent A, Picot MC, Prefaut C. Cost-sparing effect of twice-weekly targeted endurance training in type 2 diabetics: a one-year controlled randomized trial. Diabetes & Metabolism. 2008;34(3). | No economic analysis | |
| 14. | Buchner DM, Cress ME, de Lateur BJ, Esselman PC, Margherita AJ, Price R, et al. The effect of strength and endurance training on gait, balance, fall risk, and health services use in community-living older adults. Journals of Gerontology Series A-Biological Sciences & Medical Sciences. 1997;52(4). | No economic analysis | |
| 15. | Bulthuis Y, Mohammad S, Braakman-Jansen LM, Drossaers-Bakker KW, van de Laar MA. Cost-effectiveness of intensive exercise therapy directly following hospital discharge in patients with arthritis: results of a randomized controlled clinical trial. Arthritis & Rheumatism. 2008;59(2). | Condition not part of HANDI | |
| 16. | Byrne MK. Pharmacotherapy and cognitive behavioural therapy: similarly cost effective compared with community referral for disadvantaged women with major depression. Evidence-based Mental Health. 2006;9(1). | Wrong study type | |
| 17. | Callaghan MJ, Oldham J, Hunt J. An evaluation of exercise regimes for patients with osteoarthritis of the knee: a single-blind randomized controlled trial. Clinical Rehabilitation. 1995;9(3). | No economic analysis | |
| 18. | Campbell AJ, Robertson MC, La Grow SJ, Kerse NM, Sanderson GF, Jacobs RJ, et al. Randomised controlled trial of prevention of falls in people aged > or =75 with severe visual impairment: the VIP trial. BMJ. 2005;331(7520). | Intervention not in HANDI | |
| 19. | Canaway A, Pincus T, Underwood M, Shapiro Y, Chodick G, Ben-Ami N. Is an enhanced behaviour change intervention cost-effective compared with physiotherapy for patients with chronic low back pain? Results from a multicentre trial in Israel. BMJ Open. 2018;8(4). | Intervention not in HANDI | |
| 20. | Castro WP, Sanchez MJR, Gonzalez CTP, Bethencourt JM, de la Fuente Portero JA, Marco RG. Cognitive-behavioral treatment and antidepressants combined with virtual reality exposure for patients with chronic agoraphobia. International Journal of Clinical and Health Psychology. 2014;14(1). | No economic analysis | |
| 21. | Chai-Coetzer CL, Antic NA, Rowland LS, Reed RL, Esterman A, Catcheside PG, et al. Primary care vs specialist sleep center management of obstructive sleep apnea and daytime sleepiness and quality of life: a randomized trial. JAMA. 2013;309(10). | No economic analysis | |
| 22. | Chan KL, Pau NHY, Poon SFO, Lam BLH, Lam CB, Chan KKS. Effectiveness of internet-based self-help cognitive behavioral therapy for insomnia (CBT-I) among community adults: A randomized controlled trial. Applied psychology Health and well-being. 2025;17(3):e70047. | No economic analysis | |
| 23. | Chang H, Hu Y, Yue CJ, Wen Y, Yeh W, Hsu L, et al. Effect of potassium-enriched salt on cardiovascular mortality and medical expenses of elderly men. American Journal of Clinical Nutrition. 2006;83(6). | No economic analysis | |
| 24. | Cohen LL. A comparative study of distraction and EMLA for pediatric pain management during immunizations. (distress, coping). Dissertation Abstracts International: Section B: The Sciences and Engineering. 1999;59(10-B). | Intervention not in HANDI | |
| 25. | Davies G, Jordan S, Brooks CJ, Thayer D, Storey M, Morgan G, et al. Long term extension of a randomised controlled trial of probiotics using electronic health records. Scientific Reports. 2018;8(1). | Non trial data analysis | |
| 26. | de Bont P, van der Vleugel BM, van den Berg DPG, de Roos C, Lokkerbol J, Smit F, et al. Health-economic benefits of treating trauma in psychosis. European Journal of Psychotraumatology. 2019;10(1). | Wrong study type | |
| 27. | Docking S, Ademi Z, Barton C, Wallis JA, Harris IA, de Steiger R, et al. Lifetime Cost-Effectiveness of Structured Education and Exercise Therapy for Knee Osteoarthritis in Australia. JAMA network open. 2024;7(10):e2436715. | Economic Models based on non-trial data. | |
| 28. | Dugas MJ, Ladouceur R, Leger E, Freeston MH, Langlois F, Provencher MD, et al. Group cognitive-behavioral therapy for generalized anxiety disorder: treatment outcome and long-term follow-up. J Consult Clin Psychol. 2003;71(4):821-5. | No economic analysis | |
| 29. | Egger N, Konnopka A, Beutel ME, Herpertz S, Hiller W, Hoyer J, et al. Long-term cost-effectiveness of cognitive behavioral therapy versus psychodynamic therapy in social anxiety disorder. Depression and Anxiety. 2016;33(12). | No economic analysis | |
| 30. | Farias CC, Resqueti V, Dias FA, Borghi-Silva A, Arena R, Fregonezi GA. Costs and benefits of pulmonary rehabilitation in chronic obstructive pulmonary disease: a randomized controlled trial. Brazilian Journal of Physical Therapy. 2014;18(2). | No economic analysis | |
| 31. | Frolova EV, Plavinskii SL, Moiseeva IE, Kuznetsova O, Filatov VN. [Efficacy of nondrug correction of hypertension in general medical practice]. Kardiologiia. 2004;44(2). | Wrong study type | |
| 32. | Goossens ME, Rutten-Van Molken MP, Kole-Snijders AM, Vlaeyen JW, Van Breukelen G, Leidl R. Health economic assessment of behavioural rehabilitation in chronic low back pain: a randomised clinical trial. Health Economics. 1998;7(1). | No economic analysis | |
| 33. | Guest JF, Panca M, Sladkevicius E, Taheri S, Stradling J. Clinical outcomes and cost-effectiveness of continuous positive airway pressure to manage obstructive sleep apnea in patients with type 2 diabetes in the U.K. Diabetes Care. 2014;37(5). | Wrong study type | |
| 34. | Harrington R, Taylor G, Hollinghurst S, Reed M, Kay H, Wood VA. A community-based exercise and education scheme for stroke survivors: a randomized controlled trial and economic evaluation. Clinical Rehabilitation. 2010;24(1). | No economic analysis | |
| 35. | Hedman E. Internet-based cognitive behaviour therapy for social anxiety disorder: From efficacy to effectiveness. Dissertation Abstracts International: Section B: The Sciences and Engineering. 2022;83(2-B). | Duplicate | |
| 36. | Hiscock H, Bayer J, Gold L, Hampton A, Ukoumunne OC, Wake M. Improving infant sleep and maternal mental health: a cluster randomised trial. Archives of Disease in Childhood. 2007;92(11). | No economic analysis | |
| 37. | Huckfeldt PJ, Frenier C, Pajewski NM, Espeland M, Peters A, Casanova R, et al. Associations of Intensive Lifestyle Intervention in Type 2 Diabetes With Health Care Use, Spending, and Disability: An Ancillary Study of the Look AHEAD Study. JAMA Network Open. 2020;3(11). | No economic analysis | |
| 38. | Huebschmann AG, Glasgow RE, Leavitt IM, Chapman K, Rice JD, Lockhart S, et al. Integrating a physical activity coaching intervention into diabetes care: a mixed-methods evaluation of a pilot pragmatic trial. Translational Behavioral Medicine. 2022;12(4). | No economic analysis | |
| 39. | Ikic V, Belanger C, Bouchard S, Gosselin P, Langlois F, Labrecque J, et al. Reduction in costs after treating comorbid panic disorder with agoraphobia and generalized anxiety disorder. Journal of Mental Health Policy and Economics. 2017;20(1). | No economic analysis | |
| 40. | Iliffe S, Kendrick D, Morris R, Masud T, Gage H, Skelton D, et al. Multicentre cluster randomised trial comparing a community group exercise programme and home-based exercise with usual care for people aged 65 years and over in primary care. Health Technology Assessment (Winchester, England). 2014;18(49). | No appropriate economic analysis | |
| 41. | Ismail K, Maissi E, Thomas S, Chalder T, Schmidt U, Bartlett J, et al. A randomised controlled trial of cognitive behaviour therapy and motivational interviewing for people with Type 1 diabetes mellitus with persistent sub-optimal glycaemic control: a Diabetes and Psychological Therapies (ADaPT) study. Health Technology Assessment (Winchester, England). 2010;14(22). | Intervention not in HANDI | |
| 42. | Jansen C-P, Nerz C, Labudek S, Gottschalk S, Kramer-Gmeiner F, Klenk J, et al. Lifestyle-integrated functional exercise to prevent falls and promote physical activity: Results from the LiFE-is-LiFE randomized non-inferiority trial. The International Journal of Behavioral Nutrition and Physical Activity Vol 18 2021, ArtID 115. 2021;18. | No economic analysis | |
| 43. | Joesch J, Sherbourne C, Sullivan G, Stein M, Craske M, Roy-Byrne P. Incremental benefits and cost of coordinated anxiety learning and management for anxiety treatment in primary care. Psychological Medicine. 2012;42(9). | Intervention not appropriate for HANDI | |
| 44. | Johannesson M, Aberg H, Agreus L, Borgquist L, Jonsson B. Cost-benefit analysis of non-pharmacological treatment of hypertension. Journal of Internal Medicine. 1991;230(4). | No economic analysis | |
| 45. | Juhakoski R, Tenhonen S, Malmivaara A, Kiviniemi V, Anttonen T, Arokoski JP. A pragmatic randomized controlled study of the effectiveness and cost consequences of exercise therapy in hip osteoarthritis. Clinical Rehabilitation. 2011;25(4). | Intervention not in HANDI | |
| 46. | Kaufman BG, Allen KD, Coffman CJ, Woolson S, Caves K, Hall K, et al. Cost and Quality of Life Outcomes of the STepped Exercise Program for Patients With Knee OsteoArthritis Trial. Value in Health. 2022;25(4). | Non trial data analysis | |
| 47. | Keane LG. Comparing AquaStretch with supervised land based stretching for Chronic Lower Back Pain. Journal of Bodywork & Movement Therapies. 2017;21(2). | No economic analysis | |
| 48. | Kemmler W, von Stengel S, Engelke K, Haberle L, Kalender WA. Exercise effects on bone mineral density, falls, coronary risk factors, and health care costs in older women: the randomized controlled senior fitness and prevention (SEFIP) study. Archives of Internal Medicine. 2010;170(2). | No economic analysis | |
| 49. | Kent P, Haines T, O'Sullivan P, Smith A, Campbell A, Schutze R, et al. Cognitive functional therapy with or without movement sensor biofeedback versus usual care for chronic, disabling low back pain (RESTORE): a randomised, controlled, three-arm, parallel group, phase 3, clinical trial. Lancet. 2023;401(10391). | Intervention not in HANDI | |
| 50. | Kettunen JA, Harilainen A, Sandelin J, Schlenzka D, Hietaniemi K, Seitsalo S, et al. Knee arthroscopy and exercise versus exercise only for chronic patellofemoral pain syndrome: a randomized controlled trial. BMC Medicine. 2007;5. | No economic analysis | |
| 51. | Kopp PT, Yang C, Yang H, Katz JN, Paltiel AD, Hunter DJ, et al. Cost-Effectiveness of Community-Based Diet and Exercise for Patients with Knee Osteoarthritis and Obesity or Overweight. Arthritis care & research. 2024;76(7):1018-27. | Economic Models based on non-trial data | |
| 52. | Kyle SD, Siriwardena AN, Espie CA, Yang Y, Petrou S, Ogburn E, et al. Clinical and cost-effectiveness of nurse-delivered sleep restriction therapy for insomnia in primary care (HABIT): a pragmatic, superiority, open-label, randomised controlled trial. Lancet (London, England). 2023;402(10406):975-87. | Duplicate | |
| 53. | Lai X, Yuan Y, Wang H, Zhang R, Qiao Q, Feng X, et al. Cost-Effectiveness of Salt Substitute and Salt Supply Restriction in Eldercare Facilities: The DECIDE-Salt Cluster Randomized Clinical Trial. JAMA network open. 2024;7(2):e2355564. | Duplicate | |
| 54. | Lamb SE, Bruce J, Hossain A, Chen J, Longo R, Lall R, et al. Screening and Intervention to Prevent Falls and Fractures in Older People. New England Journal of Medicine. 2020;383(19). | No economic analysis | |
| 55. | Lee HL, Mehta T, Ray B, Heng MS, McCollum PT, Chetter IC. A non-randomised controlled trial of the clinical and cost effectiveness of a Supervised Exercise Programme for claudication. European Journal of Vascular & Endovascular Surgery. 2007;33(2). | No economic analysis | |
| 56. | Lee Y-C, Gao L, Dear BF, Titov N, Mihalopoulos C. The cost-effectiveness of the online MindSpot Clinic for the treatment of depression and anxiety in Australia. Journal of Mental Health Policy and Economics. 2017;20(4). | Non trial data analysis | |
| 57. | Linton SJ, Nordin E. A 5-year follow-up evaluation of the Health and economic consequences of an early cognitive behavioral intervention for back pain: a randomized, controlled trial. Spine (03622436). 2006;31(8). | No economic analysis | |
| 58. | Littlewood E, Duarte A, Hewitt C, Knowles S, Palmer S, Walker S, et al. A randomised controlled trial of computerised cognitive behaviour therapy for the treatment of depression in primary care: the Randomised Evaluation of the Effectiveness and Acceptability of Computerised Therapy (REEACT) trial. Health Technology Assessment (Winchester, England). 2015;19(101). | Dupllicate | |
| 59. | Lojander J, Rasanen P, Sintonen H, Roine RP. Effect of nasal continuous positive airway pressure therapy on health-related quality of life in sleep apnoea patients treated in the routine clinical setting of a university hospital. Journal of International Medical Research. 2008;36(4). | Non- Primary care based intervention | |
| 60. | Macfarlane GJ, Beasley M, Scott N, Chong H, McNamee P, McBeth J, et al. Maintaining musculoskeletal health using a behavioural therapy approach: a population-based randomised controlled trial (the MAmMOTH Study). Annals of the Rheumatic Diseases. 2021;80(7). | Intervention not in HANDI | |
| 61. | Marchand A, Germain V, Reinharz D, Mainguy N, Landry P. [Analysis of the cost and the effectiveness of a psychotherapy for panic disorder with agoraphobia (PDA) versus a treatment combining pharmacotherapy and psychotherapy]. Sante Mentale au Quebec. 2004;29(2). | Non trial data analysis | |
| 62. | Mavranezouli I, Megnin-Viggars O, Grey N, Bhutani G, Leach J, Daly C, et al. Cost-effectiveness of psychological treatments for post-traumatic stress disorder in adults. PLoS ONE Vol 15(4), 2020, ArtID e0232245. 2020;15(4). | Non trial data analysis | |
| 63. | Mavranezouli I, Megnin-Viggars O, Trickey D, Meiser-Stedman R, Daly C, Dias S, et al. Cost-effectiveness of psychological interventions for children and young people with post-traumatic stress disorder. Journal of Child Psychology and Psychiatry. 2020;61(6). | Non trial data analysis | |
| 64. | McLean K, Day L, Dalton A. Economic evaluation of a group-based exercise program for falls prevention among the older community-dwelling population. BMC Geriatrics. 2015;15. | Non trial data analyis | |
| 65. | Mohr DC, Lattie EG, Tomasino KN, Kwasny MJ, Kaiser SM, Gray EL, et al. A randomized noninferiority trial evaluating remotely-delivered stepped care for depression using internet cognitive behavioral therapy (CBT) and telephone CBT. Behaviour Research & Therapy. 2019;123. | No economic analysis | |
| 66. | Monga S. Brief guided parent-delivered CBT is both efficacious and cost-effective for childhood anxiety disorders...Creswell C, Violato M, Fairbanks H, et al. Clinical outcomes and cost-effectiveness of brief guided parent-delivered cognitive behavioural therapy and solution-focused brief therapy for treatment of childhood anxiety disorders: a randomised controlled trial. Lancet Psychiatry 2017;4:529–39. Evidence-based Mental Health. 2018;21(2). | Wrong study type | |
| 67. | Moreno O, Meoro A, Martinez A, Rodriguez C, Pardo C, Aznar S, et al. Comparison of two low-calorie diets: a prospective study of effectiveness and safety. Journal of Endocrinological Investigation. 2006;29(7). | No economic analysis | |
| 68. | Moretti ME, Ungar WJ, Freedman SB, Schuh S. Cost-effectiveness of preferred fluids versus electrolytes in pediatric gastroenteritis. CJEM Canadian Journal of Emergency Medical Care. 2021;23(5). | Non trial data analysis | |
| 69. | Newman MG, Kenardy J, Herman S, Taylor C. Comparison of palmtop-computer-assisted brief cognitive-behavioral treatment to cognitive-behavioral treatment for panic disorder. Journal of Consulting and Clinical Psychology. 1997;65(1). | No economic analysis | |
| 70. | Norton G, McDonough CM, Cabral H, Shwartz M, Burgess JF. Cost-utility of cognitive behavioral therapy for low back pain from the commercial payer perspective. Spine. 2015;40(10). | Non trial data analysis | |
| 71. | Oostdam N, Bosmans J, Wouters MG, Eekhoff EM, van Mechelen W, van Poppel MN. Cost-effectiveness of an exercise program during pregnancy to prevent gestational diabetes: results of an economic evaluation alongside a randomised controlled trial. BMC Pregnancy & Childbirth. 2012;12. | Condition not in HANDI | |
| 72. | Paganini S, Lin J, Kahlke F, Buntrock C, Leiding D, Ebert DD, et al. A guided and unguided internet- and mobile-based intervention for chronic pain: health economic evaluation alongside a randomised controlled trial. BMJ Open. 2019;9(4). | Intervention not in HANDI | |
| 73. | Panagiotakos D, Sitara M, Pitsavos C, Stefanadis C. Estimating the 10-year risk of cardiovascular disease and its economic consequences, by the level of adherence to the Mediterranean diet: the ATTICA study. Journal of Medicinal Food. 2007;10(2). | Wrong study type/design | |
| 74. | Pritchard DA, Hyndman J, Taba F. Nutritional counselling in general practice: a cost effective analysis. Journal of Epidemiology & Community Health. 1999;53(5). | Intervention not in HANDI | |
| 75. | Rebelo FP, Garcia Ados S, Andrade DF, Werner CR, Carvalho T. Clinical and economic outcome of a cardiopulmonary and metabolic rehabilitation program. Arquivos Brasileiros de Cardiologia. 2007;88(3). | No economic analysis | |
| 76. | Reidlinger DP, Sanders TA, Goff LM. How expensive is a cardioprotective diet? Analysis from the CRESSIDA study. Public Health Nutrition. 2017;20(8). | No economic analysis | |
| 77. | Reynolds MR, Apruzzese P, Galper BZ, Murphy TP, Hirsch AT, Cutlip DE, et al. Cost-effectiveness of supervised exercise, stenting, and optimal medical care for claudication: results from the Claudication: Exercise Versus Endoluminal Revascularization (CLEVER) trial. Journal of the American Heart Association. 2014;3(6). | Non trial data analysis | |
| 78. | Richards A, Barkham M, Cahill J, Richards D, Williams C, Heywood P. PHASE: a randomised, controlled trial of supervised self-help cognitive behavioural therapy in primary care. British Journal of General Practice. 2003;53(495). | No economic analysis | |
| 79. | Ride J, Lorgelly P, Tran T, Wynter K, Rowe H, Fisher J. Preventing postnatal maternal mental health problems using a psychoeducational intervention: the cost-effectiveness of What Were We Thinking. BMJ Open. 2016;6(11). | Non trial data analysis | |
| 80. | Robbins JA. A home-based, nurse-delivered exercise program reduced falls and serious injuries in persons greater than or equal to 80 years of age. ACP Journal Club. 2001;135(3). | Wrong study type | |
| 81. | Robertson MC, Gardner MM, Devlin N, McGee R, Campbell AJ. Effectiveness and economic evaluation of a nurse delivered home exercise programme to prevent falls. 2: Controlled trial in multiple centres. BMJ. 2001;322(7288). | Wrong study type | |
| 82. | Schmidt C, Magalhaes S, Gois Basilio P, Gouveia M, Teixeira M, Santos C, et al. Home- versus centre-based EXercise InTervention in patients with Heart Failure (EXIT-HF trial): A pragmatic randomized controlled trial. Revista portuguesa de cardiologia : orgao oficial da Sociedade Portuguesa de Cardiologia = Portuguese journal of cardiology : an official journal of the Portuguese Society of Cardiology. 2024;43(3):149-58. | Wrong study type- “protocol” | |
| 83. | Seki Y, Nagata S, Shibuya T, Yoshinaga N, Yokoo M, Ibuki H, et al. A feasibility study of the clinical effectiveness and cost-effectiveness of individual cognitive behavioral therapy for panic disorder in a Japanese clinical setting: an uncontrolled pilot study. BMC Research Notes. 2016;9(1). | Non trial data analysis | |
| 84. | Sharples L, Glover M, Clutterbuck-James A, Bennett M, Jordan J, Chadwick R, et al. Clinical effectiveness and cost-effectiveness results from the randomised controlled Trial of Oral Mandibular Advancement Devices for Obstructive sleep apnoea-hypopnoea (TOMADO) and long-term economic analysis of oral devices and continuous positive airway pressure. Health Technology Assessment (Winchester, England). 2014;18(67). | Trial data is Duplicate | |
| 85. | Shigematsu R, Okura T, Sakai T, Rantanen T. Square-stepping exercise versus strength and balance training for fall risk factors. Aging-Clinical & Experimental Research. 2008;20(1). | No economic analysis | |
| 86. | Strong LL, Von Korff M, Saunders K, Moore JE. Cost-effectiveness of two self-care interventions to reduce disability associated with back pain. Spine (03622436). 2006;31(15). | Intervention not in HANDI | |
| 87. | Whigham L, Joyce T, Harper G, Irving PM, Staudacher HM, Whelan K, et al. Clinical effectiveness and economic costs of group versus one-to-one education for short-chain fermentable carbohydrate restriction (low FODMAP diet) in the management of irritable bowel syndrome. Journal of Human Nutrition & Dietetics. 2015;28(6). |  | |
| 88. | Wiles N, Thomas L, Abel A, Barnes M, Carroll F, Ridgway N, et al. Clinical effectiveness and cost-effectiveness of cognitive behavioural therapy as an adjunct to pharmacotherapy for treatment-resistant depression in primary care: the CoBalT randomised controlled trial. Health Technology Assessment (Winchester, England). 2014;18(31). | Duplicate | |
| 89. | Wiles NJ, Thomas L, Turner N, Garfield K, Kounali D, Campbell J, et al. Long-term effectiveness and cost-effectiveness of cognitive behavioural therapy as an adjunct to pharmacotherapy for treatment-resistant depression in primary care: follow-up of the CoBalT randomised controlled trial. The Lancet Psychiatry. 2016;3(2). | Non trial data analysis | |
| 90. | Witham MD, Fulton RL, Greig CA, Johnston DW, Lang CC, van der Pol M, et al. Efficacy and cost of an exercise program for functionally impaired older patients with heart failure: a randomized controlled trial. Circulation: Heart Failure. 2012;5(2). | No economic analysis | |
| 91. | Wright B, Tindall L, Scott AJ, Lee E, Cooper C, Biggs K, et al. One session treatment (OST) is equivalent to multi-session cognitive behavioral therapy (CBT) in children with specific phobias (ASPECT): Results from a national non-inferiority randomized controlled trial. Journal of Child Psychology and Psychiatry. 2023;64(1). | Intervention and condition not in HANDI | |
| 92. | Wright JH, Wright AS, Albano AM, Basco MR, Goldsmith LJ, Raffield T, et al. Computer-assisted cognitive therapy for depression: maintaining efficacy while reducing therapist time. American Journal of Psychiatry. 2005;162(6). | No economic analysis | |
| 93. | Yeung A, Wang F, Feng F, Zhang J, Cooper A, Hong L, et al. Outcomes of an online computerized cognitive behavioral treatment program for treating chinese patients with depression: A pilot study. Asian Journal of Psychiatry. 2018;38. | No economic analysis | |

**Supplementary Table 4** List of excluded studies from backwards/forwards citation analysis with reasons for exclusion (n = 44)

|  | **Reference** | **Reason to exclude** |
| --- | --- | --- |
| 1. | Anderson R, Ukoumunne OC, Sayal K, Phillips R, Taylor J, Spears M, et al. Cost-effectiveness of classroom-based cognitive behaviour therapy in reducing symptoms of depression in adolescents: a trial-based analysis. Journal of child psychology and psychiatry, and allied disciplines. 2014;55(12):1390-7. | Duplicate |
| 2. | Andersson E, Hedman E, Ljótsson B, Wikström M, Elveling E, Lindefors N, et al. Cost-effectiveness of internet-based cognitive behavior therapy for obsessive-compulsive disorder: results from a randomized controlled trial. Journal of Obsessive-Compulsive and Related Disorders. 2015;4(NA):47-53. | Condition not in HANDI |
| 3. | Andersson E, Ljótsson B, Smit F, Paxling B, Hedman E, Lindefors N, et al. Cost-effectiveness of internet-based cognitive behavior therapy for irritable bowel syndrome: results from a randomized controlled trial. BMC public health. 2011;11(1):215-. | Condition not in HANDI |
| 4. | Andersson G, Wagner B, Cuijpers P. ICBT for Depression. NA2016. p. 17-32. | Wrong study type (book chapter) |
| 5. | Antonuccio DO, Thomas M, Danton WG. A cost-effectiveness analysis of cognitive behavior therapy and fluoxetine (prozac) in the treatment of depression *. Behavior Therapy. 1997;28(2):187-210. | Wrong study type & non trial data |
| 6. | Ballenger JC. Cost-effectiveness of preventing depression in primary care patients: Randomised trial. Yearbook of Psychiatry and Applied Mental Health. 2007;2007(NA):263-NA. | Duplicate |
| 7. | Başer ÖÇ, Ay S, Evcik D. Cost-effectiveness analysis of chronic mechanical back pain treatment modalities. Turkish journal of physical medicine and rehabilitation. 2020;66(4):413-22. | Intervention not in HANDI |
| 8. | Benedetto V, Hill J, Harrison J. Cost effectiveness of fall prevention programmes for older adults. British journal of community nursing. 2022;27(11):530-3. | Wrong study type & non trial data |
| 9. | Buhrman M, Fredriksson A, Edstrom G, Shafiei D, Tarnqvist C, Ljótsson B, et al. Guided Internet-delivered cognitive behavioural therapy for chronic pain patients who have residual symptoms after rehabilitation treatment: randomized controlled trial. European journal of pain (London, England). 2012;17(5):753-65. | No economic analysis |
| 10. | Campbell AJ, Robertson MC, La Grow SJ, Kerse NM, Sanderson GF, Jacobs RJ, et al. Randomised controlled trial of prevention of falls in people aged > or =75 with severe visual impairment: the VIP trial. BMJ. 2005;331(7520). | Already excluded in original search-Intervention not in HANDI |
| 11. | Chisholm D, Godfrey E, Ridsdale L, Chalder T, King M, Seed PT, et al. Chronic fatigue in general practice: economic evaluation of counselling versus cognitive behaviour therapy. The British journal of general practice : the journal of the Royal College of General Practitioners. 2001;51(462):15-8. | Intervention not in HANDI |
| 12. | Church J, Haas M, Goodall S. Cost Effectiveness of Falls and Injury Prevention Strategies for Older Adults Living in Residential Aged Care Facilities. PharmacoEconomics. 2015;33(12):1301-10. | Non trial data analysis |
| 13. | Darlow B, Stanley J, Dean S, Abbott JH, Garrett S, Wilson R, et al. The Fear Reduction Exercised Early (FREE) approach to management of low back pain in general practice: A pragmatic cluster-randomised controlled trial. PLoS medicine. 2019;16(9):e1002897-NA. | Intervention not in HANDI |
| 14. | Dickerson JF. CBT cost effective in youth declining antidepressants. PharmacoEconomics & Outcomes News. 2018;795(1):7-. | Duplicate |
| 15. | Dukhovny D, Dennis C-L, Hodnett E, Weston J, Stewart DE, Mao W, et al. Prospective economic evaluation of a peer support intervention for prevention of postpartum depression among high-risk women in Ontario, Canada. American journal of perinatology. 2013;30(8):631-42. | Intervention not in HANDI |
| 16. | Edwards RT, Linck P, Hounsome N, Raisanen LM, Williams N, Moore L, et al. Cost-effectiveness of a national exercise referral programme for primary care patients in Wales: results of a randomised controlled trial. BMC public health. 2013;13(1):1021-. | Duplicate |
| 17. | Golmohammadi K, Jacobs P, Sin DD. Economic evaluation of a community-based pulmonary rehabilitation program for chronic obstructive pulmonary disease. Lung. 2004;182(3):187-96. | Wrong study type |
| 18. | Gräfe V, Berger T, Hautzinger M, Hohagen F, Lutz W, Meyer B, et al. Health economic evaluation of a web-based intervention for depression: the EVIDENT-trial, a randomized controlled study. Health economics review. 2019;9(1):16-. | No cost effectiveness outcomes |
| 19. | Gräfe V, Moritz S, Greiner W. Health economic evaluation of an internet intervention for depression (deprexis), a randomized controlled trial. Health economics review. 2020;10(1):1-11. | No cost effectiveness outcomes |
| 20. | Grønne DT, Roos EM, Ibsen R, Kjellberg J, Skou ST. Cost-effectiveness of an 8-week supervised education and exercise therapy programme for knee and hip osteoarthritis: a pre-post analysis of 16 255 patients participating in Good Life with osteoArthritis in Denmark (GLA:D). BMJ open. 2021;11(12):e049541-e. | Wrong study type |
| 21. | Hartfiel N, Clarke G, Havenhand JN, Phillips C, Edwards RT. Cost-effectiveness of yoga for managing musculoskeletal conditions in the workplace. Occupational medicine (Oxford, England). 2017;67(9):687-95. | Wrong study type/design |
| 22. | Heinrich S, Rapp K, Stuhldreher N, Rissmann U, Becker C, König HH. Cost-effectiveness of a multifactorial fall prevention program in nursing homes. Osteoporosis international : a journal established as result of cooperation between the European Foundation for Osteoporosis and the National Osteoporosis Foundation of the USA. 2012;24(4):1215-23. | Wrong study type |
| 23. | Hendriks MRC, Evers SMAA, Bleijlevens MHC, van Haastregt JCM, Crebolder HFJM, van Eijk JTM. Cost-effectiveness of a multidisciplinary fall prevention program in community-dwelling elderly people: a randomized controlled trial (ISRCTN 64716113). International journal of technology assessment in health care. 2008;24(2):193-202. | Intervention not in HANDI |
| 24. | Jacobs N, Evers SMAA, Ament AJHA, Claes N. Cost-utility of a cardiovascular prevention program in highly educated adults: Intermediate results of a randomized controlled trial. International journal of technology assessment in health care. 2010;26(1):11-9. | Intervention not strictly in HANDI |
| 25. | Katon W, Schoenbaum M, Fan MY, Callahan CM, Williams JW, Hunkeler EM, et al. Cost-effectiveness of improving primary care treatment of late-life depression. Archives of general psychiatry. 2005;62(12):1313-20. | Wrong study type/design |
| 26. | Kendrick T, Chatwin J, Dowrick C, Tylee A, Morriss R, Peveler R, et al. Randomised controlled trial to determine the clinical effectiveness and cost-effectiveness of selective serotonin reuptake inhibitors plus supportive care, versus supportive care alone, for mild to moderate depression with somatic symptoms in primary care: the THREAD (THREshold for AntiDepressant response) study. Health technology assessment (Winchester, England). 2009;13(22):1-159. | Intervention not in HANDI |
| 27. | Kühr EM, Ribeiro RA, Rohde LEP, Polanczyk CA. Cost-Effectiveness of Supervised Exercise Therapy in Heart Failure Patients. Value in health: the journal of the International Society for Pharmacoeconomics and Outcomes Research. 2011;14(5): S100-7. | Non trial data analysis |
| 28. | Lave JR, Frank RG, Schulberg HC, Kamlet MS. Cost-effectiveness of treatments for major depression in primary care practice. Archives of general psychiatry. 1998;55(7):645-51. | Intervention not in HANDI |
| 29. | Li J, Parrott S, Sweeting MJ, Farmer A, Ross J, Dack C, et al. Cost-effectiveness of facilitated access to a self-management website, compared to usual care, for patients with type 2 diabetes (help-diabetes): Randomized controlled trial. Journal of medical Internet research. 2018;20(6):1-15. | Intervention not specially in HANDI |
| 30. | Ljótsson B, Andersson G, Andersson E, Hedman E, Lindfors P, Andréewitch S, et al. Acceptability, effectiveness, and cost-effectiveness of internet-based exposure treatment for irritable bowel syndrome in a clinical sample: a randomized controlled trial. BMC gastroenterology. 2011;11(1):110-. | Intervention not in HANDI |
| 31. | Mar J, Rueda JR, Durán-Cantolla J, Schechter C, Chilcott J. The cost-effectiveness of nCPAP treatment in patients with moderate-to-severe obstructive sleep apnoea. The European respiratory journal. 2003;21(3):515-22. | Non trial data analysis |
| 32. | McCrone P, Knapp M, Kennedy T, Seed PT, Jones R, Darnley S, et al. Cost-effectiveness of cognitive behaviour therapy in addition to mebeverine for irritable bowel syndrome. European journal of gastroenterology & hepatology. 2008;20(4):255-63. | Intervention not in HANDI |
| 33. | McCrone P, Ridsdale L, Darbishire L, Seed PT. Cost-effectiveness of cognitive behavioural therapy, graded exercise and usual care for patients with chronic fatigue in primary care. Psychological medicine. 2004;34(6):991-9. | Wrong study design |
| 34. | Meng H, Friedberg F. Cost-utility of home-based fatigue self-management versus usual care for the treatment of chronic fatigue syndrome. Fatigue : biomedicine, health & behavior. 2017;5(4):202-14. | Intervention not in HANDI |
| 35. | Murray E, Ross J, Pal K, Li J, Dack C, Stevenson F, et al. A web-based self-management programme for people with type 2 diabetes: the HeLP-Diabetes research programme including RCT. Programme Grants for Applied Research. 2018;6(5):1-242. | Intervention not in HANDI |
| 36. | Nagaoka M, Koreki A, Kosugi T, Ninomiya A, Mimura M, Sado M. Economic Evaluation Alongside a Randomized Controlled Trial of Mindfulness-Based Cognitive Therapy in Healthy Adults. Psychology research and behavior management. 2023;16(NA):2767-85. | No specific condition as in healthy adults so not applicable to HANDI |
| 37. | Pachito DV, Eckeli AL, Drager LF. Cost-Utility Analysis of Continuous Positive Airway Pressure Therapy Compared With Usual Care for Obstructive Sleep Apnea in the Public Health System in Brazil. Value in health regional issues. 2023;40(NA):81-8. | Non trial data analysis |
| 38. | Pelle T, Bevers K, van den Hoogen FJA, van der Palen J, van den Ende CHM. Economic evaluation of the dr. Bart app in people with knee and/or hip osteoarthritis. Arthritis care & research. 2022;74(6):945-54. | Intervention not in HANDI |
| 39. | Pietzsch JB, Garner AM, Cipriano LE, Linehan JH. An integrated health-economic analysis of diagnostic and therapeutic strategies in the treatment of moderate-to-severe obstructive sleep apnea. Sleep. 2011;34(6):695-709. | Non trial data analysis |
| 40. | Ratcliffe J, Thomas K, MacPherson H, Brazier J. A randomised controlled trial of acupuncture care for persistent low back pain: cost effectiveness analysis. BMJ (Clinical research ed). 2006;333(7569):626-. | Intervention not in HANDI |
| 41. | Risør BW, Frydendal DH, Villemoes MK, Nielsen CP, Rask CU, Frostholm L. Cost Effectiveness of Internet-Delivered Acceptance and Commitment Therapy for Patients with Severe Health Anxiety: A Randomised Controlled Trial. PharmacoEconomics - open. 2022;6(2):179-92. | Intervention not in HANDI |
| 42. | Slade EG, JD.; Lu, W.; Yanos, PT.; Rosenberg, S.; Silverstein, SM.; Minsky SK.; Mueser, KT. Tailored CBT not cost effective versus brief intervention for PTSD. PharmacoEconomics & Outcomes News. 2017;783(1):32-. | Non primary care |
| 43. | Weatherly H, Griffin S, Mc Daid C, Durée KH, Davies RJO, Stradling J, et al. An economic analysis of continuous positive airway pressure for the treatment of obstructive sleep apnea-hypopnea syndrome. International journal of technology assessment in health care. 2009;25(1):26-34. | Non trial data analysis |
| 44. | Xin YD, A.; Briggs, A; McCombie, L.; Messow, CM.; Grieve, E.; Leslie, WS.; Taylor, R., Lean, MEJ. DiRECT weight management programme cost effective in T2DM. PharmacoEconomics & Outcomes News. 2020;860(1):18-. | Wrong study type |

**Supplementary Figure 1-**Cost effectiveness plane


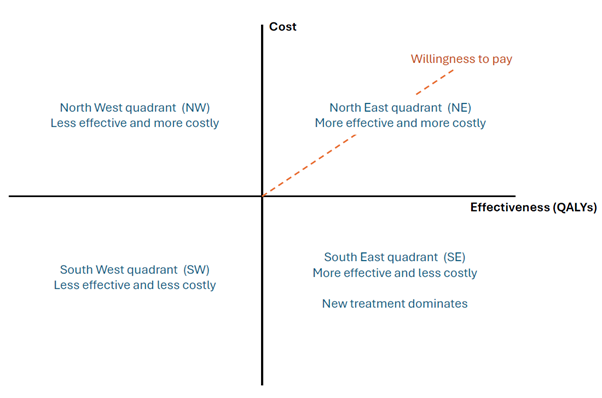


**Supplementary Table 5**. Additional characteristics of total included studies and total interventions including both CUA and CEA interventions

|  | Total Studies  N= 156  n (% of total studies) | Total interventions  N=176  N (% of total interventions) | CUA interventions  N=150  n (% of total interventions) | CEA interventions  N=25 + 1 CBA  N (% of total interventions) |
| --- | --- | --- | --- | --- |
| Comparator |  |  |  |  |
| Usual care/Usual GP care/TAU/Standard |  | 92  (52.3) | 77  (51.3) | 15  (8.5) |
| Control/waitlist control |  | 38  (21.6) | 35  (23.3) | 3  (1.7) |
| Active; Drug/surgery |  | 7  (4.0) | 5  (3.3) | 2  (1.1) |
| Active; non-drug/non-surgical |  | 39  (22.2) | 33  (22.0) | 6  (3.4) |
| Year |  |  |  |  |
| <2010 | 30 (19.2) | 30  (17.0) | 20  (13.3) | 10  (5.7) |
| >=2010 | 126 (80.8) | 146  (83.0) | 130  (86.7) | 16  (9.1) |

**Supplementary Table 6-** Additional characteristics of CUA interventions by ICUR quadrant

| **N=130 Total CUA studies and 150 CUA interventions** | | | | |
| --- | --- | --- | --- | --- |
| Total of CUA interventions  N (column %) of Total CUA intervention | Q 1a  n (row %) | Q 2b  n (row %) | Q 3c  n (row %) | Q 4d  n (row %) |
| Perspective  **N=150 (100.0)** | **N=60 (40%)** | **N=74 (49.3)** | **N=9 (6.0%)** | **N=7 (4.7)** |
| Societal  **N=68**  **(45.3)** | 37  (54.4) | 21  (30.9) | 5  (7.4) | 5  (7.4) |
| Health System  **N=60**  **(40.0)** | 14  (23.3) | 41  (68.3) | 3  (5.0) | 2  (3.3) |
| Gave both Societal & Health  **N=19**  **(12.7)** | 8  (42.1) | 10  (52.6) | 1  (5.3) | 0  (0.0) |
| Payer/Employer  **N=3**  **(2.0)** | 1  (33.3) | 2  (66.7) | 0  (0.0) | 0  (0.0) |
| Comparator  **N=150 (100.0)** | **N=60 (40%)** | **N=74 (49.3)** | **N=9 (6.0%)** | **N=7 (4.7)** |
| Usual care/Usual GP care/TAU/Standard  **N**=**77**  **(51.3)** | 28  (36.4) | 41  (53.2) | 4  (5.2) | 4  (5.2) |
| Control/waitlist control  **N=35**  **(23.3)** | 14  (40.0) | 21  (60.0) | 0  (0.0) | 0  (0.0) |
| Active; Drug/surgery  **N=5**  **(3.3)** | 2  (40.0) | 1  (20.0) | 2  (40.0) | 0  (0.0) |
| Active; non-drug/non-surgical  **N=33**  **(22.0)** | 16 (48.5) | 11  (33.3) | 3  (9.1) | 3  (9.1) |
| Year  **N=150 (100.0)** | **N=60 (40%)** | **N=74 (49.3)** | **N=9 (6.0%)** | **N=7 (4.7)** |
| <2010  **N=20**  **(13.3)** | 9  (45.0) | 8  (40.0) | 1  (5.0) | 2  (10.0) |
| >=2010  **N=130**  **(86.7)** | 51  (39.2) | 66  (50.8) | 8  (6.2) | 5  (3.8) |
| Currency  **N=150 (100.0)** | **N=60 (40%)** | **N=74 (49.3)** | **N=9 (6.0%)** | **N=7 (4.7)** |
| EUR  **N=60**  **(40.0)** | 21  (35.0) | 31  (51.7) | 4  (6.7) | 4  (6.7) |
| GBP  **N=44**  **(29.3)** | 19  (43.2) | 20  (45.5) | 2  (4.5) | 3  (6.8) |
| USD  **N=28**  **(18.7)** | 13  (46.4) | 12  (42.9) | 3  (10.7) | 0  (0.0) |
| CAD/AUD/NZD  **N=16**  **(10.7)** | 7  (43.8) | 9  (56.3) | 0  (0.0) | 0  (0.0) |
| Other; CNY/SGD  **N=2**  **(1.3)** | 0  (0.0) | 2  (100.00) | 0  (0.0) | 0  (0.0) |
| Region  **N=150 (100.0)** | **N=60 (40%)** | **N=74 (49.3)** | **N=9 (6.0%)** | **N=7 (4.7)** |
| Europe  **N=109**  **(72.7)** | 42  (38.5) | 53  (48.6) | 7  (6.4) | 7  (6.4) |
| North America  **N=26**  **(17.3)** | 13  (50.0) | 11  (42.3) | 2  (7.7) | 0  (0.0) |
| South America  **N=2**  **(1.3)** | 1  (50.0) | 1  (50.0) | 0  (0.0) | 0  (0.0) |
| Asia  **N=5**  **(3.3)** | 2  (1.8) | 3  (2.9) | 0  (0.0) | 0  (0.0) |
| **Oceania**  **N=8**  **(5.3)** | 2  (25.0) | 6  (75.0) | 0  (0.0) | 0  (0.0) |
| **Type of Analysis**  **N=150 (100.0)** | **N=60 (40%)** | **N=74 (49.3)** | **N=9 (6.0%)** | **N=7 (4.7)** |
| CUA only  **71**  **(47.3)** | 26  (36.6) | 39  (54.9) | 4  (5.6) | 2  (2.8) |
| CUA and CEA  **79**  **(52.7)** | 34  (43.0) | 35  (44.3) | 5  (6.3) | 5  (6.3) |

^a^Q1= Interventions that cost less and are more effective

^b^Q2=Interventions that cost more and are more effective

^c^Q3= Interventions that cost less and are less effective

^d^Q4= Interventions that cost more and are less effective

^#^ Study may have more than one intervention but only utilised one

^d^Device refers to a type of intervention that uses a specific device such as a splint, walking cane, pedometer, or mandibular device.

Abbreviations; CUA=Cost Utility Analysis, CEA=Cost effectiveness Analysis, CBA=Cost Benefit Analysis, EUR= EURO currency, GBP= Great British Pound, CAD=Canadian Dollar, USD=United States Dollar, AUD=Australian Dollar, NZD=New Zealand Dollar, CNY= Chinese Yuan, SGD=Singapore Dollar

**Supplementary Figure 2**.

**
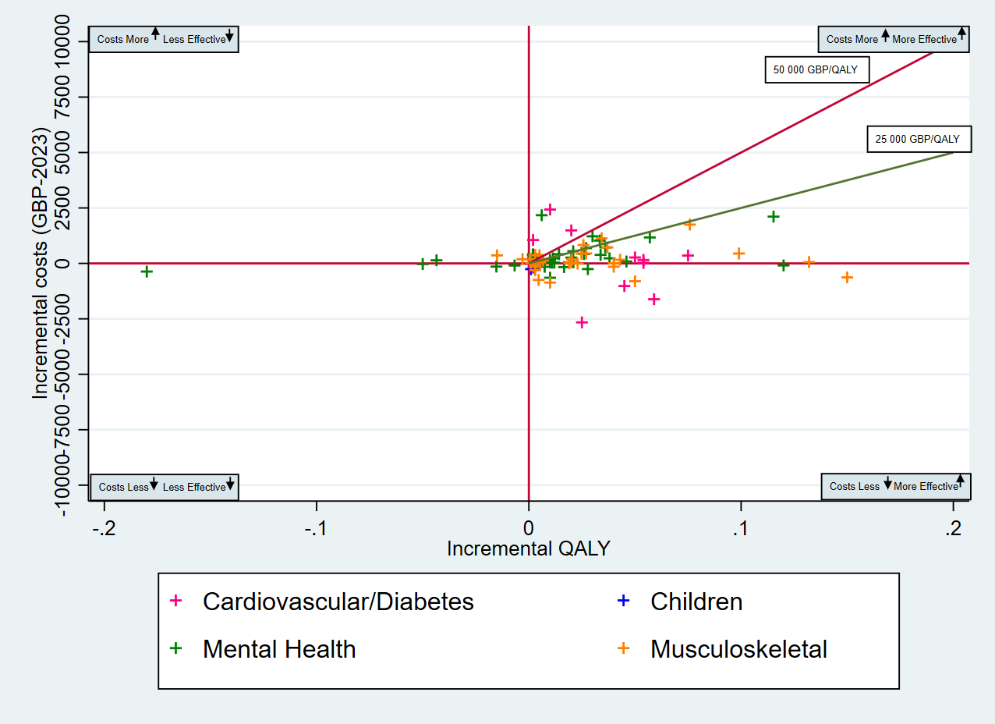
**

**Supplementary Figure 2**. Cost utility analysis of HANDI interventions based on the Health System perspective with all costs converted to GBP 2023. The outlier study by Yu 2004(1) is not shown.

**Supplementary Figure 3**.


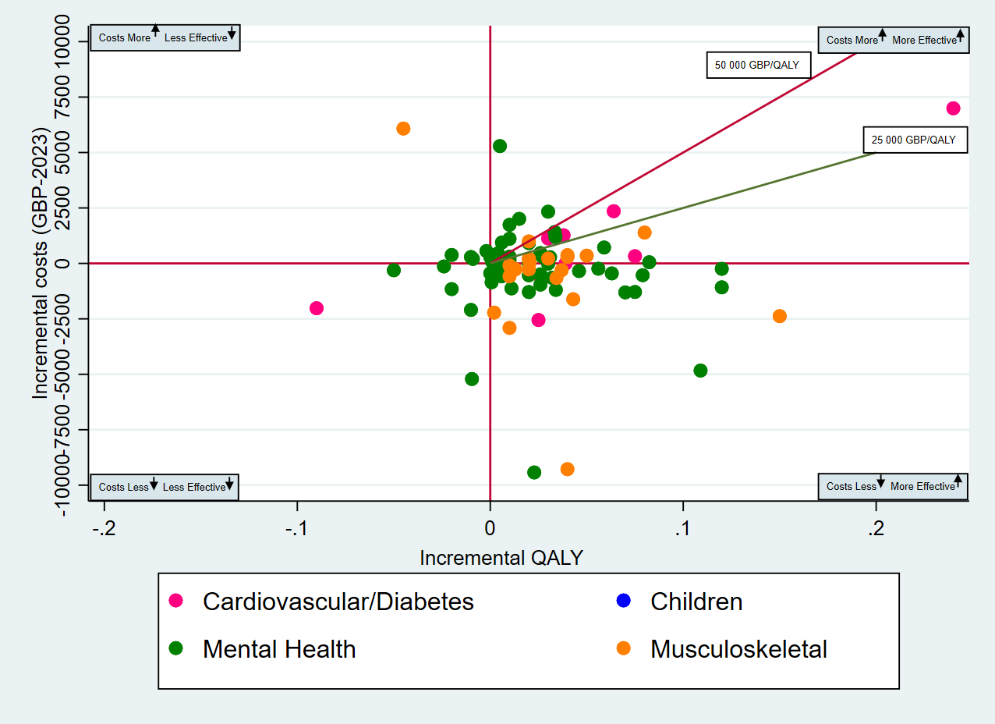


**Supplementary Figure 3.** Cost utility analysis of HANDI interventions based on the Societal perspective with all costs converted to GBP 2023. The outlier studies by Patrick 2001(2) and Barnhofer 2025(3) are not shown.

**Supplementary Table 7.** Characteristics of studies and CUA Interventions that cost less and are more effective (n= 60 interventions)

| **Study, Year, Country** | **Category (condition); Population** | **Intervention type; Intervention vs Comparator** | **Outcomes**  **(Time Horizon)** | **Perspective; Analysis; Setting** | **Results (Incremental costs (IC); QALY gained/health outcome; ICER/ICUR)**  (Currency, price year) | **Graph details; Perspective, Intervention^a^**  **ICUR^b^ (costs in GBP 2023)** |
| --- | --- | --- | --- | --- | --- | --- |
| Abbott, 2019, New Zealand | **Musculoskeletal** (Hip or Knee OA); 206 adult patients with hip or knee OA. | **Exercise/Physical Therapy**  Exercise vs UC  Manual physio vs UC  Combined vs UC | QALY; Pain and Function-WOMAC  (2 years) | Societal & Health System; CUA; Primary Care/Outpatient care | **Exercise vs UC**:  **Societal** CUA IC –3530 QALY 0.15 ICUR –23533 (reported –16616)  **Health** CUA IC -935 QALY 0.15 ICUR -6233 (reported -3657)  (NZD, 2009) | **Societal**  **Exercise vs UC**  -15855.267  (-2378.29)  **Health**  **Exercise vs UC**  -4199.5996  (-629.94) |
| Adjetey, 2023, Canada | **Cardiovascular** (Stroke); 120 adults 55+ who had experienced a stroke at least 12 months prior. | **Exercise/Physical** **Therapy**  Exercise program (strength, aerobic, and balance) vs control (balance and tone program) or cognitive and social enrichment activities program vs control (balance and tone program) | QALY; change in ADAS-Cog-Plus  (6 & 12 months) | Health System; CEA & CUA; community | **Exercise vs control**  **6 months CUA** IC -32 QALY +0.00946 ICUR -3381  **12 months CUA** IC -333 QALY +0.00215956; ICUR –154198  (CAD, 2022) | **Health**    **Exercise vs control**    **12 months**  -89476.555  (-193.23) |
| Axelsson, 2018, Sweden | **Mental health** (Health Anxiety);  132 adults 18+ years with severe health anxiety. | **Behavioural**  3 treatments vs waitlist control:  Bibliotherapy (self-guided CBT with booklet)  Internet CBT (with therapist guided support); Internet CBT (without therapist guided support) | QALY; change in health anxiety-HAI (% in remission)  (12 weeks) | Societal & Health system; CEA & CUA; Hospital was trial centre | **Societal**  **Bibliotherapy CBT vs control**  **CUA**; IC 128 QALY 0.0053 ICUR 24 245  **Guided I-CBT vs control**  **CUA**; IC 208 QALY 0.0020 ICUR 103 048  **Unguided I-CBT vs control**  **CUA**; IC -57 QALY 0.0034 ICUR -16 951  **Health**  **Bibliotherapy CBT vs control**  **CUA** IC 54 QALY 0.0053 ICUR 10189  **Guided I-CBT vs control**  **CUA** IC 320 QALY 0.0020 ICUR 160000  **Unguided I-CBT vs control**  **CUA** IC 34 QALY 0.0034 ICUR 10000  (GBP, 2014) | **Societal**    **Unguided internet CBT vs control**   -21509  (-73.13) |
| Baka, 2021, Netherlands | **Mental health** (Insomnia);  134 adults 18+ who met the DSM-5 criteria for insomnia disorder. | **Behavioural**  Guided internet -delivered CBT vs TAU | QALY; change in ISI (insomnia severity index)   (26 weeks) | Societal & Health system (Sensitivity analysis); CEA & CUA; Primary care | **Societal**  CEA IC-318 ISI 3.91 ICER -81  CUA IC -318 QALY 0.0113 ICUR -28056  **Health**  CEA IC 217 ISI 3.91 ICER 55  CUA IC 217 QALY 0.01 ICUR 20257   (EUR, 2018) | **Societal**  -29406  (-333.3) |
| Barnhofer, 2025, UK | **Mental health** (Depression); 166 adults with current major depressive disorder (PHQ-9 score >=10 after at least 12 sessions of “Talking Therapies” | **Behavioural**  Mindfulness-based cognitive therapy (MBCT) vs TAU | QALY (34 weeks) | Societal; CUA; Primary care | **CUA**  IC -245.23 QALY 0.564 ICUR -434.80  (GBP, 2021-2022) | **Societal**  -485.24  (-259.63) |
| Bower, 2000, United Kingdom | **Mental Health** (Anxiety and or Depression);  197 patients presenting with depression or mixed anxiety and depression. | **Behavioural**  CBT vs usual GP care  Non-directive counselling vs usual GP care | QALY; Severity of depression-BDI   (12 months) | Societal; CEA & CUA; Primary Care | **CBT vs usual GP care**  **CEA** IC -157 BDI NR ICER NR  **CUA** IC -157 QALY NR (but not significantly different) ICUR NR  (GBP 1997/98) | ICUR not graphed  (286.31) |
| Brettschneider, 2020, Germany | **Mental Health** (Panic disorder);  419 Adults 18+ with a diagnosis of panic disorder with or without agoraphobia. | **Behavioural**  Individual CBT with therapist vs Usual GP care  (PARADISE study) | QALY  (12 months) | Societal; CUA; Primary Care | **CUA** IC -1017 QALY +0.034 ICUR intervention dominated (less costly and more effective)  (EUR, 2012) | **Societal**  -35146.5  (-1194.98) |
| Buntrock, 2021, Germany | **Mental Health**  (Insomnia); 128 schoolteachers aged 18+ with clinically significant insomnia symptoms (ISI>14) and elevated work- related rumination (Irritation scale, subscale "Cognitive Irritation">14) | **Behavioural**  Internet CBT with therapist guided support (GET.ON Recovery) + usual GP care vs Usual GP care waitlist control | QALY; insomnia free-ISI  (6 months) | Societal & Health system; CEA & CUA; Community | **Societal**  **CEU** IC -1121 ISI +0.30 ICER Dominant  **CUA** IC -1121 QALY +0.02 ICUR Dominant  **Health**  **CEA** IC 225.7 ISI +0.30 ICER 650  **CUA** IC 225.7 QALY +0.02 ICUR 11 285  (EUR, 2013) | **Societal**  -64590  (-1292) |
| Burge, 2020, Australia | **Cardiovascular** (COPD);  166 adult patients with stable COPD | **Exercise/Physical Therapy**  Home-based exercise rehab + Usual GP care vs Usual GP care | QALY; distance walked on 6 min walk test  (12 months) | Societal & Health System  ; CEA & CUA; Hospital | **Societal**  **CEU** IC -4316 HC  6MWD +14 ICER NR  **CUA** IC -4316 QALY +0.025 ICUR NR  **Health**  **CEU** IC –4497 6MWD +14 ICER NR  **CUA** IC –4497 QALY +0.025 ICUR NR  (AUD, 2017) | **Societal**  -102110  (-2553)  **Health**   -106392  (-2660) |
| Chuang, 2012, United Kingdom | **Musculoskeletal**  (Chronic low back pain) 313 patients aged 18 to 65 years who had consulted their general practitioner with low back pain in the last 18 months. | **Exercise/Physical Therapy**  Yoga vs usual care | QALY (12 months) | Societal & Health System; CUA; Primary care | **Societal**  **CUA** IC -213.9 QALY +0.037 ICUR Dominant  **Health**  **CUA** IC +506.8 QALY +0.037 ICUR 13606  (GBP, 2008/09) | **Societal**  **-**8222  (-304) |
| Cochrane, 2005, United Kingdom | **Musculoskeletal**  (OA Hip or Knee) 312  patients >60 years with confirmed hip and/or knee OA. | **Exercise/Physical Therapy**  Group water-based exercise vs control | QALY; change in WOMAC   (12 months) | Societal; CEA, CUA & CBA; Community | **CEA;** IC -158 WOMAC +0.89 ICER 170  **CUA;** IC -158 QALY (-0.0315) +0.013 ICUR reported **mean ICUR** 5008/ calculated (-12154)  (GBP, 2002/03) | **Societal**  -20408  (-265) |
| Creswell, 2017, United Kingdom | **Mental Health**  (Anxiety); 136 children 5-12 years with anxiety associated with clinical impairment as the primary presenting problem. | **Behavioural**  Brief guided parent-delivered CBT vs solution-focused brief therapy | QALY; change in anxiety-CGI-1   (6 months) | Societal; CEA & CUA;  Allied Health | **CEA;** IC -448 CGI-1 0.008 ICER NR  **CUA**; IC -448 QALY 0.006 ICUR NR (-74667  (GBP, 2013/14) | **Societal**  -97020  (-582) |
| Davis, 2011, Canada | **Musculoskeletal**  (Falls) 155 Women aged 65 to 75 years from the community. | **Exercise/Physical Therapy**  **3 arms;**  Resistance exercise 1 per week vs balance & tone 2x week  Resistance exercise 2 x per week vs balance & tone 2x week | QALY-*SF-6D; falls prevented  (9 months) | Health system; CEA & CUA; Community | **Resistance 1x week**  **CEA** IC -393 falls prevented -8 ICER dominates  **CUA** IC -393 QALY 0.003 ICUR dominates  **Resistance 2x week**  **CEA** IC -88 falls prevented -6 ICER Dominates (-131000)  **CUA** IC -88 QALY 0.003 ICUR Dominates (-29333)  (CAD, 2008) | **Health**    **Resistance 1x**  -102063  (-306)    **Resistance 2x**  **-**22853  (-69) |
| Davis, 2020, Canada | **Musculoskeletal**  (Falls) 344 adults aged 70 or older who sustained a fall in the past 12 month | **Exercise/Physical Therapy**  Otago Exercise Program +UC vs UC | QALY-*SF-6D; falls prevented   (12 months) | Health system; CEA & CUA; Community | **CEA** IC-217 falls prevented -130 ICER 2  **CUA** IC -217 QALY +0.003  ICUR -68584  (CAD, 2019) | **Health**  -48960  (-147) |
| Davis, 2022, Canada | **Musculoskeletal**  (Falls) 344 adults aged 70 or older who sustained a fall in the past 12 month. | **Exercise/Physical Therapy**  Otago Exercise Program + UC vs UC | QALY; falls prevented   (12 months) | Health system; CEA & CUA; Community; (**stratified by gender**) | **Women**  **CEA** IC 26 fall avoided -0.6 ICER -42  **CUA** IC 26 QALY +0.001 ICUR 28582  **Men**  **CEA** IC -1104 fall prevented -1.2 ICER 920 (dominates)  **CUA** IC -1104 QALY +0.0046 ICUR 238913 (CAD, 2019) | **Health**    **Men**  -162448  (-747) |
| de Bruin, 2016, Netherlands | **Mental Health**  (Insomnia) 62 Adolescents between 12-19 years meeting the diagnostic criteria of the SDM-IV-TR for primary insomnia. | **Behavioural**  Internet CBT with therapist vs Group face to face CBT | QALY; sleep efficiency>=85% (SE)  (12 months) | Societal; CEA & CUA; Community/Primary Care | **CEA** IC -405.55 SE>85% -4% ICER 12572  **CUA** IC -405.55 QALY 0.00 ICUR Dominant  (EUR, 2014) | **Societal**  ICUR undefined  (-445) |
| Dickerson, 2018, United States | **Mental Health**  (Depression) 212 Adolescents aged 12 to 18 with depression (diagnosis of major depression) identified in primary care settings and not taking antidepressants. | **Behavioural**  CBT with therapist vs Usual GP care | QALY; Depression free days-(DFD)  (12 months & 24 months) | Societal; CEA & CUA; Primary Care | **24- month results;**  **CEU** IC -4976 DFD +43.3 ICER -115  **CUA** IC -4976 QALY +0.109  ICUR -45792  (USD, 2008) | **Societal**    **24 months**  -44324  (-4831) |
| Frederix, 2015,  Belgium | **Cardiovascular** (CAD)  140 adults with CAD and entered cardiac rehabilitation. | **Exercise/Physical Therapy**  Internet-based telerehabilitation programme +conventional centre based cardiac rehab vs conventional cardiac rehab | QALY  (24 weeks) | Societal and patient; CUA; Primary Care/outpatients | CUA IC -564.40 QALY 0.026 ICUR -21707   (EUR, 2015) | **Societal**  -23006  (-598) |
| Ginsburg, 2022, United States | **Mental Health**  (Anxiety)” STARS” RCT with 216 students aged 6 to 18 years diagnosed with anxiety based on the ADIS. | **Behavioural**  School based modular CBT vs school clinician care | QALY  (12 months) | Societal; CUA; Community | **CUA** IC -166 QALY -0.024 ICUR 6917  *also did severity of anxiety subgroup  **Severe Anxiety subgroup;**  **CUA** IC -594 QALY +0.026 ICUR -22846  (USD, 2018) | **Societal**  **severe anxiety**  **subgroup**   -18981  (-494) |
| Goossens, 2015, Netherlands | **Musculoskeletal**  (chronic low back pain) 85 Patients   18-65 with lower back pain lasting at least 3 months with sufficiently disabled (Roland Disability score>3) and at least moderately fearful of movement, injury or reinjury (Tampa Scale of Kinesiophobia>33) | **Behavioural**  Exposure in vivo (CBT for chronic back pain) vs  Graded activity | QALY; self-reported disability via the Quebec back pain disability scale-QBPDS  (12 months) | Societal; CEA & CUA;  Outpatient/ Hospital | **CEA** IC -2643 QBPDS -2.23 ICER NR  **CUA** IC -2643 QALY +0.01 ICUR NR   (-264300)  (EUR, 2014) | **Societal**  -290884  (-2909) |
| Groessl, 2020, Netherlands | **Musculoskeletal**  (chronic low back pain) 150 Military veterans with chronic low back pain (defined as lasting 12+weeks). | **Exercise/Physical Therapy**  Yoga vs Waitlist control | QALY  (12 months) | Societal & Health system; CUA; Hospital is the trial centre | **Societal**  **CUA** IC -1899 QALY +0.043 ICUR Dominant (-44163)  **Health**  **CUA** IC 193 QALY 0.043 ICUR 4488  (USD, 2017) | **Societal**  -37573  (-1616) |
| Hautala, 2017, Finland | **Cardiovascular**  **(CAD with ACS)** 204 patients with coronary artery disease (CAD) who suffered from acute coronary syndrome (ACS) and underwent coronary angiography to confirm CAD. | **Exercise/Physical Therapy**  Exercise-based cardiac rehabilitation vs Usual GP care | QALY  (12 months) | Health system; CUA; Outpatient | **CUA** IC -1103 QALY +0.045 ICUR -24,511  (EUR, 2015) | **Health**  **-**22702  (-1022) |
| Hedman, 2011, Sweden | **Mental Health**  (Social Anxiety Disorder-SAD) 126 adult patients with a confirmed diagnosis of SAD as assessed using the Structured Clinical Interview for DSM-IV axis I disorders | **Behavioural**  Internet CBT with therapist support vs group F2F CBT | QALY; % with improvement in SAS-LSAS  (6 months) | Societal; CEA & CUA; Outpatient | **CEA** IC -1335 % improvement +0.19 ICER -7046  **CUA** IC -1335 QALY +0.075 ICUR -17823  (USD, 2009) | **Societal**  -17172  (-1288) |
| Hedman, 2013, Sweden | **Mental Health**  (Severe Health Anxiety) 81 participants with a primary diagnosis of severe health anxiety according to DSM-IV as assessed using the Health Anxiety Interview (HAI) | **Behavioural**  Internet CBT with therapist support vs control | QALY; % with improvement in Health Anxiety-HA  (12 months) | Societal; CEA & CUA; Hospital as trial centre | CEA IC -784 % improvement in HA +0.63   ICER -1244  CUA IC -784 QALY  +0.12 ICUR -6533  (GBP, 2010) | **Societal**  -8989  (-1079) |
| Horrell, 2014, United Kingdom | **Mental Health**  (Depression)   459 Adults 18+ with depression as indicated by the Beck depression Inventory | **Behavioural**  1 day CBT self-confidence Workshop vs Waiting list control | QALY; Depression free days (DFD) based on BDI-II<10  (12 weeks) | Societal; CEA & CUA; Community | **CEA** IC -7 DFD +19.23 ICER -0.36  **CUA** IC -7 QALY +0.03 ICUR -233  (GBP, 2010/11) | **Societal**  -321  (-9.63) |
| Jolstedt, 2018, Sweden | **Mental Health**  (Anxiety)  131 children 8-12 years with a principal anxiety disorder diagnosis | **Behavioural**  Internet CBT with therapist support vs Internet-delivered child-directed play (targeted at parent) | QALY; remission based on CSR-Clinician rated symptom severity  (6 months) | Societal; CEA & CUA; Hospital as trial centre | **CEA** IC -493.05 remission +0.34  ICER -1436  **CUA** IC -493.05 QALY +0.02 ICUR -24650  (EUR, 2016) | **Societal**  -26802  (-536) |
| Kahlke, 2023, Germany | **Mental Health**   (Social anxiety disorder) 200 adults 18+ and scored >21 on the Social Phobia Scale (SPS and/or >32 on Social Interaction Anxiety Scale (SIAS) and met criteria of SAD based on SCID-I (DSM-IV) | **Behavioural**  StudiCare (text-based CBT) vs waitlist control | QALY; Symptom free status SPS & SIAS   (6 months) | Societal & Health System; CEA & CUA; Primary care | **Societal**   CEA IC -321 SPS 0.26 ICER dominant   CUA IC -319 QALY 0.046 ICUR Dominant~-6978  **Health**  CEA IC 81 SPS 0.255 ICER 348  CUA IC 81 QALY 0.046 ICUR 1945  (EUR, 2017) | **Societal**  -7488  (-345) |
| Killingmo, 2023, Norway | **Musculoskeletal**  (Knee OA)  161 Adults with knee OA | **Exercise/Physical Therapy**  Strength exercise (SE) vs UC  Aerobic exercise (AE) vs UC | QALY (1 year) | Health & Societal; CUA, Community | **Societal**  **SE to UC;** IC -9445 QALY 0.05 ICUR  -188 900  **AE vs UC;** IC -9297 QALY 0.04 ICUR  -232425  **Health**  **SE to UC;** IC -805 QALY 0.05 ICU  -16100  **AE vs UC;** IC 349 QALY 0.04 ICUR 8725  (EUR, 2020) | **Societal**  SE vs UC  -188 526.97  (-9426.35)  AE vs UC  -231 966.01  (-9278.64)  **Health**  SE to UC  -16068.21  (-803.41) |
| Kloek, 2018, Netherlands | **Musculoskeletal**  (OA Hip or Knee)  207 patients with osteoarthritis of hip and/or knee and attending a physiotherapist | **Exercise/Physical Therapy**  E-exercise (F2F physio + web app) vs usual physiotherapy | QALY; physical functioning-HOOS/KOOS  (12 months) | Societal & Health System; CEA & CUA; Allied Health | **SOCIETAL;**  **CEA** IC -529 HOOS/KOOS +1.49 ICER -355  **CUA** IC -529 QALY +0.01 ICUR -52900  **Health**  CEA IC -792 HOOS/KOOS +1.49 ICER -532  **CUA** IC -792 QALY +0.01 ICUR -79200  (EUR, 2015) | **Societal**  -57774  (-578)    **Health**  -86497  (-865) |
| Lai, 2024, China | **Cardiovascular**   (to prevent high BP)  1612 participants from 48 facilities 55+ yrs with blood pressure measured at baseline. | **Nutrition**  Salt substitution + salt supply restriction vs salt substitution only vs salt supply restriction vs no strategy | QALY; reduction in SBP  (12 & 24 months) | Societal; CEA & CUA; Primary care | **12 months**; salt sub vs regular salt   CEA IC-9.77 SBP 7.41 ICER -1.32  CUA IC -9.77 QALY 0.039 ICUR -250.51  **24 months**; CEA IC -25.95 SBP +7.14 ICER -3.63   CUA IC-25.95 QALY 0.093 ICUR -279.03   (USD, 2020) | **Societal**  **12 months**  **salt sub vs regular salt**   -202  (-7.87) |
| Le, 2014, United States | **Mental Health**  (PTSD) 103 Adults aged 18 to 65 years with PTSD diagnosis based on DSM-IV criteria (The Optimizing PTSD Treatment Trial) | **Behavioural**  Prolonged exposure therapy vs Pharmacotherapy with Sertraline | QALY  (12 months) | Societal; CUA; University Hospital as trial centres | **CUA** IC -262 QALY +0.056 ICUR -4678.57  (USD, 2012) | **Societal**  -4289  (-240) |
| Li, 2019, USA | **Musculoskeletal**   (Falls) 670 community-dwelling adults 70+ who have fallen at least once in the preceding 12 months and at risk of falls based on referral. | **Exercise/Physical Therapy**   3 active group interventions; Tai Ji Quan vs Multimodal vs Stretching | QALY; incidence of falls  (6 months) | Health care system; CEA & CUA; Primary care | **TAI Ji vs stretching**  CEA IC-171 falls 0.95 ICER -180  CUA IC -171 QALY 0.04 ICUR -4269  **Tai ji vs multimodal**  CEA IC -723 falls 0.30 ICER -2410  CUA IC -723 QALY 0.02 ICER -36151  (USD, 2016-17) | **Health**    **Tai ji vs stretching**  **-**3706  (-148) |
| Lynch, 2021, USA | **Mental Health** (Anxiety and/or Depression)  185 youths 8-17 years with anxiety and/or depression | **Behavioural**  Brief behavioural therapy (BBT) vs assisted referral to community outpatient mental health care (ARC) | QALY; anxiety free days (AFDs) Depression-free days (DFDs)  (32 weeks) | Societal; CEA & CUA; Primary care | CEA IC -1082 AFDs 28.63 ICER -38 CEA IC -1082 DFDs 10.52 ICER -102  CUA IC -1082 QALY 0.026 ICUR -41414  (USD, 2014) | **Societal**  -36805  (-957) |
| McCarthy, 2004, United Kingdom | **Musculoskeletal**  (OA Knee) 214 Adults meeting the American College of Rheumatology’s classification of knee osteoarthritis, were selected from referrals from the primary and secondary care settings. | **Exercise/Physical Therapy**  class-based exercise programme + home-based exercise programme vs home-based exercise programme | QALY  (12 months) | Health System; CUA; Outpatient/Allied Health Primary Care/ Community | **CUA** IC -5.48 QALY +0.023 ICUR -238.26  (GBP, 1999/2000) | **Health**  -422  (-9.70) |
| McCrone, 2004, United Kingdom | **Mental Health**  (Depression and/or anxiety) 261 Patients aged 18–75 years with a diagnosis of depression and/or anxiety and were not currently receiving face-to-face psychological therapy (including counselling). | **Behavioural**  computerised CBT (beating the blues) + Usual GP care vs Usual GP care | QALY; Depression free days (DFD)  (8 months) | Societal; CEA & CUA; Primary Care | **CEU** IC -367 DFD +28.4 ICER -12.9  **CUA** IC -367 QALY +0.032 ICUR -11468.75  (GBP, 1999-2000) | **Societal**  -20305  (-650) |
| McCrone, 2012, UK | **Musculoskeletal**   (Chronic fatigue syndrome)  640 Adults 18 + attending outpatient CFS clinics. | **Exercise/Physical Therapy** Adaptive pacing therapy (APT), Cognitive behaviour therapy (CBT), or Graded exercise therapy (GET) vs Specialist medical care (SMC) | QALY; fatigue ~also had ICER based on disability  (12 months) | Societal & Health care System; CEA & CUA; Primary care | **GET vs SMC**  **Health**  **CEA** IC 837 fatigue 14.0 ICER 5987  **CUA** IC 810 QALY 0.0343 ICUR 23615  **Societal**  **CEA** IC -400 fatigue 14.0 ICER dominant  **CUA** IC -472 QALY 0.0343 ICUR -13761 dominant  (GBP 2009/2010) | **Societal**    **GET vs SMC**  **-**19203.50  (-658.68) |
| McMillan, 2015, United Kingdom | **Cardiovascular**  (Obstructive sleep apnoea) 278 adult aged ≥ 65 years with newly diagnosed OSAS [defined as oxygen desaturation index at ≥4% desaturation threshold level for > 7.5 events/hour and Epworth Sleepiness Scale (ESS) score of ≥ 9] (PREDICT trial) | **Device**  CPAP (continuous positive airway pressure) + Best supportive care vs Best supportive care | QALY  (12 months) | Health System; CUA; Hospital | **CUA** IC -35 QALY +0.005 ICUR NR (-7000)  (GBP, 2011-2012, discounted 3.5%) | **Health**  **-**9430  (-47.15) |
| Miyamoto, 2018, Brazil | **Musculoskeletal**   (Low back pain) 296 patients 18 to 80 yrs with non-specific chronic low back pain (NSCLBP) | **Exercise/Physical Therapy** Pilates -3 different doses (P1= 1/wk P2=2/wk P3=3/wk)  vs control (booklet) | QALY   (12 months) | Societal; CEA & CUA; Primary care/Allied health | **P1 vs booklet** CUA IC -75 QALY 0.01 ICUR -7008  **P2 vs booklet** CUA IC 174 QALY 0.02 ICUR 7053  **P3 vs booklet** CUA IC 230 QALY 0.04 ICUR  5503  (GBP, 2016) | **Societal**    **P1** -7008 |
| Moffett, 1999, USA | **Musculoskeletal**   (Low back pain)  187 patients 18 to 60 years with mechanical low back pain of 4 weeks to 6 months | **Exercise/Physical Therapy**  Exercise programme vs control | EuroQoL health index (EQ-5D); Roland disability questionnaire, Aberdeen back pain scale   (12 months) | Societal; CEA & CUA; Primary care | CEA IC -148.28 Roland disability -1.42 ICER 104.4 CEA IC -148.28 Aberdeen back pain scale -4.44 ICER 33.4  CUA IC -148.28 EQ-5D 0.02 ICUR -7414   (GBP, 1996*) | S**ocietal**  -13542  (-271) |
| Monteiro, 2022, Portugal | **Mental Health** (Postpartum Depression) 367 women 18+ present low risk for postpartum depression | **Behavioural**  "Be a Mom" web-based self-guided CBT vs waitlist control | QALY  (14 months) | Societal, CUA; Primary care | CUA IC -165.47 QALY 0.0064 ICER Dominant~-25855  (EUR, 2020, discount rate 3.5% (0.3 % per month) | **Societal**  -32733  (-209.49) |
| Morrell, 2009, United Kingdom | **Mental Health**  (Postnatal depression) 63 clusters and 418 at risk women on collaborating health visitors (HV’s) caseload for 4 months postnatally (The PoNDER trial) | **Behavioural**  CBT vs UC  PC approach vs UC | QALY  (6 months) | Health System; CUA; Primary Care | **CBT vs UC**    **ICUR** IC -45 QALY +0.004 ICUR NR  (-11250)  (GBP, 2003/04) | **Health**    **CBT vs UC**  -18425  (-73.70) |
| Morriss, 2019, United Kingdom | **Mental Health**  (Health Anxiety) 156 Participants were aged ≥18 years with ≥2 unscheduled healthcare contacts within 12 months and scored >18 on the Health Anxiety Inventory, HAI | **Behavioural**  Internet CBT with therapist (using videoconferencing or telephone) + Usual GP care vs Usual GP care | QALY  (12 months) | Societal; CUA; Primary Care/Hospital | **ICUR** IC -1064 QALY +0.07 ICUR -15200  (GBP, 2017) | **Societal**  **-**18672  (-1307) |
| Nordgren, 2014, Sweden | **Mental Health**  (anxiety) 100 participants 18+years had to fulfill the DSM-IV criteria for any anxiety disorder as a primary diagnosis. | **Behavioural**  Internet CBT with therapist support vs UC | QALY; improvement in CORE-OM scores  (10 weeks) | Societal; CEA & CUA; Primary Care | **CEA** IC -616 CORE-OM +0.34 ICER -1824  **CUA** IC -474 QALY +0.063 ICUR -7523  (USD, 2010-2013) | **Societal**  -7172  (-452) |
| Nordh, 2021, Sweden | **Mental Health**  (Social Anxiety Disorder) 103 children and adolescents (10-17 years of age) with a principal diagnosis of SAD. | **Behavioural**  Internet CBT with therapist support vs internet delivered supportive therapy | QALY; Change in SAD based on CSR  (3 months) | Societal & Health System; CEA & CUA; Allied Health | **Societal**  **CEA** IC -1076.5 CSR 0.62 ICER-17900.7  **CUA** IC -1076.5 QALY +0.011  ICUR NR (-97845)  (EUR, 2018-2019) | **Societal**  -102572  (-1128) |
| Quinnell, 2014, United Kingdom | **Cardiovascular**  (Obstructive sleep apnea-hypopnoea (OSAHS)) 83 Patients aged ≥18 years with mild to moderate OSAHS confirmed by respiratory polysomnography (rPSG) (AHI 5–<30/h) and symptomatic daytime sleepiness (Epworth Sleepiness Scale (ESS) score ≥9) | **Device**  **3 interventions vs control:**  1.Thermoplastic "boil and bite' device-SleepPro 1  2. Patient-moulded dental impression kit-SleepPro 2  3. Bespoke MAD device | QALY  (4 weeks) | Health System; CUA; Hospital as trial centre | **SleepPro 1**  **CUA** IC -3.87 QALY+ 0.00094 ICUR -4093  **SleepPro2**  **CUA** IC -15.08 QALY+ 0.00088  ICUR-17104  **Bespoke MAD device**  **CUA** IC 26.39 QALY +0.00177 ICUR 14876  (GBP, 2011/12) | **Health**    **SleepPro 1 –**5543  (-5.21)    **SleepPro2** –23091  (-20.32) |
| Romero-Sanchiz, 2017, Spain | **Mental Health**  (Depression)  296 adults presenting with depressive symptoms in primary care | **Behavioural**  ICBT with therapist support + usual GP care vs Usual GP care  ICBT without therapist support +usual GP care vs Usual GP care | QALY  (12 months) | Societal; CEA & CUA; Primary Care | **I-CBT with no therapist support**  **CEA** IC –409.22 BDI 4.16 ICER –98.37  **CUA** IC –409.22 QALY 0.079 ICUR –5160    **I-CBT with therapist**  **CEA** IC 40.93 BDI 4.13 ICER 9.91  **CUA** IC 40.93 QALY 0.0824 ICUR 496.72    (EUR, 2014) | **Societal**    **I-CBT with no therapist support**  -6738  (-532.27) |
| Sava, 2009, Romania | **Mental Health**  (Depression) 141 adults that met criteria for MDD according to the (DSM-IV, scored at least 20 on the BDI, and scored at least 14 or higher on the Hamilton Rating Scale for Depression. | **Behavioural**  Beck's CT (individual F2F CBT) vs Fluoxetine (antidepressant)  REBT (also a form of individual F2F CBT) vs Fluoxetine (antidepressant) | QALY; Depression free days (DFD)  (6 months) | Health System & Payer; CEA & CUA; Hospital as trial centre | **Beck’s CT vs Antidepressant**  **CUA** IC -162.1 QALY+0.01660 ICUR -9765  **REBT vs Antidepressant**  CUA IC –148.39 QALY + 0.00744844 ICUR -19922  (USD, 2006) | **Health**    **Beck’s CT vs Antidepressant**  -9924  (-164.74)  **REBT vs Antidepressant**  -20247  (-150.80) |
| Schwieckert, 2006, Germany | **Musculoskeletal**  (Chronic low back pain) 259 Adults with a history of nonspecific LBP of at least 6 months. | **Behavioural**  CBT with therapist + Standard physical rehabilitation vs Standard physical rehabilitation | QALY  (6 months) | Societal; CUA; 2 rehabilitation centres | **CUA** IC -1670.6 QALY +0.002  ICUR -126731  (EUR, 2001) | **Societal**  -1109600  (-2219) |
| Strauss, 2023, United Kingdom | **Mental Health**  (Anxiety and/or Depression) 410 Adults 18+ who met criteria on the Clinical Interview Schedule-Revised (CIS-R) for a primary diagnosis of a depressive episode, mixed anxiety and depression, or non-specified mild neurotic disorder; scored 10 or more points (clinical cutoff) on the Patient Health Questionnaire (PHQ-9) for depression. | **Behavioural**  Mindfulness-Based  Cognitive Therapy Self-help (MBCT-SH) vs Cognitive Behavioural Therapy Self-help (CBT-SH) | QALY  (42 weeks) | Health System; CUA; Allied Health | **CUA** IC -526 QALY +0.01 ICUR Dominant (-52600)  (GBP, 2017-18) | **Health**  -64614  (-646) |
| Tan, 2010, Netherlands | **Musculoskeletal**  Patellofemoral pain syndrome (PFPS) 131 patients between 14- 40 with symptoms of PFPS and no history of previous active treatment with exercises within the last 6 mths but persisted for longer than 2 mths but no longer than 2 yrs. | **Exercise/Physical Therapy**  Supervised Exercise Program vs usual care | QALY  (12 months) | Societal; CUA; Primary Care/Specialist | **CUA** IC -155 QALY +0.0105 ICUR-14738  (EUR, 2007) | **Societal**  -17353  (-182) |
| Thase, 2020, United States | **Mental Health**  (Depression) 154 adult outpatients presenting for treatment of major depressive disorder (SCI for DSM-IV) unmedicated patients scoring ≥14 on the Hamilton Rating Scale for Depression (HAMD) | **Behavioural**  Computer assisted CBT (CCBT) vs standard F2F CBT | QALY  (6 months) | Societal; CUA; Outpatient | **CUA** IC -945 QALY +0.0007 ICUR Dominant (-1350,000)  (USD, 2013) | **Societal**  -1216272  (-851) |
| Van Lieshout, 2023, Canada | **Mental Health**  (Depression) 329 participants were mothers and birthing parents 18+ with Edinburgh Postnatal Depression Scale (EPDS) scores ⩾10, age, and an infant <12 months of age. | **Behavioural**  1 day CBT Workshop + UC vs UC while Waitlist control | QALY  (12 weeks) | Payer; CUA; Community | **CUA** IC -2602.14 QALY +0.01   ICUR NR (-260214)  (CAD, 2020) | **Payer**  -175053  (-1750.53)    *Payer perspective not graphed |
| Vollbehr, 2023, Netherlands | **Mental Health** (Depression) 171 women 18 to 34 yrs and currently in treatment with a primary diagnosis of MDD using DSM-IV (SCID-I) | **Exercise/Physical Therapy**   Mindful yoga + TAU vs TAU | QALY   (15 months) | Societal; CUA Outpatients | CEA IC -1.852 DASS 6.41 ICER -0.28892  CUA IC -1.852 QALY 0.02 ICUR - 92.6  (EUR,2019, discounted 1.5% health outcomes & 4% costs) | **Societal**  -94  (-1.88) |
| Wijnen, 2018, Netherlands | **Mental Health**   (Depression)  329 Adults 18+ with mild to moderate depressive symptoms defined as a score of 14 to 38 on the Inventory of Depressive symptomatology self-report (IDS-SR). | **Behavioural**  Web-based unguided self-help CDMIs (complaint-directed mini-interventions) based on CBT techniques vs waitlist control + usual care | QALY; rate of responders   (at least 50% decrease in IDS-SR scores)  (3 months) | Societal & Health System; CEA & CUA; Primary care | **Societal**  CEA IC -225 % responders 0.07 ICER -3214 Dominant  CUA IC -225 QALY 0.12 ICUR Dominant~ -1875  **Health** CEA IC -78 % responders 0.07 ICER Dominant~ -1114  CUA IC -85 QALY 0.12 ICUR Dominant~-708   (EUR, 2016) | **Societal**  -2039  (-245)    **Health**  -770  (-92) |
| Witlox, 2022, Netherlands | **Mental Health**  (Anxiety) 314 adults aged 55–75 years, presence of mild to moderately severe anxiety symptoms as measured with the Generalized Anxiety Disorder-7 (GAD-7; scores between 5 and 15) | **Behavioural**  Blended Acceptance and Commitment Therapy vs Brief individual CBT | QALY; % responders to show improvement in anxiety  (12 months) | Societal; CEA & CUA; Primary Care | **CEA** IC -466 % responders -0.06 ICER 7767  **CUA** IC -466 QALY +0.007 ICUR NR-Dominant -66571  (EUR, 2019) | **Societal**  -67720  (-474) |
| Xin, 2020 | **Diabetes**  (Type 2 diabetes) 298 Adults 20 to 65 years with type 2 diabetes diagnosed within previous 6 years | **Nutrition**  Counterweight Plus weight management programme + TAU vs control +TAU | QALY; % in remission   (2 years for CEA & lifetime for CUA) | Health System; CEA & CUA; Primary care | CEA IC 616 % in remission 0.323 ICER 1907  Lifetime CUA IC -1337 QALY 0.059 ICUR -22661  (GBP, 2018) | **Health**    -27362  (-1614) |
| Yu, 2004, USA | **Cardiovascular** (CAD) 269 patients with recent acute myocardial infarction or had elective percutaneous coronary intervention | **Exercise/Physical Therapy** CRPP (exercise +education) vs control (conventional without exercise) | QALY  (2 years) | Health System; CUA; Outpatients | CUA IC -416 QALY 0.64 ICUR –650   (USD, 2001*) | **Health**  -747  (-478) |
| Zhou, 2025, China | **Mental Health**  (Depression) 244 adults with Major depressive disorder (MDD) | **Behavioural**  Unguided internet-based CBT (ICBT) + usual care vs WLC (usual care) | QALY (1 year) | Health & Societal; CUA, Outpatients | **Societal**  **CUA** IC -368.02 QALY 0.0278 ICUR  -13235.44  **Health**  **CUA** IC 9.33 QALY 0.0228 ICUR 409.25  (US, 2022) | **Societal**  -9536.86  (-265.18) |

^a^Intervention was listed only if there was more than one comparison.

^b^based on costs converted to GBP 2023.

*Price year estimated as it was not reported. Estimates are 3 years prior to the year the study was published.

Abbreviations:   CEA= Cost effectiveness analysis, ADAS-cog-plus, CGI-1=, HOOS/KOOS= , 6MWD= ,  CAD= Coronary Artery Disease, CUA= Cost Utility analysis, DSM-IV= ICER = Incremental cost-effectiveness ratio, BDI= Beck depression inventory, ISI= insomnia severity index, ICUR=Incremental cost utility ratio,   CBT= cognitive behavioural therapy, HAMD= HV=Health visitor, PC=patient centred GP= General practitioner, NR=Not reported, CSR= CORE-OM=Clinical Outcomes in Routine Evaluation-Outcome Measure; CSR=Clinician-rated symptom severity, HAI= Health anxiety index, SMFQ= CES-D= Center for Epidemiological studies Depression Scale, GAD-7=Generalized Anxiety Disorder-7, RCI=Reliable change index,  OA= Osteoarthritis, QALY= Quality-adjusted life year, QBDS=Quebec back pain disability scale, SPS= SBP= systolic blood pressure, SCID-1= SF-6D= TAU= treatment as usual, UC=usual care, WOMAC=

**Supplementary Table 8**. Characteristics of studies and CUA interventions that cost more but are more effective (n=74 interventions)

| **Study, Year, Country, Design** | **Category (condition); Population** | **Intervention type; Intervention vs Comparator** | **Outcomes**  **(Time horizon)** | **Perspective, Analysis, Setting** | **Results (Incremental costs (IC); incremental QALY gained/health outcome; ICER/ICUR) (currency, year)** | **Graph details; Perspective, Intervention^a^**  **ICUR^b^ (costs in GBP 2023)** |
| --- | --- | --- | --- | --- | --- | --- |
| Adams, 2021, UK | **Musculoskeletal**  (Thumb base OA); 349 adults >30 years with BTOA | **Device**  Splint vs self- management (SM)  Placebo splint vs self-management (SM) | QALY (12 weeks) | Health System; CUA; Outpatient care | **Splint vs SM**:  IC: 152 QALY 0.00O418 ICUR: 363,640  **Placebo Splint vs SM**:  IC: 98; QALY: -0.000347; ICUR -282051  (GBP 2017/18) | **Health**  **Splint vs SM**  446699  (187) |
| Alfonso-Rosa, 2015, Spain | **Diabetes**  (Type 2 diabetes); 50 adults with type 2 diabetes and attended a primary care clinic | **Exercise/Physical Therapy**  WBV-based exercise therapy + TAU vs TAU | QALY   (3 months) | Health System; CUA; Primary care | CUA; IC 272 QALY +0.075 ICUR 3626.661  (EUR, 2012) | **Health**  4725  (354) |
| Ali, 2024, USA | **Mental health** (Depression) 175 Adults diagnosed with depression in their lifetime with PHW-9 score 10 or above | **Behavioural**  Computer-assisted cognitive behavioural therapy (CCBT) vs TAU | QALY (6 months) | Health System; CUA; Community | CUA; IC 715 QALY 0.0209 ICUR 37295  (US, 2021) | **Health**  28755.65  (551.29) |
| Ambrens, 2022, Australia | **Musculoskeletal** (Falls); 509 adults 70+ years | **Exercise/Physical Therapy**  E health exercise program (Standing Tall) + UC vs UC | QALY; Falls prevented   (2 years) | Health System; CEA & CUA;  Community | **CEA**: IC: 1492; Falls prev:+ 0.31; ICER: 4785  **CUA:** IC: 1492; QALY +0.0257: ICUR: 58,039  (AUD 2020, 5% discounted in 2^nd^ year) | **Health**  32301  (830) |
| Axelsson, 2018, Sweden | **Mental health** (Health Anxiety); 132 adults 18+ years with severe health anxiety | **Behavioural**  3 treatments vs waitlist control:  Bibliotherapy (self-guided CBT with booklet)  Internet CBT (with therapist guided support); Internet CBT (without therapist guided support) | QALY; change in health anxiety-(HAI) % in remission)  (12 weeks) | Societal & Health system; CEA & CUA; Hospital was trial centre | **Societal**  **Bibliotherapy CBT vs control**  **CUA**; IC 128 QALY 0.0053 ICUR 24 245  **Guided I-CBT vs control**  **CUA**; IC 208 QALY 0.0020 ICUR 103 048  **Unguided I-CBT vs control**  **CUA**; IC -57 QALY 0.0034 ICUR -16 951  **Health**  **Bibliotherapy CBT vs control**  **CUA** IC 54 QALY 0.0053 ICUR 10189  **Guided I-CBT vs control**  **CUA** IC 320 QALY 0.0020 ICUR 160000  **Unguided I-CBT vs control**  **CUA** IC 34 QALY 0.0034 ICUR 10000  (GBP, 2014) | **Societal**    **Bibliotherapy CBT vs control**  30985  (164)  **Guided I-CBT vs control**  133430  (267)    **Health**    **Bibliotherapy CBT vs control**  13072  (69)  **Guided I-CBT vs control**  160000  (411)  **Unguided I-CBT vs control**  12829  (44) |
| Bailly, 2018, France | **Cardiovascular** (cardiovascular disease), 50 sedentary adults 18+ with cardiovascular disease | **Exercise/Physical Therapy**  Progressively autonomous physical activity vs standard supervised physical activity) | QALY   (12 months) | Health system; CUA; Community | **CUA**; IC:145; QALY +0.054 QALY; ICUR 2685 (adjusted for baseline QALY)  (EUR 2013-14) | **Health**  2848  (154) |
| Baka, 2021, Netherlands | **Mental health**  (Insomnia) 134 adults 18+ who met the DSM-5 criteria for insomnia disorder | **Behavioural**  guided internet -delivered CBT vs TAU | QALY; change in ISI (insomnia severity index) (26 weeks) | Societal & Health system (Sensitivity analysis); CEA & CUA; Primary care | **Societal**  CEA IC-318 ISI 3.91 ICER -81  CUA IC -318 QALY 0.0113344739 ICUR -28056  **Health**  CEA IC 217 ISI 3.91 ICER 55  CUA IC 217 QALY 0.01 ICUR 20257   (EUR, 2018) | **Health**  20066  (227) |
| Barton, 2009, UK | **Musculoskeletal**  (Knee pain) 389 adults aged 45+ with self- reported knee pain | **Exercise/Physical Therapy** Dietary vs standard care (leaflet); strengthening exercise vs standard care (leaflet); combo of diet + strengthening exercises vs standard care | QALY   (24 months) | Health service; CUA; Primary care | **Strengthening exercise vs standard**  CUA IC 245.73 QALY +0.005 ICUR extended dominance (calculated as 49146)  (GBP 206-07, 2nd year discounted at 3.5%) | **Health**    **Strengthening exercise vs standard**  73900  (369.50) |
| Bennell, 2016, Australia | **Musculoskeletal** (Knee OA); 201 Adult patients with knee OA | **Exercise/Physical Therapy** & **Behavioural**  Supervised Exercise Therapy with pain coping skills training vs just exercise therapy  Supervised Exercise Therapy with pain coping skills training vs just pain coping skills training | QALY (52 weeks) | Societal; CUA; Community | **Supervised Exercise Therapy with pain coping skills training vs just exercise therapy**  **CUA**; IC 626; QALY; 0.03; ICUR NR (22,167)    Supervised Exercise Therapy with pain coping skills training vs just pain coping skills training  **CUA**; IC 335; QALY; 0.03; ICUR NR (11,167)  (AUD, 2010) | **Societal**    **Supervised Exercise Therapy with pain coping skills training vs just pain coping skills training**  7257  (217.71) |
| Biesheuvel-Leliefeld, 2018, Netherlands | **Mental Health** (depression in remission); 248 Adults 18+ with a history of major depressive disorder | **Behavioural**  Preventative Cognitive Therapy (supported self- help) + usual GP care vs usual GP care | QALY; relapse rate of depression (12 months) | Societal & Health system; CEA & CUA; Primary care | **Societal**  **CEA** IC 2114 relapse rate +0.15 ICER  13515  **CUA** IC  2114 QALY +0.03 ICUR 63051  **Health**  **CEA** IC 1107 relapse rate +0.15 ICER 7079  **CUA** IC 1107 QALY +0.03 ICUR 33025  (EUR, 2013) | **Societal**  77750  (2332.51)    **Health**  40714  (1221) |
| Bonin, 2014, United Kingdom | **Mental Health** (Insomnia) 111 Adults 18+ who self-referred | **Behavioural**  CBT workshop vs waitlist control | QALY; severity of insomnia-ISI  (3 months) | Societal; CEA & CUA; Community | **CEA** IC 319 ISI -3.9; ICER **NR**  **CUA** IC 319 QALY +0.004; ICUR **NR**  (GBP 2008/09) | **Societal**  113418  (453.67) |
| Bradshaw, 2024, United Kingdom | **Children** (Eczema)  1208 Children up to 21 days old with relative with diagnosis of eczema | **Other**  Applying daily emollients in the first year to prevent eczema in high-risk children + advice vs advice only (control) | QALY  (24 months) | Health; CEA & CUA; Primary care | **CUA** IC 82.28 QALY 0.0010 ICUR 82580  (GBP, 2019-2020) | **Health**  92176.25  (91.84) |
| Buntrock, 2017, Germany | **Mental Health**   (Depression) 406 Adults 18+ with subthreshold depression | **Behavioural**  Internet guided self -help with support (internet CBT + problem solving therapy) called “GET.ON Mood Enhancer” + usual care vs enhanced Usual GP care | QALY; Depression free days-DFD  (12 months) | Societal & Health system; CEA & CUA; Community | **Societal;**  **CEU** IC 134 DFD +0.12 ICER 1117  **CUA** IC 134 QALY +0.01 ICUR 13400  **Health;**  **CUA** IC 135 QALY +0.01 ICUR 13500  (EUR, 2013) | **Societal**  15442  (154.42)  **Health**  15557  (155.57) |
| Buntrock, 2021, Germany | **Mental Health**  (Insomnia); 128 currently employed schoolteachers aged 18+ with clinically significant insomnia symptoms (ISI>14) and elevated work- related rumination (Irritation scale, subscale "Cognitive Irritation">14) | **Behavioural**  Internet CBT with therapist guided support (GET.ON Recovery) + usual GP care vs Usual GP care waitlist control | QALY; insomnia free-ISI  (6 months) | Societal & Health system; CEA & CUA; Community | **Societal**  **CEU** IC  -1121 ISI +0.30 ICER Dominant  **CUA** IC -1121 QALY +0.02 ICUR Dominant  **Health**  **CEA** IC 225.7 ISI +0.30 ICER 650  **CUA** IC 225.7 QALY +0.02 ICUR 11 285  (EUR, 2013) | **Health**  13004.5  (260.09) |
| Chalder, 2012, UK | **Mental Health** (Depression) 361 adults aged 18 to 69 years with ICD-10 diagnosis of depression and scoring >=14 on BDI | **Exercise/Physical Therapy**  Physical exercise + TAU vs TAU | QALY  (12 months) | Health service; CUA; Primary care | CUA IC 296 QALY 0.0142 ICUR 20834  (GBP, 2009, discounted at 12 months 3.5%) | **Health**  29074  (413) |
| Chapdelaine, 2023, Canada | **Mental Health**   (Anxiety disorder) 231 adults 18 to 65 meeting DSM-5 for panic disorder, agoraphobia, social anxiety disorder or GAD | **Behavioural**  transdiagnostic CBT group therapy + TAU vs TAU | QALY   (up to 8 months) | Societal & Health System; CEA & CUA; Primary care | **Health system:**  CEA IC+626 AFD +38 ICER 15.88/AFD CUA IC +626 QALY +0.026 ICUR 30290.6  **Societal:**  CEA IC 691 ICER 21.64/AFD  CUA IC 691 ICUR 40309.81  (CAD, 2020) | **Health**  16197  (421)  **Societal**  17879  (465) |
| Chew, 2022, USA | **Cardiovascular**  (Heart failure) 349 adults 60+ who had been admitted for acute decompensated heart failure | **Exercise/Physical Therapy**  Rehabilitation group vs control | QALY  (6 months) | Health System; CUA; outpatients | CUA IC 2976 QALY 0.0100 ICUR 292521  (USD, 2019, discounted 3% per year) | **Health**  242475  (2429) |
| Chuang, 2012, United Kingdom | **Musculoskeletal**  (Chronic low back pain) 313 patients aged 18 to 65 years who had consulted their general practitioner with low back pain in the last 18 months. | **Exercise/Physical Therapy**  Yoga vs usual care | QALY (12 months) | Societal & Health System; CUA; Primary care | **Societal**  **CUA** IC -213.9 QALY +0.037 ICUR Dominant  **Health**  **CUA** IC +506.8 QALY +0.037 ICUR 13606  (GBP, 2008/09) | **Health**  19480  (721) |
| Coyle, 2012, Canada | **Diabetes**  (Type 2 Diabetes) 251 subjects aged 39 to 70 with a diagnosis of type 2 diabetes | **Exercise/Physical Therapy**  **3 interventions (DARE clinical trial);**  Resistance exercise vs wait list control  Aerobic exercise vs waitlist control  combo of resistance and aerobic exercise vs waitlist control | QALY; life years   (12 months with 40- year simulation data and costs **based on lifetime costs**) | Societal; CUA; Community | **Resistance exercise**  **CUA**; IC 7244 QALY +0.03 ICUR 206 985  **Aerobic exercise**  **CUA**; IC 8175 QALY +0.07 ICUR 116793  **combo of resistance and aerobic exercise**  **CUA**; IC 8975 QALY +0.24 ICUR 37782  (CAD, 2008, discounted at 5% per annum) | Based on lifetime costs so not graphed |
| Dams, 2019, Germany | **Mental Health**  (Social anxiety disorder)  103 Patients 14-20 years old with a primary diagnosis of SAD | **Behavioural**  CBT vs wait list control  Psychodynamic therapy (PDT) vs waitlist control | QALY (6 months) | Payer; CUA; Hospitals are the trial centres (multi-centre) | **CBT vs wait list control**  **CUA;** IC 320 QALY +0.017 ICUR 18824  (EUR, 2014) | **Payer**  **CBT vs wait list control**  21294  (362)  *Payer perspective not graphed |
| Davis, 2022, Canada | **Musculoskeletal**  (Falls) 344 adults aged 70 or older who sustained a fall in the past 12 month. | **Exercise/Physical Therapy**  Otago Exercise Program + UC vs UC | QALY; falls prevented   (12 months) | Health system; CEA & CUA; Community; (**stratified by gender**) | **Women**  **CEA** IC 26 fall avoided -0.6 ICER -42  **CUA** IC 26 QALY +0.001 ICUR 28582  **Men**  **CEA** IC -1104 fall prevented -1.2 ICER 920 (dominates)  **CUA** IC -1104 QALY +0.0046 ICUR 238913 (CAD, 2019) | **Health**    **Women**  17600  (17.60) |
| Dear, 2015, Australia | **Mental Health**  (Anxiety) 72 participants were at least 60 years of age and a self- reporting of difficulties with anxiety | **Behavioural**  Internet CBT with therapist support vs waitlist control | QALY  (8 weeks) | Health system; CUA; Community | **CUA** IC 92.2 QALY +0.010 ICUR 8806  (AUD, 2013) | **Health**  5680  (57) |
| de Jonge, 2024, Netherlands | **Mental Health** (Depression) 214 adults with a history of 2 or more major depressive episodes and currently in remission for at least 2 months by DSM-IV using SCID-1 | **Behavioural**  preventive cognitive therapy (adapted form of CBT) vs TAU | QALY; DFDs  (15 months) | Societal & Health System; CUA & CEA; Primary care | **Societal**  **CEA** IC 858 DFDs 41 ICER 20 **CUA** IC 858 QALY 0.0061 ICUR 141451  **Health System**  **CEA** IC 1973 DFD 41 ICER 47 **CUA** IC 1973 QALY 0.0061 ICUR 378922  (EUR, 2014) | **Societal**  155679  (944)  **Health**  357988  (2171) |
| De Vries, 2019,  Netherlands | **Cardiovascular**  (moderate obstructive sleep apnoea) 85 patients 18+ with moderate OSA (AHI 15 to 30 events/h**)** | **Device**  MAD (mandibular advancement device) vs CPAP (continuous positive airway pressure) | QALY; reduction in apnoea-hypopnea index -AHI  (12 months) | Societal; CEA & CUA; Allied Health sleep centres | **CEA** IC 2156.5 AHI -7.07 ICER -305  **CUA** IC  2156 QALY + 0.064 ICUR 33701  (EUR, 2015) | **Societal**  36791  (2355) |
| Egger, 2015, Germany | **Mental Health**  (Social Anxiety Disorder) 495 Adults aged 18 to 70 years with social anxiety disorder according to the German version of the Structured Clinical Interview (SCID) and Liebowitz social anxiety score (LSAS) score >30 | **Behavioural**  **3 arm;**  CBT with therapist vs waitlist control  Psychodynamic therapy (PDT) vs waitlist control | QALY; rate of remission- Liebowitz social anxiety scale (LSAS <=30)  (6 months) | Societal; CEA & CUA; Community/Primary Care/Outpatient clinic | **CBT with therapist vs waitlist control**    **CEA** IC 1625 LSAS remitted +0.27 ICER 5788  **CUA** IC 1625 QALY +0.015 ICUR 107645  (EUR, 2008) | **Societal**    **CBT with therapist vs waitlist control**  133842  (2008) |
| Eriksson, 2010, Sweden | **Cardiovascular**  (High risk of CVD) 151 adults aged 18 to 65 years at moderate to high risk of cardiovascular disease (with hypertension, dyslipidaemia, type 2 DM, obesity, or any combination) | **Exercise/Physical Therapy**    Lifestyle-modification program +UC vs UC | QALY (measured via EQ-5D, EQ-VAS and SF-6D);  (3 years) | Societal; CUA; Primary Care | **EQ-5D;**  **CUA** IC +336.7 QALY+0.075 ICER 4492.0  **SF-6D;**  **CUA** IC +336.7 QALY+0.070 ICUR 4812.9    (USD, 2009, discounted 3% per year) | **Societal**  4331  (325) |
| Gillespie, 2013, Ireland | **Cardiovascular** (COPD) 350 patients with a diagnosis of COPD as defined by the GOLD guidelines | **Exercise/Physical Therapy** Exercise + Education vs TAU | QALY; CRQ  (22 weeks) | Health System; CEU & CUA; Primary Care | **CEA** IC 944 CRQ 1.11 ICER 850  **CUA** IC 944 QALY 0.002 ICUR 472000   (EUR, 2009) | **Health**  526730  (1054) |
| Groessl, 2020, Netherlands | **Musculoskeletal**  (chronic low back pain) 150 Military veterans with chronic low back pain (defined as lasting 12+weeks). | **Exercise/Physical Therapy**  Yoga vs Waitlist control | QALY  (12 months) | Societal & Health system; CUA; Hospital is the trial centre | **Societal**  **CUA** IC -1899 QALY +0.043 ICUR Dominant (-44163)  **Health**  **CUA** IC 193 QALY 0.043 ICUR 4488  (USD, 2017) | **Health**  3818  (164) |
| Gueugnon, 2021, France | **Musculoskeletal**  (OA Knee) 90 Adult (aged >40 years) patients with symptomatic medial knee OA (ERGONOMIE study) | **Device**  Knee Brace, ODRA + UC vs UC | QALY  (12 months) | Societal; CUA; Primary Care/ Hospital | **CUA** IC 1335 QALY +0.08 ICUR 16683  (EUR, 2015/2016) | **Societal**  17392  (1391) |
| Gusi, 2008, Spain | **Mental Health**  (moderate depression, or overweight or obese) 106 women were aged 60 years and older, suffered from either moderate depression (6-9 on GDS) or were overweight (BMI 25 to 39.9), and were capable of walking for more than 25 minutes. | **Exercise/Physical Therapy**  Supervised walking programme vs usual GP care | QALY  (6 months) | Health system; CUA; primary Care | **CUA** IC 41 QALY +0.132 ICUR 311  (EUR, 2005) | **Health**  457  (60) |
| Hakkaart-Van Roijen, 2006, Netherlands | **Mental Health**  (depression and/or anxiety) 702 Adults aged 18 to 65 years, eligible for out-patient mental healthcare, not treated by the same MHC in the past year and were diagnosed with major depressive disorder (single or recurrent) dysthymic disorder; panic disorder (with or without agoraphobia) social phobia, or generalised anxiety disorder | **Behavioural**  Brief therapy (stepped-care approach) vs Usual GP care  CBT with therapist vs Usual GP care  Brief therapy (stepped-care approach) vs CBT with therapist | QALY  (18 months) | Societal; CUA; Outpatient | **CBT vs UC**  **CUA** IC 237 QALY +0.027 ICER 8778    **CBT vs Brief Therapy**  **CUA** IC -468 QALY -0.003 ICER 156000  (EUR, 2002) | **Societal**    **CBT vs UC**  11392  (308) |
| Hedman, 2016, Sweden | **Mental Health**  (Severe Health Anxiety) 151 adult patients 18+ with a principal diagnosis of severe health anxiety (hypchondriasis) according to DSM-IV | **Behavioural**  Internet CBT with therapist support vs internet behavioural stress management | QALY; % with improvement in Health anxiety-HA  (3 months) | Societal; CEA & CUA; Hospital as Trial centre/ Primary care | **CEU** IC 310 HA +0.14   ICER 2214  **CUA** IC 310 QALY +0.031 ICUR 10,000    (USD, 2013) | **Societal**  9009  (279) |
| Hochheim, 2021, Germany | **Musculoskeletal**  (low back pain)  223 adults with back pain based on at least two ICD-10 codes M40 to M54 (dorsopathies) | **Exercise/Physical Therapy**   Multimodal therapy (specific exercise training and behavioural support) vs control (TAU) | QALY  (24 months) | Payer; CUA; Primary Care | CUA IC 719 QALY 0.087 ICUR 8296  (EUR, 2020) | **Payer**  8440  (732)     *Payer perspective not graphed |
| Hollinghurst, 2014, United Kingdom | **Mental Health**  (Depression) 469 Adults 18 to 75 years and have depression, as indicated by a Beck depression (BDI-II) Inventory score of 14 or above and and ICD-10 diagnosis of depression using the Revised Clinical Interview Schedule (CIS-R) | **Behavioural**  CBT with therapist + Usual GP care vs Usual GP care | QALY  (12 months) | Health System & Payer; CUA; Primary Care | **CUA** IC 850 QALY +0.057 ICUR 14911  (GBP, 2010) | **Health**  20516  (1169) |
| Hollinghurst, 2010, United Kingdom | **Mental Health**  (Depression) 137 adults 18 and 75 with a new episode of depression. This was defined as a score of 14 or more on the Beck Depression Inventory (BDI) 12 and an ICD–10 diagnosis of depression using the Revised Clinical Interview Schedule (CIS–R). | **Behavioural**  Internet CBT with own therapist + Usual GP care vs Usual GP care | QALY; % those recovered (BDI <10)  (12 months) | Health System; CEA & CUA; Primary Care | **CEA** IC 469 % recovery +0.133 ICER 3528  **CUA** IC 469 QALY +0.027 ICUR 17173  (GBP, 2007) | **Health**  25525  (689) |
| Hornby, 2022, United States | **Cardiovascular**  (post unilateral stroke) 44 patients aged 18-75 years with a history of unilateral stroke in the last 1-6 months, ability to walk with moderate assistance or less (ie, perform at least 50% of work to ambulate), including the use of braces and devices as necessary, but at self-selected speeds (SSSs) <0.9 m/s; | **Exercise/Physical Therapy**  HIT (high intensity exercise) vs Conventional physical therapy | QALY; Change in Self-selected speed-SSS  (6 months) | Health System; CEA & CUA; Outpatient | **CEA** IC 309 SSS +0.20 ICER 155 per 0.10 m/s  **CUA** IC 309 QALY +0.05 ICUR 6180  (USD, 2016) | **Health**  5358  (268) |
| Johnson, 2007, United Kingdom | **Musculoskeletal**  (Chronic Back Pain) 227 Patients 18 to 65 years of age, consulting with LBP, were recruited; those still reporting LBPat  3 months with pain score >=20mm or more (100-mm VAS) and RMDQ disability score of >=5 | **Combo of both Exercise/Physical Therapy/Behavioural**  Group Exercise + CBT group F2F vs usual GP care | QALY  (15 months) | Health System; CUA; Primary Care | **CUA** IC 27 QALY +0.0054 ICUR 5000  (GBP, 2003/04) | **Health**  8189  (44) |
| Kahlke, 2023, Germany | **Mental Health**   (Social anxiety disorder) 200 adults 18+ and scored >21 on the Social Phobia Scale (SPS and/or >32 on Social Interaction Anxiety Scale (SIAS) and met criteria of SAD based on SCID-I (DSM-IV) | **Behavioural**  StudiCare (text-based CBT) vs waitlist control | QALY; Symptom free status SPS & SIAS   (6 months) | Societal & Health System; CEA & CUA; Primary care | **Societal**  CEA IC -321 SPS 0.26 ICER dominant CUA IC -319 QALY 0.046 ICUR Dominant~-6978.26  **Health**  CEA IC 81 SPS 0.255 ICER 348 CUA IC 81 QALY 0.046 ICER 1945  (EUR, 2017) | **Health**  1901  (87.46) |
| Killingmo, 2023, Norway | **Musculoskeletal**  (Knee OA)  161 Adults with knee OA | **Exercise/Physical Therapy**  Strength exercise (SE) vs UC  Aerobic exercise (AE) vs UC | QALY (1 year) | Health & Societal; CUA, Community | **Societal**  **SE to UC;** IC -9445 QALY 0.05 ICUR  -188 900  **AE vs UC;** IC -9297 QALY 0.04 ICUR  -232425  **Health**  **SE to UC ;** IC -805 QALY 0.05 ICU  -16100  **AE vs UC ;** IC 349 QALY 0.04 ICUR 8725  (EUR, 2020) | **Health**  AE vs UC  8707.77  (348.31) |
| Klein, 2018, Netherlands | **Mental Health**  (Depression) 264 Adults aged 18 to 65 years with a history of at least 2 major depressive episodes (MDEs) according to (DSM-IV) criteria assessed with the Structured Clinical Interview for DSM-IV Disorders (SCID-I) of which the latest MDE occurred within the last 2 years and currently remitted for at least 2 months according to SCID-I and a score of ≤10 on the Hamilton Rating Scale for Depression (HRSD) | **Behavioural**  Internet CBT with therapist support + Usual GP care vs Usual GP care | QALY; Depression free days (DFD)  (24 months) | Societal; CEA & CUA; Primary Care | **CEA** IC 1008 DFD +5.6 ICER 179  **CUA** IC 1008 QALY +0.01 ICUR 230816  (EUR, 2014, costs discounted at 4%) | **Societal**  110939  (1109) |
| Kolovos, 2016, Netherlands | **Mental Health** (Depression) 269 adults 18+ and on a waiting list for face-to-face treatment and met criteria for major depressive disorder (MDD) according to DSM-IV as measured with CIDI | **Behavioural**  Internet-based therapy (based on problem-solving therapy) + waiting for face-to-face vs enhanced waitlist control (waiting for face-to face+ unguided self-help book) | QALY; CES-D (improvement in CES-D & response to treatment)  (12 months) | Societal; CEA & CUA; Primary care | CEA (improvement in symptoms) IC 1579 CES-D 0.49 ICER 3222 CEA (response to treatment) IC 1579 CES-D 0.01 ICER 157900  CUA IC 1579 QALY 0.01 ICUR   157900  (EUR, 2013) | **Societal**  174221  (1742) |
| Koppenaal, 2023, Netherlands | **Musculoskeletal**  (low back pain) 208 adults 18+ and received physiotherapy treatment for nonspecific low back pain | **Exercise/Physical Therapy**  E-Exercise (smartphone app integrated into face-to-face physiotherapy treatment) vs face-to-face physiotherapy | QALY; improvement in ODI  (12 months) | Societal & Health System; CUA & CEA; Primary care | **Societal**  CEA IC 1004 ODI -1.63 ICER -614   CUA IC 994 QALY 0.02 ICUR 49159  **Health**  CEA IC 47 ODI -1.69 ICER -28 CUA IC 47 QALY 0.02 ICUR 2239  (EUR, 2020) | **Societal**  49602  (992)    **Health**  2346  (46.91) |
| Kraepelien,2018, Sweden | **Mental Health** (Depression) 945 Adults 18-67 and present depressive symptoms defined as scoring >=10 on the PHQ-9. | **1 in Behavioural & 1 in Exercise/Physical Therapy**  Internet-based CBT vs TAU; Physical exercise vs TAU | QALY  (12 months) | Societal & Health; CUA; Primary care | **Health**  **ICBT vs Tau**  ICUR IC 343.08 QALY +0.0338 ICUR 10166  **PE vs TAU**   CUA IC 920.14 QALY+ 0.0334 ICUR 27560  **Societal**  **ICBT vs TAU**   ICUR IC 1061.88   QALY 0.0338 ICUR 31417  **PE vs TAU**   IC 1266.6 QALY 0.0334 ICUR 37922  (EUR, 2012) | **Health**  **ICBT vs TAU**  11342  (383)  **PE vs TAU**  30784  (1028)  **Societal**  I**CBT vs TAU**  35106  (1187)  **PE vs TAU**  42389  (1416) |
| Kyle, 2024, United Kingdom | **Mental Health (Insomnia)**  642 Adults with insomnia disorder | **Behavioural**  Sleep restriction CBT (SRT) vs sleep hygiene (control) | QALY  (12 months) | **Health**; CUA; Primary Care | **Health**  IC 43.59 QALY 0.021 ICUR 2076  (GBP, 2018-19) | **Health**  2454.72  (51.54) |
| Lamb, 2010, United Kingdom | **Musculoskeletal**  (Chronic low back pain) 528 Adults 18+ with subacute and chronic LBP who were experiencing symptoms that were at least moderately troublesome>6 weeks (BeST trial) | **Behavioural**  Group F2F CBT + Usual GP care vs Usual GP care | QALY  (12 months) | Health System; CUA; Primary Care | **CUA** IC 314.37 QALY +0.099 ICUR 3093  (GBP, 2008) | **Health**  4516  (447.09) |
| Li, 2022, China | **Cardiovascular**  (High risk of stroke) 20955 adults with high risk of stroke based on either a history of stroke or age ≥60 years with uncontrolled high blood pressure. | **Nutrition**  Salt substitute vs Control | QALY; Strokes averted  (5 years) | Joint perspective of Health System & consumers; CEA & CUA; Community | **Using Median price of salt substitute**  CEA IC 106 strokes averted+ 0.14  ICER 5512  CUA IC 106 QALY +0.054 ICUR 1980    (CNY, 2014, discounted at 5% per annum) | **Health**  452  (24.39) |
| Lynch, 2005, United States | **Mental Health**  (Depression) 94 Teens 13 to 18 years old at risk for depression; based on identifying parents of teenagers who had had at least 2 dispensations of an antidepressant medication and/or mental health visits within the past year. Teens reported a previous depression episode or subdiagnostic levels of depressive symptoms that were insufficient to meet full criteria for a DSM-III-R affective diagnosis (Center for Epidemiologic Studies Depression Scale score,>=24). | **Behavioural**  Group F2F CBT vs Usual GP care | QALY; Depression free days (DFD)  (12 months) | Societal; CEA & CUA; Primary Care | **CEA** IC 610 DFD +53 ICER 10  **CUA** IC 610 QALY +0.059 ICUR 9275  (USD, 2000) | **Societal**  12147  (717) |
| Maru, 2019, Australia | **Cardiovascular**  (heart failure) 278 Adults with symptomatic heart failure within 6 weeks of hospital discharge (the EJECTION-HF study) | **Exercise/Physical Therapy**  Supervised center-based exercise training + disease management program (UC) vs disease management program (UC) | QALY  (12 months) | Health System; CUA; Outpatient | **CUA** IC 2405 QALY +0.02 ICUR 128889  (AUD, 2015) | **Health**  74397  (1488) |
| Matchar, 2019, Singapore | **Musculoskeletal**  (Falls) 323 Adults 65+ who present to the ED for a fall or fall related injury, and subsequently discharged to their home in the community (the SAFE study) | **Exercise/Physical Therapy**  Multifactorial fall prevention program vs Usual GP care | QALY  (9 months) | Health System; CUA; Outpatient | **CUA** IC 362 QALY +0.003 ICUR 120667  (SGD, 2015) | **Health**  127340  (382) |
| McCrone, 2012, UK | **Musculoskeletal**   (Chronic fatigue syndrome)  640 Adults 18 + attending outpatient CFS clinics | **Exercise/Physical Therapy** Adaptive pacing therapy (APT), Cognitive behaviour therapy (CBT), or Graded exercise therapy (GET) vs Specialist medical care (SMC) | QALY; fatigue ~also had ICER based on disability  (12 months) | Societal & Health care System; CEA & CUA; Primary care | **GET vs SMC**  **Health**  **CEA** IC 837 fatigue 14.0 ICER 5987  **CUA** IC 810 QALY 0.0343 ICUR 23615  **Societal**  **CEA** IC -400 fatigue 14.0 ICER dominant  **CUA** IC -472 QALY 0.0343 ICUR 13761 dominant  (GBP, 2009/2010) | **Health**  32955  (1130) |
| Meuldijk, 2015, Netherlands | **Mental Health**  (Depression and/or anxiety) 182 patients aged 18 to 65 years with a mild to moderate anxiety and/or depressive disorder," | **Behavioural**    Concise care (SSRI or CBT up to only 7 weeks) vs Standard care | QALY  (12 months) | Societal; CUA; Outpatient | **CUA** IC 4795 QALY +0.005 ICUR 959000  (EUR, 2013) | **Societal**  1058120  (5291) |
| Miyamoto, 2018, Brazil | **Musculoskeletal**   (low back pain) 296 patients 18 to 80 yrs with non-specific chronic low back pain (NSCLBP) | **Exercise/Physical Therapy**  Pilates -3 different doses (P1= 1/wk P2=2/wk P3=3/wk)  vs control (booklet) | QALY   (12 months) | Societal; CEA & CUA; Primary care/Allied health | **P1 vs booklet** CUA IC -75 QALY 0.01 ICUR -7008  **P2 vs booklet** CUA IC 174 QALY 0.02 ICUR 7053  **P3 vs booklet** CUA IC 230 QALY 0.04 ICUR  5503  (GBP, 2016) | **Societal**    **P2** 10879  (218)  **P3** 7190  (288) |
| Morgan, 2004, United Kingdom | **Mental Health**  (insomnia) 124 Patients aged 31 to 92 years with chronic sleep problems who had been receiving repeat hypnotic drug prescriptions for at least 1 month. All met Diagnostic and Statistical Manual of Mental Disorders (DSM-IV) criteria for insomnia | **Behavioural**  CBT with therapist vs Control | QALY  (6 months) | Health System; CUA; Primary Care | **CUA** IC 129.9 QALY +0.038 ICUR 3418  (GBP, 1999/2000) | **Health**  6052  (230) |
| Mourad, 2022, Sweden | **Mental Health**  (Depression) 144 CVD patients age ≥18 years with no hospitalisations during the past 4 weeks, had been in contact with the medical or cardiac clinics at four hospitals in Southeastern Sweden and had depressive symptoms with a score ≥5 on the Patient Health  Questionnaire-9 | **Behavioural**  Internet CBT guided by nurse’s vs Online discussion forum (ODF) | QALY  (12 months) | Health System; CUA; Primary Care/Hospital | **ICUR** IC 2174.6 QALY + 0.11527   ICUR 18 865  (EUR, rate of May 2021-costs from 2017-2019) | **Health**  18293  (2109) |
| Murphy, 2012, United Kingdom | **Mental Health**  (CHD risk, Depression, Anxiety or stress) 786 sedentary patients 16 +yrs (defined as not moderately active for >=3 times per week), have at least on of; CHD risk factor or Mental health condition (mild anxiety, depression or stress) | **Exercise/Physical Therapy**  NERS (National Exercise Referral Scheme) vs UC | QALY  (12 months) | Health System; CUA; Primary Care | **ICUR** IC 327 QALY + 0.027   ICUR 12111  (GBP, 2010) | **Health**  16663  (450) |
| Nordh, 2021, Sweden | **Mental Health**  (Social Anxiety Disorder) 103 children and adolescents (10-17 years of age) with a principal diagnosis of SAD. | **Behavioural**  Internet CBT with therapist support vs internet delivered supportive therapy | QALY; Change in SAD based on CSR  (3 months) | Societal & Health System; CEA & CUA; Allied Health | **Health**  **CEA** IC 31.48 CSR 0.62 ICER 51  **CUA** IC 31.48 QALY +0.011  ICUR NR 2862  (EUR, 2018-2019) | **Health**  2999  (32.99) |
| Oppong, 2014, UK | **Musculoskeletal**   (Hand OA) 257 Adults aged 50+ | **Exercise/Physical Therapy**  4 treatment groups ~Hand exercises only, joint protection only, joint protection + hand exercises vs leaflet +advice (control) | QALY  (12 months) | Health service; CUA; Primary care | **Hand exercises vs control**  CUA IC 6.05 QALY 0.019 ICUR 318   (GBP, 2010/2011) | **Health**  438  (8.32) |
| Patrick, 2001, USA | **Musculoskeletal** (OA)  249 adults 55 to 75 with a doctor confirmed diagnosis of osteoarthritis | **Exercise/Physical Therapy**  Aquatic classes vs control | QALY based on CHDR ~current Health desirability rating or QWB ~ quality of well-being scale  (lifetime costs) | Societal; CUA; Primary care | IC 11363 QALY (CHDR) 7.03 ICUR with 3% discounting 32643  (USD, 1997, future costs discounted 3%) | **Societal**  1992  (14001)  Costs based on lifetime so not graphed |
| Pinto, 2013, New Zealand | **Musculoskeletal**  (OA Hip or Knee) 206 Adults who met the American College of Rheumatology criteria for hip or knee osteoarthritis | **Exercise/Physical Therapy**  **(3 interventions vs Usual GP care alone)**  Exercise Physiotherapy + Usual GP Care  Manual Physiotherapy + Usual GP Care  Combined Exercise and Manual physiotherapy + Usual GP care | QALY; % of OMERACT-OARSI responders  (12 months) | Societal & Health System; CEA & CUA; Primary Care | **Societal**  **Exercise Physiotherapy**  CEA 551.64 responders +0.10 ICER 7869  CUA 551.64 QALY +0.04 ICUR 23365  **Manual Physiotherapy**  CEA -154.98 responders +0.22 ICER -6905  CUA -154.98 QALY +0.009 ICUR –38072  **Health**  **Exercise Physiotherapy**  **CUA** IC 654.53 QALY 0.04 ICUR 26400  **Manual Physiotherapy**  **CUA** IC 660.55 QALY 0.009 ICUR 37964 | **Societal**    **Exercise Physiotherapy**  9292  (371.66)    **Health**  **Exercise Physiotherapy**   17789  (440.98) |
| Quinnell, 2014, United Kingdom | **Cardiovascular**  (Obstructive sleep apnea-hypopnoea (OSAHS)) 83 Patients aged ≥18 years with mild to moderate OSAHS confirmed by respiratory polysomnography (rPSG) (AHI 5–<30/h) and symptomatic daytime sleepiness (Epworth Sleepiness Scale (ESS) score ≥9) | **Device**  **3 interventions vs control:**  1.Thermoplastic "boil and bite' device-SleepPro 1  2. Patient-moulded dental impression kit-SleepPro 2  3. Bespoke MAD device | QALY  (4 weeks) | Health System; CUA; Hospital as trial centre | **SleepPro 1**  **CUA** IC -3.87 QALY+ 0.00094 ICUR -4093  **SleepPro2**  **CUA** IC -15.08 QALY+ 0.00088  ICUR-17104  **Bespoke MAD device**  **CUA** IC 26.39 QALY +0.00177 ICUR 14876  (GBP, 2011/12) | **Health**    **Bespoke MAD device**    93961  (166) |
| Reed, 2010, United States | **Cardiovascular**  (Heart failure) 2331 outpatients had left ventricular ejection fraction (LVEF) of <=35% and New York Heart Association class II to IV symptoms (medically stable heart failure) | **Exercise/Physical Therapy**  Exercise training (HF-ACTION) + Usual GP care vs Usual GP care | QALY  (2 years) | Societal; CUA; Outpatient | **CUA** IC +1161 QALY +0.03 ICUR NR (38700)  (USD, 2008, discounted 3% per annum>1yr) | **Societal**  37574  (1127) |
| Revicki, 2005, United States | **Mental Health**  (Depression) 267 Women who screened positive for major depression using the Primary Care Evaluation of Mental Disorders. Included participants were found to be low-income minority women. | **Behavioural**  **3 interventions:**  CBT with therapist vs Community referral  Pharmacotherapy vs Community referral | QALY; Depression free days (DFD)  (12 months) | Health System; CEA & CUA; Primary Care | **CBT with therapist vs Community referral: using outpatient costs**  **CEA** IC 636 DFD +25.80 ICER 27.04  **CUA** IC 636 QALY +0.036 ICUR 17624  (USD, 2002) | **Health**  19988  (719.56) |
| Rhon, 2022, USA | **Musculoskeletal** (Knee OA) 156 adults 38 + years and met the criteria for knee OA with radiographic evidence of osteoarthritis | **Exercise/Physical Therapy**  Physical therapy vs glucocorticoid injections | QALY   (12 months) | Health care; CUA; Primary care | CUA IC 2145 QALY 0.076 ICUR 28271  (USD, 2019) | **Health**  23074  (1751) |
| Romero-Sanchiz, 2017, Spain | **Mental Health**  (Depression)  296 adults presenting with depressive symptoms in primary care | **Behavioural**  ICBT with therapist support + UC vs Usual GP care  ICBT without therapist +UC vs Usual GP care | QALY; Severity of Depression (BDI-II)  (12 months) | Societal; CEA & CUA; Primary Care | **I-CBT with therapist**  **CEA** IC 40.93 BDI 4.13 ICER 9.91  **CUA** IC 40.93 QALY 0.0824 ICUR 496.72    **I-CBT with no therapist support**  **CEA** IC –409.22 BDI 4.16 ICER –98.37  **CUA** IC –409.22 QALY 0.079 ICUR –5160    (EUR, 2014) | **Societal**    **I-CBT with therapist**  646  (53.24) |
| Smit, 2013, Netherlands | **Mental Health**  (Smoking Cessation)  414 smokers aged 18+ | **Behavioural**  3 arm intervention; Internet-based multiple computer-tailored program (MT) vs MT + counselling(C) vs Usual care (UC) | QALY; prolonged abstinence (PA) at 12 months  (12 months) | Societal; CEA & CUA; Primary care | **MT vs UC**  **CEA** IC 255 PA 0.05 ICER 5100 **CUA** IC 255 QALY -0.01   ICUR -25500  **MT +C vs UC**  **CEA** IC 806 PA -0.02 ICER Dominated  **CUA** IC 806 QALY 0.02 ICUR 40300 (EUR, 2011) | **Societal**    **MT + C vs UC**  45685  (914) |
| Stanczyk, 2014, Netherlands | **Mental Health**  (Smoking Cessation)  2099 adult smokers 18+ and motivated to quit smoking within 6 months | **Behavioural**  3 arm; video-based vs text-based computer-tailored smoking cessation interventions vs control | QALY; prolonged abstinence (PA) (12 months) | Societal; CEA & CUA; Primary care | **Video vs control**  CEA IC 60 QALY +0.004 ICER 1500  CUA IC 60 QALY 0.001 ICUR 60000  **Text vs Control**  CEA IC 504 PA +0.001 ICER 50 400  CUA IC 504 QALY -0.002 ICUR  -252000  (EUR, 2013) | **Societal**  **video vs control**  66200  (66) |
| Stanmore, 2019, UK | **Musculoskeletal** (Falls) 106  Adults aged 55 + living in 18 assisted living facilities in the UK. | **Exercise/Physical Therapy** Physiotherapy (Exergames) + leaflet (standard care) vs leaflet (standard care) | QALY  (3 months) | Health System; CUA; Primary care | CUA IC 101.84 QALY 0.0067 ICUR 15210  (GBP, 2015-2016) | **Health**  19375  (129.73) |
| Titov, 2015, Australia | **Mental Health**  (Depression) 54 Australian adults over 60 years of age with symptoms of depression | **Behavioural**  Internet CBT with therapist support vs Waitlist control | QALY  (8 weeks) | Health System; CUA; Primary Care | **CUA** IC 52 QALY +0.012 ICUR 4392  AUD, 2013) | **Health**  2669  (32) |
| Turner, 2017, UK | **Mental Health** (Depression) 86 young people aged 14 to 17 yrs attending outpatient services presenting with depression | **Exercise/Physical Therapy**   Preferred intensity exercise + TAU vs TAU | QALY; point change in CDI-2 * negative value for incremental effect is positive (children's Depression Inventory)  (6 months) | Health System; CEA & CUA; Primary care | CEA IC 292 CDI-2 -4.8 ICER 61 CUA IC 286 QALY 0.00187   ICUR 152822   (GBP, 2012/2013) | **Health**  202885  (380) |
| Ulfsdottir, 2023, Sweden | **Cardiovascular** (Intermittent claudication) 99 adults with at least 6 months of mild to severe IC of vascular origin with an ankle-brachial index (ABI) of less than 0.9 and /or postexercise ABI reduction of 30% or more. | **Exercise/Physical Therapy**  3 intervention groups; Supervised exercise (SE)+ WA, Home-based structured exercise (HSEP)+ WA, and walk advice alone (WA) | QALY   (12 months) | Health system; CUA; Primary Care | **HSEP vs WA**  CUA IC 39.57 QALY 0.01 ICUR 3749   (EUR, 2020*) | **Health**  3949  (39.49) |
| Van Asselt, 2011, Netherlands | **Cardiovascular**  (Intermittent Claudication) 252 patients with peripheral arterial disease, stage II according to Fontaine and eligible for conservative treatment, (an ankle-brachial index (ABI) less than 0.9 and an absolute claudication distance (ACD) below 500 m) were enrolled in the EXITPAD study. | **Exercise/Physical Therapy**  Supervised exercise therapy (SET) vs unsupervised walking advice (WA) | QALY; change in walking distance  (12 months) | Societal; CEA & CUA; Outpatient | **CEA** IC 1104 walking distance +200 m ICER 4.08 per metre  **CUA** IC 1104 QALY +0.038 ICUR 28693  (EUR, 2008) | **Societal**  33383  (1269) |
| Williamson, 2023, UK | **Musculoskeletal**   (Low back pain from neurogenic claudication)  435 adults 65+ who reported symptoms consistent with NC | **Exercise/Physical Therapy** BOOST programme vs BPA (best practice advice) | QALY  (12 months) | Societal & Health System; CUA; Primary care/Allied health | **Health**  **CUA** IC 147 QALY 0.02  ICUR 7211  S**ocietal**  **CUA**   IC +35.47 QALY 0.02 ICUR 1717  (GBP, 2018-19) | **Societal**  2101  (42.83)    **Health**  8707  (177.49) |
| Warmerdam, 2010, Netherlands | **Mental Health**  (Depression) 252 participants aged 18+, presenting with depressive symptoms, and willing to participate in a self-help course. Presence of depressive symptoms based on (CES-D) score 16+ | **Behavioural**  Internet CBT with therapist support vs Waitlist control  Problem solving therapy vs Waitlist control | QALY; change in severity of depression CES-D  (12 weeks) | Societal; CEA & CUA; Community | **Internet CBT with therapist support vs Waitlist control**  **CEA** IC 256 depression +0.135 ICER1817  **CUA** IC 256 QALY 0.01 ICUR 22609  (EUR, 2007) | **Societal**  30094  (300.94) |
| Zhou, 2025, China | **Mental Health**  (Depression) 244 adults with Major depressive disorder (MDD) | **Behavioural**  Unguided internet-based CBT (ICBT) + usual care vs WLC (usual care) | QALY (1 year) | Health & Societal; CUA, Outpatients | **Societal**  **CUA** IC -368.02 QALY 0.0278 ICUR  -13235.44  **Health**  **CUA** IC 9.33 QALY 0.0228 ICUR 409.25  (US, 2022) | **Health**  294.89  (6.72) |

^a^Intervention was listed only if there was more than one comparison

^b^based on costs converted to GBP 2023

Abbreviations: QALY= Quality-adjusted life year; CEA= Cost effectiveness analysis; CUA= Cost Utility analysis; ICER = Incremental cost-effectiveness ratio; ICUR=Incremental cost utility ratio;  CBT= cognitive behavioural therapy; HV=Health visitor; PC=patient centred GP= General practitioner; CORE-OM=Clinical Outcomes in Routine Evaluation-Outcome Measure; CSR=Clinician-rated symptom severity; SMFQ= CES-D= Center for Epidemiological studies Depression Scale; GAD-7=Generalized Anxiety Disorder-7; RCI=Reliable change index

*Price year estimated as it was not reported. Estimates are 3 years prior to the year the study was published.

**Supplementary Table 9.** Characteristics of studies and CUA interventions that cost less and are less effective (n=9 interventions)

| **Study, Year, Country, Design** | **Category (condition); Population** | **Intervention type; Intervention vs Comparator** | **Outcomes**  **(Time horizon)** | **Perspective, Analysis, Setting** | **Results (Incremental costs (IC); QALY gained/health outcome; ICER/ICUR)** | **Graph details; Perspective, Intervention^a^ ICUR^b^ (costs in GBP 2023)** |
| --- | --- | --- | --- | --- | --- | --- |
| Axelsson, 2020, Sweden | M**ental health** (Health Anxiety)   204 adults 18+ years with principal health anxiety | **Behavioural**  Internet CBT (self- help with therapist feedback/support) vs individual face to face CBT | QALY; change in health anxiety-HAI (12 months) | Societal; CEA & CUA; Primary care | **CEA**: IC: -6127; HAI: -0.16; ICER 39 057  **CUA**: IC -6127; QALY: -0.0095; ICUR   643516  (USD 2017, baseline to 12- month follow-up) | **Societal**  548716  (-5213) |
| Creswell, 2024, UK | **Mental Health** (Anxiety)  706 Children 5-12 years and have a problem with anxiety as determined by clinical teams in usual practice) | **Behavioural**  OSI (online support and intervention-parent led CBT) vs TAU | QALY (26 weeks) | Health System; CUA; Primary care (mental health services) | CUA IC-85.87 QALY -0.0067 ICUR 12883  (GBP, 2020-21) | **Health**  14306  (-96) |
| Domino, 2009, United States | **Mental Health**  (Depression)  327 Outpatient adolescents aged 12 to 18 years with a primary diagnosis of major depression | **Behavioural**  **3 arm;**  CBT with therapist vs Antidepressant  (Fluoxetine)  CBT + Fluoxetine vs Fluoxetine | QALY; Depression free days-(DFD)  (36 weeks) | Societal; CEA & CUA; Community/Outpatients | **CBT with therapist vs Fluoxetine**    **CEA** IC -1044 DFD -19.4 ICER NR  **CUA** IC -1044 QALY -0.02 ICUR NR (52200)  (USD, 2003) | **Societal**    **CBT with therapist vs Fluoxetine**  57916  (-1158) |
| Duarte, 2017, United Kingdom | **Mental Health**  (Depression) REEACT trial,  461 adults presenting with depression according to a self-report questionnaire [score of >10 on the Patient Health Questionnaire (PHQ-9) depression severity instrument | **Behavioural**  **3 arm;**  Computerised CBT (Beating the Blues)    + UC vs UC  Computerised CBT (MoodGYM) + UC vs UC | QALY  (24 months) | Health System; CUA;  Primary Care | **Beating the Blues vs UC**  **CUA** IC 104.24 QALY -0.0435 ICUR Dominated    **MoodGYM vs UC**  **CUA** IC -106.07 QALY -0.0153 ICUR 6933    (GBP, 2011/12, discounted 3.5% from 12 to 24 months | **Health**    **MoodGYM**  9339  (-143) |
| El Alaoui, 2017, Sweden | **Mental Health**  (Social Anxiety Disorder) 126 participants with social anxiety disorder | **Behavioural**  Internet CBT (with therapist support) vs Group face to face CBT | QALY  (4 years) | Health system; CUA;  Hospital/Outpatient clinic | **CUA** IC -343 QALY -0.18 ICUR NR (1906)  (EUR, 2017, (above not discounted) discounted 3% & 5% per annum as a sensitivity analysis)) | **Health**  2046  (-368) |
| Gerhards, 2010, Netherlands | **Mental Health**  (Depression) 303 Adults aged 18 to 65 years with mild to moderate depressive complaints (Beck Depression Inventory II (BDI–II) score>=16) | **Behavioural**  Computerised CBT (Colour your life) vs Usual GP care  Computerised CBT (Colour your life) +Usual GP care vs Usual GP care | QALY -EQ-5D & SF-6D; severity of depression -BDI-II  (12 months) | Societal; CEA & CUA,  Community | **Computerised CBT (Colour your life) vs Usual GP care;**    **CEA** IC -1784 BDI-II +1.33 ICER NR  **CUA** IC -1784 QALY(EQ-5D) -0.01 ICUR NR (178400)    (EUR, 2007) | **Societal**  209717  (-2097) |
| Ginsburg, 2022, United States | **Mental Health**  (Anxiety)” STARS” RCT with 216 students aged 6 to 18 years diagnosed with anxiety based on the ADIS | **Behavioural**  School based modular CBT vs school clinician care | QALY  (12 months) | Societal; CUA; Community | **CUA** IC -166 QALY -0.024 ICUR 6917  (USD, 2018)  *also did severity of anxiety subgroup  **Severe Anxiety;**  **CUA** IC -594 QALY +0.026 ICUR -22846  (USD, 2018) | **Societal**  5747  (-138) |
| Holst, 2018, Sweden | **Mental Health**  (Depression) 90 patients aged ≥18 years with mild to moderate depression. Patients had to have a MADRS-S score <35, which is the cut-off point for severe depression | **Behavioural**  Internet CBT with therapist support vs Usual GP care | QALY; mean change in BDI-II  (12 months) | Societal & Health System; CEA & CUA; Primary Care | **Societal;**  CEA IC -281 BDI-II -0.46 ICER 411  CUA IC -281 QALY -0.05 ICUR 5387  **Health**  CUA IC –26.85 QALY-0.05 ICUR 537  (EUR, 2013) | **Societal**  6201  (-310)    **Health**   593  (-29.63) |
| Van Reijen, 2022,  Netherlands | **Cardiovascular**  (Intermittent Claudication) 206 Adult patients with disabling IC intermittent claudication) with a max walking distance between 100 and 300 metres on a treadmill | **Exercise/Physical Therapy**  Supervised exercise therapy (SET) vs Endovascular Revascularisation (ER) | QALY; Change in VasQol score  (12 months) | Restricted Societal; CEA & CUA; Allied Health/Hospital | **CEA** IC -1852 VasQol -0.64 ICER 2877  **CUA** IC -1852 QALY -0.09 ICUR 20805  (EUR, 2015) | **Societal**  22474  (-2023) |

^a^Intervention was listed only if there was more than one comparison.

^b^based on costs converted to GBP 2023.

Abbreviations: QALY= Quality-adjusted life year; CEA= Cost effectiveness analysis; CUA= Cost Utility analysis; ICER = Incremental cost-effectiveness ratio; ICUR=Incremental cost utility ratio;  ; CBT= cognitive behavioural therapy; HV=Health visitor; PC=patient centred GP= General practitioner; CORE-OM=Clinical Outcomes in Routine Evaluation-Outcome Measure; CSR=Clinician-rated symptom severity; SMFQ= CES-D= Center for Epidemiological studies Depression Scale; GAD-7=Generalized Anxiety Disorder-7; RCI=Reliable change index

**Supplementary Table 10.** Characteristic of studies and CUA interventions that cost more and less effective (n=7 interventions)

| **Study, Year, Country, Design** | **Category (condition); Population** | **Intervention type; Intervention vs Comparator** | **Outcomes**  **(Time Horizon)** | **Perspective, Analysis, Setting** | **Results (Incremental costs (IC); QALY gained/health outcome; ICER/ICUR)** | **Graph details; Perspective, Intervention^a^ ICUR^b^ (costs in GBP 2023)** |
| --- | --- | --- | --- | --- | --- | --- |
| Bodden, 2008, Netherlands | **Mental Health** (Anxiety) 116 children aged 8-18 years with a primary anxiety disorder | **Behavioural**  Family CBT vs individual CBT | QALY; proportion of anxiety free at 1 year-ADIS   (15 months) | Societal; CEA & CUA; Hospitals are the Trial centres (multi-centre) | **CEA** IC 299; proportion of anxiety free -0.15; ICER inferior  **CUA** IC 299; QALY -0.02; ICUR inferior (EUR, 2003, 4% discounted after 1 year) | **Societal**  -18991  (380) |
| Duarte, 2017, United Kingdom | **Mental Health**  (Depression) REEACT trial; 461 adults presenting with depression according to a self-report questionnaire [score of >10 on the Patient Health Questionnaire (PHQ-9) depression severity instrument | **Behavioural**  3 arm**;**  Computerised CBT (Beating the Blues)    + UC vs UC  Computerised CBT (MoodGYM) + UC vs UC | QALY  (24 months) | Health System; CUA;  Primary Care | **Beating the Blues vs UC**  **CUA** IC 104.24 QALY -0.0435 ICUR Dominated (-2396)  **MoodGYM vs UC**  **CUA** IC -106.07 QALY -0.0153 ICUR 6933  (GBP, 2011/12, discounted 3.5% from 12 to 24 months | **Health**    **Beating the Blues**  -3228  (140) |
| Kigozi, 2018, UK | **Musculoskeletal**   (knee OA) 514 adults 45+ with current knee pain and /or stiffness in one or both knees who met criteria by NICE for knee OA | **Exercise/Physical Therapy**  2 different Enhanced physical therapy interventions; (individually tailored~ITE or targeted exercise adherence ~TEA) vs usual physical therapy (UC) | QALY  (18 months) | Health care; CUA; Primary care/Allied health | **ITE vs UC**  CUA IC 273.4 QALY -0.015 ICUR -18227  **TEA vs UC**  CUA IC 141.80 QALY -0.003 ICUR -47267  (GBP, 2012-13) | **Health**    **ITE vs UC**  -24197  (363)  **TEA vs UC**  **-**62750  (188) |
| Smeets, 2009, Netherlands | **Musculoskeletal**  (Chronic low back pain) 172 adults aged 18 to 65 years with non-specific low back pain for more than three months resulting in disability (Roland Disability Questionnaire score [RDQ] > 3) and ability to walk at least 100 m. | **Exercise/Physical Therapy**  Combination of Active physical (APT) + graded activity with problem solving (GAP) vs APT  Combination APT + GAP vs GAP | QALY; change in RDQ-Roland Disability Questionnaire)  (52 weeks) | Societal; CEA & CUA; Primary Care/Outpatient | **APT + GAP vs APT**  **CEA** IC -407 RDQ -1.23 ICER 371  **CUA** IC -407 QALY -0.014 ICUR 35 060  **APT +GAP vs GAP**  **CEA** IC 4787 RDQ -1.27 ICER -3759  **CUA** IC 4787 QALY -0.045 ICUR -108,857  (EUR, 2003) | **Societal**    **APT + GAP vs GAP**    135132  (6081) |
| Smit, 2013, Netherlands | **Mental Health**  (Smoking Cessation) 414 smokers aged 18+ | **Behavioural**  3 arm intervention; Internet-based multiple computer-tailored program (MT) vs MT + counselling (C) vs Usual care (UC) | QALY; prolonged abstinence (PA) at 12 months  (12 months) | Societal; CEA & CUA; Primary care | **MT vs UC**  CEA IC 255 PA 0.05 ICER 5100 CUA IC 255 QALY -0.01   ICUR -25500  **MT +C vs UC**   CEA IC 806 PA -0.02 ICER  CUA IC 806 QALY 0.02 ICUR 40300 (EUR, 2011) | **Societal**    **MT vs UC**    -28907  (289) |
| Stallard, 2013, United Kingdom | **Mental Health**  (Depression) 846 Young people in Year groups 8 to 11 (ages 12–16 years) at high risk of depression (Those with SMFQ scores of ≥ 5 at both screening and baseline assessments) were classified as high risk. Those who attended Personal**,** Social and Health Education (PSHE) at participating schools were eligible | **Behavioural**  Classroom-based CBT vs  Usual school curriculum delivered by teachers | QALY; change in SMFQ  (12 months) | Societal; CEA & CUA; Community | **CEA** IC 147 SMFQ -0.58 ICER more costly/less effective) -253  **CUA** IC 147 QALY-0.009 ICUR -15667  (GBP, 2010) | **Societal**  -22471  (202) |
| Stanczyk, 2014, Netherlands | **Mental Health** (Smoking Cessation) 2099 adults smokers 18+ and motivated to quit smoking within 6 months | **Behavioural**  3 interventions; video-based vs text-based computer-tailored smoking cessation interventions vs control | QALY; prolonged abstinence (PA)   (12 months) | Societal; CEA & CUA; Primary care | **Video vs control**  CEA IC 60 PA +0.004 ICER 1500  CUA IC 60 QALY 0.001 ICUR 60000  **Text vs Control**  CEA IC 504 QALY +0.01 ICER 50 400  CUA IC 504 QALY -0.002 ICUR-252000  (EUR, 2013) | **Societal**    **Text vs control**;  -278045  (556) |

^a^Intervention was listed only if there was more than one comparison.

^b^based on costs converted to GBP 2023

Abbreviations: QALY= Quality-adjusted life year; CEA= Cost effectiveness analysis; CUA= Cost Utility analysis; ICER = Incremental cost-effectiveness ratio; ICUR=Incremental cost utility ratio; CBT= cognitive behavioural therapy; SMFQ= Short Mood and Feelings Questionnaire; ADIS=Anxiety Disorders Interview Schedule, UC=usual care

**Supplementary Table 11.** Characteristics of studies only completing cost effectiveness analyses (CEA) using ICER’s (25 CEA) + 1 that used CBA

| **Study, Year, Country, Design** | **Category (condition); Population** | **Intervention type; Intervention vs Comparator** | **Outcomes**  **(Time horizon)** | **Perspective, Analysis, Setting** | | | **Results (Incremental costs (IC); QALY gained/health outcome; ICER/ICUR)** | **ICER (cost/health outcome)** |
| --- | --- | --- | --- | --- | --- | --- | --- | --- |
| **Studies with interventions that cost less but are more effective** | | | | | | | | |
| Aranda-Reneo, 2021, Spain | **Musculoskeletal**   (Falls) 498 adults aged 65 to 80 | **Exercise/Physical Therapy**  Group Otago exercise program vs Individual Otago exercise program | risk of falling based on SPPB (short physical performance battery protocol), Time up and go, and Tinetti  (12 months) | Health care; CEA; Primary care | | | CEA IC -52.35 SPPB 0.08 ICER Dominant (hand calculated as-654.375   (EUR, 2019) | Costs less/more effective  -654.375 |
| Bergstrom, 2010, Sweden | **Mental Health**  (Panic disorder); 104 Adults 18+ years with panic disorder (with or without agoraphobia) | **Behavioural**  Internet CBT (with therapist guided support) vs group face to face CBT | Proportion of panic disorder severity score (PDSS) responders at follow-up  (6 months) | Health system; CEA; Hospital was trial centre | | | **CEA** IC -239; proportion of PDSS responders; +0.06; ICER 121  (EUR, 2007*) | Costs less/more effective  -121 |
| de Boer, 2014, Netherlands | **Mental Health**  (Chronic pain) 50 patients 18+ who have nonspecific chronic pain for which no somatic treatments could be offered | **Behavioural**  Internet CBT (therapist support) vs Group face to face CBT | Pain-pain catastrophizing scale-PCS  (16 weeks) | Health system; CEA; Hospital is the Trial Centre | | | **CEA** IC -199 PCS +5 ICER 40  (EUR, 2012) | Costs less/more effective  -40 |
| Hupperets, 2010,  Netherlands | **Musculoskeletal** (prevent ankle sprains) 522 athletes aged 12-70 who had sustained a lateral ankle sprain up to two months before inclusion | **Exercise/Physical Therapy** Proprioceptive training program + usual care vs Usual care | proportion of ankle sprain recurrence prevented   (12 months) | Societal; CEA; Primary care | | | CEA IC -68.60 ankle sprain recurrence prevented +0.1158 ICER -592   (EUR, 2007*) | Costs less/more effective  -592 |
| Janssen, 2014, Netherlands | **Musculoskeletal**  (Ankle sprain) 340 Athletes 18 to 70 years of age who had sustained an ankle sprain no longer than 2 months prior and continue to participate in sports at least 1 h/week | **Exercise/Physical Therapy**  neuromuscular training (home-based) vs combined Brace + neuromuscular training (home-based)  Brace vs combined Brace + neuromuscular training (home-based) | Recurrence of ankle sprain  (12 months) | Societal; CEA Community | | | **Neuromuscular training (home-based) vs combined**  CEA IC -28.37 sprains +0.0915 ICER 310.08  **Brace vs combined**  CEA IC -76.16 sprains -0.0268  ICER -2828.3    (EUR, 2013) | **Bracing dominant** Costs less/more effective (fewer mean sprains)  -2828.3 |
| Patil, 2015, Finland | **Musculoskeletal**   (Falls) 409 community-dwelling, independently living Finnish women aged 70 to 80 yrs | **Exercise/Physical Therapy**   4 groups~ no exercise + placebo, no exercise + vit D, exercise +placebo, exercise + vit D | injurious falls per person year;  (24 months) | Societal; CEA; Primary care | | | exercise + placebo vs no exercise +placebo (essentially exercise vs no exercise CEA IC -133.5 Effect 0.05 ICER -2670  (EUR, 2011) | costs less/ more effective  -2670 |
| Rizzo, 1996, USA | **Musculoskeletal** (Falls) 301 participants 70+ years and had at least 1 of 8 targeted risk factors for falling. | **Exercise/Physical Therapy** Multiprogram targeted intervention [exercise (based on baseline) + behaviour recommendations and medication adjustment if required] vs usual care (series of home visits by social work student) | Medical Falls prevented (aggregate)   (12 months) | Perspective not reported~Health system assumed; CEA; Primary care | | | CEA IC -2129 Medical Falls prevented (aggregate) 13   ICER -163.77   (US, 1993) | Costs less/more effective  -163.77 |
| Simpson, 2003, UK | **Mental Health** (Depression) 145 patients age 18 to 70 were recruited at 7 GP practices if scored between 14 to 40 points on the BDI and had been depressed for >=6 months. | **Behavioural**  Psychodynamic counselling + Usual care by GP  vs control + usual care by GP | proportion of "non cases" at 12 months based on BDI (Beck Depression Inventory)   (12 months) | Health System; CEA; Primary care | | | CEA IC -28 BDI non-cases +0.11 ICER -254.5  (GBP, 1997-98) | costs less/more effective  -254.5 |
| Smit, 2006, Netherlands | **Mental Health**  (Depression) 216 adults (aged 18–65 years) with sub-threshold depression defined as having at least one core symptom plus one, two or three current depressive symptoms according to the Instel screening instrument but not those with full-blown depression. | **Behavioural**  cognitive-behavioural minimal contact psychotherapy vs usual GP care | % of people who did not get depression  (12 months) | Societal; CEA; Primary Care | | | **CEA** IC -1848 % no depression +6.4  ICER -288.75  (EUR, 2003) | Costs less/ more effective  -288.75 |
| Thiart, 2016, Germany | **Mental Health**  (Insomnia) 128 currently employed schoolteachers aged 18+ with clinically significant insomnia symptoms (ISI>14) and elevated work related rumination (Irritation scale, subscale "Cognitive Irritation">14) | **Behavioural**  Internet-based CBT with therapist support (GET.ON Recovery)+ Usual GP care vs Usual GP care Waitlist control | ISI-Insomnia severity index; RCI-Reliable change index  (6 months) | Employers; CEA & CBA;  Community | | | **CEA** IC -417.63 ISI +0.36 ICER -1512  CBA ROI 208.71  (EUR, 2013) | Costs less/more effective  -1512 |
| Van Apeldoorn, 2014, Netherlands | **Mental Health**  (Panic disorder) 150 patients between 18 and 65 years of age with panic disorder with or without agoraphobia | **Behavioural**  CBT with therapist vs Anti-depressant (SSRI) | Change in mean HAM-A – Hamilton Anxiety rating scale)  (24 months) | Societal; CEA, Primary Care/Allied Health/Specialist | | | **CEA** IC -894 HAM-A +3.9 improvement   ICER NR ( -229.23)  (EUR, 2005) | Costs less/more effective  -229.23 |
| **Studies with interventions that cost less and are just as effective** | | | | | | | | |
| King, 2000, United Kingdom | **Mental Health**  (Depression and/or Anxiety) 197 Adults 18 +years diagnosed with depression or mixed anxiety and depression. As assessed by a score of 14+ on the BDI | **Behavioural**  CBT with therapist vs Usual GP care  CBT with therapist vs non-directive counselling | Reduction in depression/anxiety scores-BDI  (12 months) | Societal; CEA; Primary Care | | | **CBT with therapist vs Usual GP care**  **CEA** IC -157 BDI -0.9 ICER 174  (GBP, 1997/98)  *No significant differences in cost and effectiveness | Costs less/just as effective  174 |
| **Studies with interventions that cost less and are less effective** | | | | | | | | |
| Roberge, 2008, Canada | **Mental Health**  (Panic disorder with Agoraphobia) 100 adults from 19 to 65 years suffering from PDA according to the Diagnostic and Statistical Manual of Mental Disorders, fourth edition (DSM–IV) criteria | **Behavioural**  3 interventions; Standard CBT (individual F2F CBT), Brief (Brief individual CBT and Group CBT | Change in Global Functioning Index-GFI  (3 months) | Societal; CEA; Primary Care/Community/Hospital | | | **Brief CBT vs Standard CBT**  **CEA** IC -708.19 GFI -4.6 ICER 153.58  (CAD, 2000) | Costs less/slightly less effective  153.58 |
| **Studies with interventions that cost more and are more effective** | | | | | | | | |
| Boggs, 2021, USA | **Mental Health** (Depression) 389 adult patients with at least one prior episode of major depressive episode | **Behavioural**  Mindful mood balance (mindfulness-based cognitive therapy)~web based program  + usual care vs Usual care | DFD; (12 months) | Health service; CEA; Primary care | | | CEA IC 432 DFD +29 ICER 14.89  (USA, 2015) | Cost more/more effective   14.89 |
| Hewitt, 2019, Australia | **Musculoskeletal**  (Falls) 16 RCFs/221 Adults living in residential care facilities with a mean age of 86 years (SUNBEAM programme) | **Exercise/Physical Therapy**  Exercise program vs UC | Falls avoided  (12 months) | Health system; CEA; Residential Aged Care Facility | | | **CEA** IC 28.29 Falls avoided 1.31 ICER 22    (AUD, 2015) | Costs more/more effective  22 |
| Holman, 2011, United Kingdom | **Mental Health**  (Depression) 198 Patients 65 years or older with a primary diagnosis of depressive disorder obtained from the Geriatric Mental State and History and Etiology Schedule and a score of 14 or higher on the BDI-II | **Behavioural**  CBT with therapist+ Usual GP care vs Usual GP care    CBT with therapist + Usual GP care vs Talking control + Usual GP care | Change in Depression score (BDI-II)  (10 months) | Health System; CEA; Primary Care | | | CBT with therapist+ Usual GP care vs Usual GP care  **CEA** IC 427 BDI-II +3.6 (reductions in depression score) ICER 120    CBT with therapist + Usual GP care vs Talking control + Usual GP care  **CEA** IC 580 BDI-II +3.5 ICER 167  (GBP, 2008) | Costs more/ more effective  120 |
| Kafali, 2014, United States | **Mental Health**  (Depression) 257 Adult Latino patients (aged 18+) with depression (scored 10 or more in PHQ-9) and met initial screening criteria for Major depressive disorder | **Behavioural**  F2F CBT with therapist vs Usual GP care  Telephone CBT vs Usual GP care  Telephone vs F2F | Reduction in depression scores -PHQ-9 & Hopkins Symptom Checklist (HSCL)  (4 months) | Societal; CEA; Primary Care | | | **F2F CBT vs UC**  CEA IC 731.86 PHQ-9 -2.30 ICER 318.20  CEA IC 731.86 HSCL -0.27 ICER 2710.59  **Telephone CBT vs UC**  CEA IC 236.76 PHQ-9 -2.98 ICER 79.45  CEA IC 236.76 HSCL -0.33 ICER 717.47  (USD, 2010) | Costs more/more effective  318.20 |
| Petrou, 2006, United Kingdom | **Mental Health**  (Postnatal depression) 151 primiparous women attending antenatal clinics at 26–28 weeks of gestation who were at high risk of developing postnatal depression using a predictive index (index score ≥ 24) | **Behavioural**  Preventive intervention (weekly counselling support) vs Usual GP care | Postnatal depression avoided  (18 months) | Societal; CEA; Primary Care | | | **CEA** IC 119.5 PND 0.49 months avoided  ICER 43.1 per month of postnatal depression avoided  (GBP, 2000, 6% discounted per annum) | Costs more/more effective  43 |
| Poirier-Bisson, 2013, Canada | **Mental Health**  (Panic disorder) 69 Adults who presented at the ED with chest pain and diagnosed with NCCP | **Behavioural**  **(3 interventions)**  Panic management (group CBT principles) vs Usual GP care  Brief individual CBT vs Usual GP care  Pharmacotherapy vs Usual GP care | change in ADIS-IV score from baseline to post-test  (6 months) | Health System; CEA;  Hospital | | | **Panic management (group CBT principles)**  **CEA** IC 174.5 ADIS-IV +1.81 ICER 124.05  (CAD 2008-2009) | Costs more /more effective  124.05 |
| Robertson, 2001, New Zealand | **Musculoskeletal**  (Falls) 211 women and men aged 75 years and older | **Exercise/Physical Therapy**  Home-based exercise programme delivered by nurse vs UC | Falls avoided  (12 months) | Societal; CEA; Primary Care/Community/Hospital as trial centres | | | **CEA** IC 432 Falls prevented +0.46   ICER 1803  (NZD, 1998-99) | Costs more/ more effective  1803 |
| Robertson, 2001, New Zealand (2) | **Musculoskeletal**  (Falls) 233 Women aged 80 years and older living in the community and invited by their general practitioner to take part | **Exercise/Physical Therapy**  Home-based exercise programme vs US | Falls avoided  (24 months) | Societal; CEA; Primary Care | | | **After 1 year;**  **CEA** IC 173 Falls prevented 64 ICER 314  **After 2 years;**  **CEA** IC 195 Falls prevented 82 ICER 265  (NZD, 1995) | Costs more/more effective  265 |
| Scott, 2003, United Kingdom | **Mental Health**  (Depression) 158 partially remitted major depression adults 21- to 65-year-old psychiatric out-patients with unipolar depression and who had satisfied DSM–III–R criteria for major depression in an episode within the past 18 months but not in the past 2 months | **Behavioural**  cognitive therapy + anti-depressant vs anti-depressant | % Relapse of Depression prevented  (17 months) | Health System; CEA; Outpatient | | | **CEA** IC 779 relapse prevented +0.18 ICER 4328  (GBP 1998/1999) | Costs more/ more effective  4328 |
| Slade, 2017, United States | **Mental Health**  (PTSD + other severe mental illness) 183 adults with diagnosis of schizophrenia, schizoaffective disorder, major depression, or bipolar disorder based on the SCI for DSM-IV Axis-I Disorders. All participants also met clinical criteria  for a DSM-IV diagnosis of PTSD as verified by (CAPS) interview (schizophrenia version) and had currently severe PTSD symptoms (that is, a minimum CAPS total score of 65) | **Behavioural**  CBT with therapist vs  Brief intervention | % remission from PTSD  (12 months) | Payer; CEA; Hospital/Outpatient | | | **CEA** IC 3991 % remission +0.108 ICER 36893  (USD, 2010) | Costs more/ more effective  36893 |
| Thomas, 2005, United Kingdom | **Musculoskeletal**  (knee pain) 759 people aged >=45 years with reported current knee pain | **Exercise/Physical Therapy**  Exercise Program vs No-exercise group control | % of those with a Change in WOMAC > 50% improvement  (2 years) | Health System & Payer; CEA; Primary Care | | | **CEA** IC 225 WOMAC +0.07 ICER 2570  (GBP, 1996, 5% discounted in the 2^nd^ year) | Costs more/ more effective  2570 |
| Xin, 2019, | **Diabetes**   (Type 2 diabetes) 298 Adults 20 to 65 years with type 2 diabetes diagnosed within previous 6 years | **Nutrition**  Counterweight Plus weight management programme + TAU vs control +TAU | % of those in remission  (12 months) | Health System; CEA; Primary care | | | CEA IC 982 diabetes remission 0.416278   ICER 2359  (GBP, 2016-17) | costs more/more effective  2359 |
| **Cost effectiveness studies only using Net Benefit Analysis** | | | | | | | | |
| Carande-Kulis, 2015, United States | **Musculoskeletal** (Falls)  278 Adults aged 65 or older with differing levels of all risk | **Exercise/Physical Therapy**  **3 interventions:**  Otago Exercise Program vs control  Tai chi vs control  Stepping on exercise program vs control | Net benefit (12 months) | | Payer; CBA; Community | **Otago Exercise Program**  NB 121.85 ROI 36%  **Tai chi**  NB 529.86 ROI  509%  **Stepping on exercise program**  NB 134.37 ROI 64% | |  |

QALY= Quality-adjusted life year; CEA= Cost effectiveness analysis; CUA= Cost Utility analysis; ICER = Incremental cost-effectiveness ratio; ICUR=Incremental cost utility ratio;   CBT= cognitive behavioural therapy; HV=Health visitor; PC=patient centred GP= General practitioner; CORE-OM=Clinical Outcomes in Routine Evaluation-Outcome Measure; CSR=Clinician-rated symptom severity; SMFQ= CES-D= Center for Epidemiological studies Depression Scale; GAD-7=Generalized Anxiety Disorder-7; RCI=Reliable change index

*Price year estimated as it was not reported. Estimates are 3 years prior to the year the study was published.

**Supplementary Figure 4**


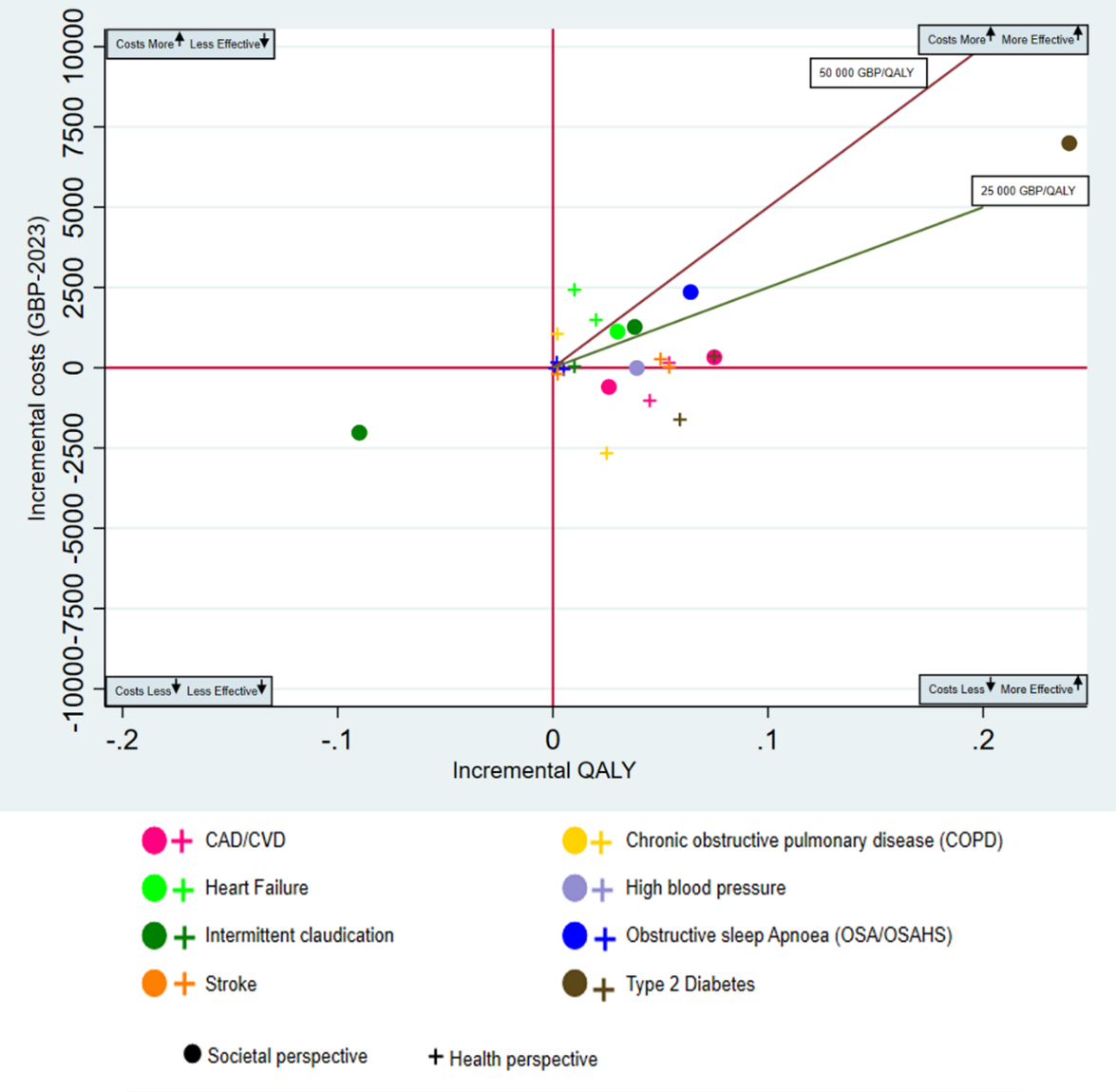


**Supplementary Figure 4.** Cost utility analysis of HANDI interventions by cardiovascular/diabetes conditions and by economic perspective. The outlier study by Yu 2004(1) is not shown.

**Supplementary Material 1**: detailed results of primary (CUA) and secondary outcomes (CEA) with references

**Primary Outcome; Cost utility Analysis (CUA)**

130 studies with a total of 150 interventions reported cost utility analyses and calculated ICURs. These are summarised by the ICUR quadrant. The ICUR quadrant results for the different categories of HANDI interventions appears in Table 1 and summary of studies (Supplementary Tables 9 to 12) while reported ICUR values appear in Supplementary Tables 4 to 7.

***Distribution of HANDI interventions across the cost effectiveness plane***

***Interventions that cost less and are more effective (dominant SE quadrant)***

Of the 150 interventions, 60 interventions (40%) showed that NDIs were dominant (less costly and more effective) compared to the reference or standard intervention (Supplementary Table 4 above and Supplementary Table 9 below).

Many of these were behavioural interventions (n=33; 55.0%). Most behavioural interventions evaluated various forms of internet or computerised cognitive behavioural therapy (CBT) compared to either a control or usual care or active interventions such as a group face-to-face CBT (Supplementary Table 2). Two additional interventions were behavioural (e.g., CBT AND physical rehabilitations versus physical rehabilitation alone) for a musculoskeletal condition (e.g., low back pain)(4, 5).

Additionally, 23 interventions (38.3%) in this group evaluated exercise/physical therapy interventions(1, 6-25). Of these, 17 interventions (73.9%) were exercise/physical therapy based for musculoskeletal conditions(6, 8-13, 15, 17-23, 25) (i.e., 6 studies for hip and or knee pain (6, 10, 17, 19, 23, 25), 4 for low back pain(9, 15, 21, 22), 4 for falls prevention(11-13, 18), and 1 for chronic fatigue syndrome(20)). Additionally, of the 23 exercise/physical therapy interventions, 5 studies evaluated exercise/physical therapy interventions for cardiovascular conditions(1, 7, 8, 14, 16), and one study (4.3%) was for a mental health condition(24).

Only two interventions (2/60; 3.3%) were nutrition-based interventions (i.e., salt substitution for CVD and weight management intervention for diabetes(26, 27)). Similar, only two studies (2/55; 3.6%) evaluated device interventions for CVD (e.g., a continuous positive airway pressure for obstructive sleep apnoea)(28, 29).

**Supplementary Table 12**. Summary of interventions that cost less and are more effective (N=60 interventions)

| Type of Intervention | Category | Condition | #studies | Details of Interventions |
| --- | --- | --- | --- | --- |
| Behavioural (n=33) | Mental Health (n=31) | Health Anxiety | 3 | Internet CBT (unguided) vs control(30) ^  Internet CBT with therapist support vs control(31) ^ or + UC vs UC(32) ^ |
|  |  | Social Anxiety Disorder (SAD) | 3 | Internet CBT with therapist support vs group F2F CBT(33) ^ or vs IDST(34) ^  Text-based CBT (StudiCare) vs waitlist control(35) ^ |
|  |  | Anxiety | 5 | Internet CBT with therapist support vs internet delivered child play(36) ^, or UC(37) ^  School based CBT vs school classroom clinician care(38) ^  Brief parent delivered CBT vs solution-focussed brief therapy(39) ^  BACT vs BI CBT(40) ^ |
|  |  | Depression | 11 | CBT vs Usual GP care(41, 42) *, CBT vs antidepressant(43) *, 1 day CBT workshop vs WLC(44) ^ , + UC vs UC(45) ^, internet CBT with no therapist support + Usual GP care vs UGPC(46) ^ or vs WLC(47, 48) *^, computer assisted CBT vs F2F CBT(49) ^, web-based CDMIs vs UC(50) *^Mindfulness-based CBT vs TAU^(3) |
|  |  | Anxiety and / or Depression | 4 | CBT vs Usual GP care(51) ^, BBT vs ARC(52)^, computerised CBT (Beating the Blues) + Usual GP care vs Usual GP care(53) ^, MBCT-SH vs CBT-SH(54) * |
|  |  | Insomnia | 3 | Internet guided CBT vs TAU(55, 56) ^, Internet CBT with therapist vs Group F2F CBT(57) ^ |
|  |  | Panic Disorder | 1 | Individual CBT with therapist vs Usual GP care(58) ^ |
|  |  | PTSD | 1 | Prolonged exposure therapy vs Pharmacotherapy with Sertraline(59) ^ |
|  | Musculoskeletal (n=2) | Low Back Pain | 2 | CBT + SPR vs SPR(5) ^, CBT (exposure in vivo) vs graded activity (4) ^ |
| Exercise/Physical Therapy (n=23) | Musculoskeletal (n=17) | Hip and/or Knee OA/PFPS | 6 | Exercise vs UC(6) *^, Group water-based exercise vs control(10) ^, F2F physio + web app vs Usual physio(17) *^, Class-based exercise program + Home-based vs Home-based(19) *, Supervised exercise program vs UC(23) ^strength exercise vs UC*^, aerobic exercise vs UC^(25) |
|  |  | Low back pain | 4 | Yoga vs UC(9)* or waitlist control(15) ^, Pilates once per week vs control (booklet)(21) ^, exercise program vs control(22) ^ |
|  |  | Fall prevention | 4 | Otago exercise program + UC vs UC(11, 12) *, Resistance exercise 1x, or 2x per week vs balance and tone(13) *, Tai J Quan vs stretching(18) * |
|  |  | CFS | 1 | Graded exercise therapy (GET) vs Specialist Medical Care (SMC)(20) ^ |
|  | Cardiovascular (n=5) | CAD with or without ACS | 3 | Internet based tele rehab vs Usual rehab(14) ^, Exercise-based cardiac rehab vs Usual GP Care(16) *, CRPP (exercise + education) vs control (no exercise)(1) * |
|  |  | Stroke | 1 | Exercise program vs control (balance and tone)(7) * |
|  |  | COPD | 1 | Home-based exercise rehab + usual GP care vs Usual GP care(8) *^ |
|  | Mental Health (n=1) | Depression | 1 | Mindful yoga + TAU vs TAU(24) ^ |
| Nutrition(n=2) | Cardiovascular (n=1) | High Blood pressure prevention | 1 | Salt substitute vs control (regular salt)(26) ^ |
|  | Diabetes (n=1) | Type 2 Diabetes | 1 | Weight management (Counterweight Plus) + TAU vs TAU(60) * |
| Device (n=2) | Cardiovascular (n=2) | OSA/OSAHS | 2 | CPAP + BSC vs BSC(28) *  Boil & bite MAD or patient moulded MAD vs control(29) * |

*Health system perspective ^Societal perspective

Abbreviations; GP= SPR=standard physical rehabilitation, BSC= best supportive care, TAU= Treatment as usual, BACT= Blended acceptance and commitment therapy, BI= Brief individual, IDST=internet delivered supportive therapy, CBT= cognitive behavioural therapy, OA=Osteoarthritis, F2F= Face-to-face, PFPS= , PTSD= COPD= , CFS= CAD=, ACS= OSA= OSAHS= BBT= Brief behavioural therapy , ARC=assisted referral to community outpatient mental health care, MBCT-SH= Mindfulness-Based cognitive therapy-Self-help, CBT-SH=Cognitive behavioural therapy-self-help, WLC=wait-list control, CDMI= complaint-directed min-interventions

***Interventions that cost more but more effective (NE quadrant)***

Of the 150 CUA interventions, 74 (49.3%) found that NDIs cost more but are still more effective (Intervention had higher QALYs). Most of these (n=34) were exercise/physical therapy interventions(2, 9, 11, 15, 20, 21, 25, 61-87) and behavioural interventions (n=33)(30, 34, 35, 46, 48, 56, 88-112). Further, 4 studies evaluated device interventions(29, 113-115), and one study was a nutritional intervention(116) while another was under the other category (an emollient)(117). (refer to Supplementary Table 5 and Supplementary Table 10)

**Supplementary Table 13.** Summary of interventions that cost more and are more effective (N=74 interventions)

| **Type of Intervention** | **Category** | **Condition** | **#studies** | **Details of Interventions** |
| --- | --- | --- | --- | --- |
| Behavioural (n=33) | Mental Health (n=32) | Health Anxiety | 2 | Bibliotherapy CBT vs WLC^*, Internet CBT (unguided) vs WLC*, or Internet CBT (guided) vs WLC(30) ^*, Internet CBT with therapist support vs Behavioural stress management(96) ^ |
|  |  | Social Anxiety Disorder (SAD) | 4 | CBT vs WLC (91) ^P, (94)^ ^, Internet CBT with therapist support vs group F2F CBT or vs IDST(34)*  Text-based CBT (StudiCare) vs WLC(35)* |
|  |  | Anxiety | 2 | Transdiagnostic CBT group therapy + TAU vs TAU(90)*^  Internet CBT with therapist support vs WLC(93)* |
|  |  | Depression | 16 | CBT vs UGPC(97)*, CBT vs CR(106)*, Internet CBT vs TAU(101)*^, Internet CBT guided by nurse’s vs ODF(105)* Internet CBT with own therapist + UGPC vs UGPC(98)*, Internet CBT with therapist support vs UGPC(99)^,(46)^, or vs WLC(109)*,(110)^, Group F2F CBT vs UGPC(103)^, Preventative Cognitive therapy(supported self- help) vs UC(88)*^(92)*^, Internet CBT + problem solving therapy) vs Enhanced UC(89)*^, Internet problem-solving therapy vs WLC +self-help book(100)^, computer-assisted CBT vs TAU*(111) unguided internet CBT + UC vs UC (WLC)*(48) |
|  |  | Anxiety and / or Depression | 2 | CBT vs Usual GP care(95)^, Concise care (SSRI or CBT up to only 7 weeks) vs Standard care(104)^ |
|  |  | Insomnia | 4 | Internet guided CBT vs TAU(55)*, Internet CBT with therapist support + Usual GP care vs Usual GP care + WLC(56)*, CBT workshop vs WLC(118)^, sleep restriction CBT vs control (sleep hygiene)*(112)CBT with therapist vs control*(119) |
|  |  | Smoking Cessation | 2 | Video-based computer tailored program vs control(108)^, Computer tailored program + counselling vs UC(107)^ |
|  | Musculoskeletal (n=1) | Depression | 1 | Group F2F CBT + UGPC vs UGPC(102)^ |
| Exercise/Physical Therapy (n=34) | Musculoskeletal (n=19) | Hip and/or Knee OA/or knee pain | 7 | Strengthening exercise vs standard care (leaflet)(64)*  Supervised exercise therapy with pain coping skills vs just pain coping skills(65)^, Hand exercises vs control(79)*, Aquatic classes vs control(2)^, Exercise Physiotherapy vs Usual GP care(80)*^, Physical therapy vs glucocorticoid injections(82)* aerobic exercise vs UC*(25) |
|  |  | Low back pain | 6 | Yoga vs UC(9)* or waitlist control(15)*, Multimodal therapy (exercise training + behavioural support) vs TAU(72) ^P^, E-exercise (smartphone app integrated into F2F physio) vs F2F physio(101)*^, Pilates 2x /Pilates 3x per week vs control (booklet)(21)^*, BOOST programme vs BPA(87) |
|  |  | Fall prevention | 4 | E-health exercise program + UC vs UC(62)*  Otago exercise program + UC vs UC(11) (*women), Multifactorial fall prevention program vs UC(77)*, Physiotherapy (Exergames) vs SC (Leaflet)(83)* |
|  |  | CFS | 1 | Graded exercise therapy (GET) vs Specialist Medical Care (SMC) (20) * |
|  |  | Various | 1 | National Exercise Referral scheme) vs UC(78)* |
|  | Cardiovascular (n=9) | CD or CVD | 2 | Progressively autonomous physical activity vs standard supervised physical activity(63)*  Lifestyle modification program +UC vs UC(69)^ |
|  |  | Heart Failure | 3 | Rehabilitation group vs control(67)*  Supervised centre-based exercise training + disease management program (UC) vs UC(76)*  Exercise training (HF-ACTION) + Usual GP care vs Usual GP care(81)^ |
|  |  | Intermittent claudication | 2 | Home-based structured exercise (HSEP) vs Walk advice (WA)(85)*, Supervised exercise therapy vs Unsupervised walking advice(86)^ |
|  |  | Stroke | 1 | High Intensity exercise vs conventional physical therapy(73)* |
|  |  | COPD | 1 | Exercise + Education vs TAU(70)* |
|  | Mental Health (n=4) | Depression | 4 | Physical Exercise + TAU vs TAU(66)*, Physical Exercise vs TAU(101)*^ Supervised walking program vs Usual GP care(71)*, Preferred intensity exercise + TAU vs TAU(84)* |
|  | Diabetes (2) | Type 2 Diabetes | 2 | WBV-based exercise therapy + TAU vs TAU(61)*  Resistance exercise vs WTC and Aerobic exercise vs WTC(68)^ |
| Exercise + Behavioural (n=1) | Musculoskeletal | Low back pain | 1 | Group Exercise + Group F2F CBT vs Usual GP care(74) * |
| Device (n=4) | Musculoskeletal (n=2) | OA | 2 | Splint vs SM(113)*  Knee brace + UC vs UC(115)^ |
|  | Cardiovascular (n=2) | OSA/OSAHS | 2 | MAD vs CPAP(114)^  Bespoke MAD vs control(29)* |
| Nutrition (n=1) | Cardiovascular (n=1) | Stroke prevention | 1 | Salt substitute vs control (regular salt)(116)* |
| Other (n=1) | Children (n=1) | Eczema | 1 | Applying daily emollients in the first year to prevent eczema +advice vs control (advice only)*(117) |

*Health system perspective

^ Societal perspective

^P^ Payer perspective

Abbreviations; GP= SPR=standard physical rehabilitation, BSC= best supportive care, TAU= Treatment as usual, BACT= Blended acceptance and commitment therapy, BI= Brief individual, IDST=internet delivered supportive therapy, CBT= cognitive behavioural therapy, OA=Osteoarthritis, F2F= Face-to-face, PFPS= , PTSD= COPD= , CFS= CAD=, ACS= OSA= OSAHS= BBT= Brief behavioural therapy , ARC=assisted referral to community outpatient mental health care, MBCT-SH= Mindfulness-Based cognitive therapy-Self-help, CBT-SH=Cognitive behavioural therapy-self-help, WLC=wait-list control, CDMI= complaint-directed min-interventions, WBV=Whole body vibration, HSEP= Home-based structured exercise program, WA=Walk advice, BPA= Best practice advice, ODF= Online discussion forum, UGPC=Usual GP care

***Interventions that cost less and were less effective***

Only 9 (6.0 %) of the 150 CUA interventions cost less and were less effective(38, 120-127). Eight studies evaluated behavioural interventions for mental health(38, 120-126) which mostly compared cognitive behaviour therapy with different forms of CBT or compared CBT with usual care. The other study involved an exercise/physical therapy intervention for CVD(127)which specifically compared supervised exercise therapy with endovascular revascularisation (ER) for intermittent claudication due to iliac artery obstruction. (Supplementary Table 6 and Supplementary Table 11).

**Supplementary Table 14.** Summary of interventions that cost less but are less effective (N=9 interventions)

| **Type of Intervention** | **Category** | **Condition** | **# studies** | **Details of interventions** |
| --- | --- | --- | --- | --- |
| Behavioural  (n=8) | Mental Health(n=8) | Health Anxiety | 1 | Internet CBT (self-help with therapist support) vs individual F2F CBT(120) ^ |
|  |  | Social Anxiety Disorder (SAD) | 1 | Internet CBT (with therapist support) vs Group F2F CBT(124) * |
|  |  | Anxiety | 2 | Online parent led CBT (with support) vs TAU(121) *, School based CBT vs school clinician care(38) ^ |
|  |  | Depression | 4 | Internet CBT with therapist support vs Usual GP care(126) *^ CBT with therapist vs Fluoxetine(122) ^, Computerized CBT (MoodGym)(123) * (Colour your life)(125) ^ vs UC |
| Exercise/Physical Therapy (n=1) | Cardiovascular  (n=1) | Intermittent Claudication | 1 | Supervised exercise therapy (SET) vs Endovascular revascularization (ER)(127) ^ |

*Health system perspective

^ Societal perspective

Abbreviations; CBT=cognitive behavioural therapy, F2F=face-to-face, UC=Usual care, TAU=Treatment as usual

***Interventions that cost more and were less effective***

Only 7 out of the 150 CUA interventions (4.7 %) cost more and were less effective(108, 123, 128-132). Most of these studies (n=5, 71%) were behavioural interventions for mental health (i.e., anxiety, depression)(108, 123, 128, 131, 132) such as family CBT compared with individual CBT in children with anxiety(128). Two studies evaluated exercise/physical therapy for musculoskeletal conditions(129, 130). One of which compared an individually tailored programs with usual physical therapy for knee OA (129) and the other consisted of a two-component program (Active physical therapy + Graded activity with problem solving) vs only a one-component program (Graded activity with problem solving) for low back pain(130) (Supplementary Table 7 and Supplementary Table 12).

**Supplementary Table 15.** Summary of interventions that cost more but are less effective (N=7 interventions)

| Type of Intervention | Category | Condition | # studies | Details of interventions |
| --- | --- | --- | --- | --- |
| Behavioural  (n=5) | Mental Health(n=5) | Anxiety | 1 | Family CBT vs Individual CBT(128) ^ |
|  |  | Depression | 2 | Computerized CBT (Beating the Blues)(123) * vs UC, Classroom-based CBT vs Usual school curriculum delivered by teachers(132) ^ |
|  |  | Smoking Cessation | 2 | Internet-based multiple computer-tailored program vs UC(107) ^, Text-based computer-tailored program vs control(108) ^ |
| Exercise/Physical Therapy (n=2) | Musculoskeletal  (n=2) | Knee OA | 1 | Individually tailored or targeted exercise adherence vs Usual physical therapy(129) * |
|  |  | Chronic Low Back Pain | 1 | Active physical therapy + Graded activity with problem solving vs Graded activity with problem solving(130) ^ |

*Health system perspective

^ Societal perspective

Abbreviations; CBT=cognitive behavioural therapy, F2F=face-to-face, UC=Usual care, TAU=Treatment as usual

**Secondary Analysis: Cost Effectiveness Analysis (CEA)**

25 studies that completed a CEA and the one study a CBA. Out of the 25 studies, 11 of these CEA studies (44%) were dominant (cost less and were more effective)(131, 133-142), 12 studies (48%) cost more and were more effective(27, 143-153), and 2 studies (8%) cost less and were less effective(154, 155). The single CBA study found that all 3 of its fall prevention programs (Otago, Tai chi, and stepping on) provided net benefits(156) (Supplementary Table 11)

**References**

1. Yu C-M, Lau C-P, Chau J, McGhee SM, Kong S-L, Cheung BMY, et al. A short course of cardiac rehabilitation program is highly cost effective in improving long-term quality of life in patients with recent myocardial infarction or percutaneous coronary intervention. Archives of physical medicine and rehabilitation. 2004;85(12):1915-22.

2. Patrick DL, Ramsey SD, Spencer AC, Kinne S, Belza B, Topolski TD. Economic Evaluation of Aquatic Exercise for Persons With Osteoarthritis. Medical care. 2001;39(5):413-24.

3. Barnhofer T, Dunn BD, Strauss C, Ruths FA, Barrett B, Ryan M, et al. Mindfulness-based cognitive therapy versus treatment as usual after non-remission with NHS Talking Therapies high-intensity psychological therapy for depression: a UK-based clinical effectiveness and cost-effectiveness randomised, controlled, superiority trial. Lancet Psychiatry. 2025;12(6):433-46.

4. Goossens ME, de Kinderen RJ, Leeuw M, de Jong JR, Ruijgrok J, Evers SM, et al. Is exposure in vivo cost-effective for chronic low back pain? A trial-based economic evaluation. BMC Health Services Research. 2015;15.

5. Schweikert B, Jacobi E, Seitz R, Cziske R, Ehlert A, Knab J, et al. Effectiveness and cost-effectiveness of adding a cognitive behavioral treatment to the rehabilitation of chronic low back pain. Journal of Rheumatology. 2006;33(12).

6. Abbott JH, Wilson R, Pinto D, Chapple CM, Wright AA. Incremental clinical effectiveness and cost effectiveness of providing supervised physiotherapy in addition to usual medical care in patients with osteoarthritis of the hip or knee: 2-year results of the MOA randomised controlled trial. Osteoarthritis and Cartilage. 2019;27(3):424-34.

7. Adjetey C, Davis JC, Falck RS, Best JR, Dao E, Bennett K, et al. Economic Evaluation of Exercise or Cognitive and Social Enrichment Activities for Improved Cognition After Stroke. JAMA network open. 2023;6(11):e2345687-e.

8. Burge AT, Holland AE, McDonald CF, Abramson MJ, Hill CJ, Lee AL, et al. Home-based pulmonary rehabilitation for COPD using minimal resources: An economic analysis. Respirology. 2020;25(2).

9. Chuang LH, Soares MO, Tilbrook H, Cox H, Hewitt CE, Aplin J, et al. A pragmatic multicentered randomized controlled trial of yoga for chronic low back pain: economic evaluation. Spine. 2012;37(18).

10. Cochrane T, Davey RC, Edwards SMM. Randomised controlled trial of the cost-effectiveness of water-based therapy for lower limb osteoarthritis. Health Technology Assessment. 2005;9(19).

11. Davis JC, Hsu CL, Barha C, Jehu DA, Chan P, Ghag C, et al. Comparing the cost-effectiveness of the Otago Exercise Programme among older women and men: A secondary analysis of a randomized controlled trial. PLoS ONE [Electronic Resource]. 2022;17(4).

12. Davis JC, Khan KM, Hsu CL, Chan P, Cook WL, Dian L, et al. Action Seniors! Cost-effectiveness analysis of a secondary falls prevention strategy among community-dwelling older fallers. Journal of the American Geriatrics Society. 2020;68(9).

13. Davis JC, Marra CA, Robertson MC, Khan KM, Najafzadeh M, Ashe MC, et al. Economic evaluation of dose-response resistance training in older women: a cost-effectiveness and cost-utility analysis. Osteoporosis International. 2011;22(5).

14. Frederix I, Hansen D, Coninx K, Vandervoort PM, Vandijck D, Hens N, et al. Effect of comprehensive cardiac telerehabilitation on one-year cardiovascular rehospitalization rate, medical costs and quality of life: A cost-effectiveness analysis. European journal of preventive cardiology. 2015;23(7):674-82.

15. Groessl EJ, Lin L, Richard EL, Tally SR, Liu L. Cost-effectiveness of Yoga for Chronic Low Back Pain in Veterans. Medical Care. 2020;58.

16. Hautala AJ, Kiviniemi AM, Makikallio T, Koistinen P, Ryynanen OP, Martikainen JA, et al. Economic evaluation of exercise-based cardiac rehabilitation in patients with a recent acute coronary syndrome. Scandinavian Journal of Medicine & Science in Sports. 2017;27(11).

17. Kloek CJJ, van Dongen JM, de Bakker DH, Bossen D, Dekker J, Veenhof C. Cost-effectiveness of a blended physiotherapy intervention compared to usual physiotherapy in patients with hip and/or knee osteoarthritis: a cluster randomized controlled trial. BMC Public Health. 2018;18(1).

18. Li F, Harmer P, Eckstrom E, Fitzgerald K, Akers L, Chou L-S, et al. Cost-effectiveness of a therapeutic Tai Ji Quan fall prevention intervention for older adults at high risk of falling. The journals of gerontology Series A, Biological sciences and medical sciences. 2019;74(9):1504-10.

19. McCarthy CJ, Mills PM, Pullen R, Richardson G, Hawkins N, Roberts CR, et al. Supplementation of a home-based exercise programme with a class-based programme for people with osteoarthritis of the knees: a randomised controlled trial and health economic analysis. Health Technology Assessment (Winchester, England). 2004;8(46).

20. McCrone P, Sharpe M, Chalder T, Knapp M, Johnson AL, Goldsmith K, et al. Adaptive Pacing, Cognitive Behaviour Therapy, Graded Exercise, and Specialist Medical Care for Chronic Fatigue Syndrome: A Cost-Effectiveness Analysis. PloS one. 2012;7(8):e40808-NA.

21. Miyamoto GC, Franco KFM, van Dongen JM, dos Santos Franco YR, de Oliveira NTB, Amaral DDV, et al. Different doses of Pilates-based exercise therapy for chronic low back pain: a randomised controlled trial with economic evaluation. British journal of sports medicine. 2018;52(13):859-68.

22. Moffett JK, Torgerson DJ, Bell-Syer SEM, Jackson DA, Llewlyn-Phillips H, Farrin A, et al. Randomised controlled trial of exercise for low back pain: clinical outcomes, costs, and preferences. BMJ (Clinical research ed). 1999;319(7205):279-83.

23. Tan SS, van Linschoten RL, van Middelkoop M, Koes BW, Bierma-Zeinstra SM, Koopmanschap MA. Cost-utility of exercise therapy in adolescents and young adults suffering from the patellofemoral pain syndrome. Scandinavian Journal of Medicine & Science in Sports. 2010;20(4).

24. Vollbehr NK, Stant AD, Hoenders HJR, Bartels-Velthuis AA, Nauta MH, Castelein S, et al. Cost-effectiveness of a mindful yoga intervention added to treatment as usual for young women with major depressive disorder versus treatment as usual only: Cost-effectiveness of yoga for young women with depression. Psychiatry research. 2023;333(NA):115692-.

25. Killingmo RM, Oiestad BE, Risberg MA, Maas E, Grotle M. Cost-effectiveness of strength exercise or aerobic exercise compared with usual care for patients with knee osteoarthritis: secondary results from a multiarm randomised controlled trial in Norway. BMJ Open. 2024;14(5):e079704.

26. Lai X, Yuan Y, Wang H, Zhang R, Qiao Q, Feng X, et al. Cost-Effectiveness of Salt Substitute and Salt Supply Restriction in Eldercare Facilities. JAMA Network Open. 2024;7(2):e2355564-NA.

27. Xin Y, Davies A, McCombie L, Briggs A, Messow C-M, Grieve E, et al. Type 2 diabetes remission: economic evaluation of the DiRECT/Counterweight‐Plus weight management programme within a primary care randomized controlled trial. Diabetic medicine : a journal of the British Diabetic Association. 2019;36(8):1003-12.

28. McMillan A, Bratton DJ, Faria R, Laskawiec-Szkonter M, Griffin S, Davies RJ, et al. A multicentre randomised controlled trial and economic evaluation of continuous positive airway pressure for the treatment of obstructive sleep apnoea syndrome in older people: PREDICT. Health Technology Assessment (Winchester, England). 2015;19(40).

29. Quinnell TG, Bennett M, Jordan J, Clutterbuck-James AL, Davies MG, Smith IE, et al. A crossover randomised controlled trial of oral mandibular advancement devices for obstructive sleep apnoea-hypopnoea (TOMADO). Thorax. 2014;69(10).

30. Axelsson E, Andersson E, Ljotsson B, Hedman-Lagerlof E. Cost-effectiveness and long-term follow-up of three forms of minimal-contact cognitive behaviour therapy for severe health anxiety: Results from a randomised controlled trial. Behaviour Research and Therapy. 2018;107.

31. Hedman E, Andersson E, Lindefors N, Andersson G, Ruck C, Ljotsson B. Cost-effectiveness and long-term effectiveness of Internet-based cognitive behaviour therapy for severe health anxiety. Psychological Medicine. 2013;43(2).

32. Morriss R, Patel S, Malins S, Guo B, Higton F, James M, et al. Clinical and economic outcomes of remotely delivered cognitive behaviour therapy versus treatment as usual for repeat unscheduled care users with severe health anxiety: a multicentre randomised controlled trial. BMC Medicine. 2019;17(1).

33. Hedman E, Andersson E, Ljotsson B, Andersson G, Ruck C, Lindefors N. Cost-effectiveness of Internet-based cognitive behavior therapy vs. cognitive behavioral group therapy for social anxiety disorder: Results from a randomized controlled trial. Behaviour Research and Therapy. 2011;49(11).

34. Nordh M, Wahlund T, Jolstedt M, Sahlin H, Bjureberg J, Ahlen J, et al. Therapist-Guided Internet-Delivered Cognitive Behavioral Therapy vs Internet-Delivered Supportive Therapy for Children and Adolescents With Social Anxiety Disorder: A Randomized Clinical Trial. JAMA Psychiatry. 2021;78(7).

35. Kählke F, Buntrock C, Smit F, Berger T, Baumeister H, Ebert DD. Long-Term Outcomes and Cost-Effectiveness of an Internet-Based Self-Help Intervention for Social Anxiety Disorder in University Students: Results of a Randomized Controlled Trial. Depression and Anxiety. 2023;2023(NA):1-16.

36. Jolstedt M, Wahlund T, Lenhard F, Ljotsson B, Mataix-Cols D, Nord M, et al. Efficacy and cost-effectiveness of therapist-guided internet cognitive behavioural therapy for paediatric anxiety disorders: a single-centre, single-blind, randomised controlled trial. The Lancet Child & Adolescent Health. 2018;2(11).

37. Nordgren LB, Hedman E, Etienne J, Bodin J, Kadowaki A, Eriksson S, et al. Effectiveness and cost-effectiveness of individually tailored internet-delivered cognitive behavior therapy for anxiety disorders in a primary care population: A randomized controlled trial. Behaviour Research and Therapy. 2014;59.

38. Ginsburg GS, Pella JE, Slade E. Cost-Effectiveness Analysis of School-Based Treatments for Anxiety Disorders. The Journal of Mental Health Policy & Economics. 2022;25(3).

39. Creswell C, Violato M, Fairbanks H, White E, Parkinson M, Abitabile G, et al. Clinical outcomes and cost-effectiveness of brief guided parent-delivered cognitive behavioural therapy and solution-focused brief therapy for treatment of childhood anxiety disorders: A randomised controlled trial. The Lancet Psychiatry. 2017;4(7).

40. Witlox M, Kraaij V, Garnefski N, Bohlmeijer E, Smit F, Spinhoven P. Cost-effectiveness and cost-utility of an Acceptance and Commitment Therapy intervention vs. a Cognitive Behavioral Therapy intervention for older adults with anxiety symptoms: A randomized controlled trial. PLoS ONE Vol 17(1), 2022, ArtID e0262220. 2022;17(1).

41. Dickerson JF, Lynch FL, Leo MC, DeBar LL, Pearson J, Clarke GN. Cost-effectiveness of Cognitive Behavioral Therapy for Depressed Youth Declining Antidepressants. Pediatrics. 2018;141(2).

42. Morrell C, Warner R, Slade P, Dixon S, Walters S, Paley G, et al. Psychological interventions for postnatal depression: cluster randomised trial and economic evaluation. The PoNDER trial. Health Technology Assessment. 2009;13(37).

43. Sava FA, Yates BT, Lupu V, Szentagotai A, David D. Cost-effectiveness and cost-utility of cognitive therapy, rational emotive behavioral therapy, and fluoxetine (Prozac) in treating depression: a randomized clinical trial. Journal of Clinical Psychology. 2009;65(1).

44. Horrell L, Goldsmith KA, Tylee AT, Schmidt UH, Murphy CL, Bonin E-M, et al. One-day cognitive-behavioural therapy self-confidence workshops for people with depression: randomised controlled trial. British Journal of Psychiatry. 2014;204(3).

45. Van Lieshout RJ, Layton H, Savoy CD, Xie F, Brown JS, Huh K, et al. In-person 1-day cognitive behavioral therapy-based workshops for postpartum depression: A randomized controlled trial. Psychological Medicine. 2023.

46. Romero-Sanchiz P, Nogueira-Arjona R, Garcia-Ruiz A, Luciano JV, Garcia Campayo J, Gili M, et al. Economic evaluation of a guided and unguided internet-based CBT intervention for major depression: Results from a multi-center, three-armed randomized controlled trial conducted in primary care. PLoS ONE [Electronic Resource]. 2017;12(2).

47. Monteiro F, Antunes P, Pereira M, Canavarro MC, Fonseca A. Cost-utility of a web-based intervention to promote maternal mental health among postpartum women presenting low risk for postpartum depression. International journal of technology assessment in health care. 2022;38(1):e62-NA.

48. Zhou W, Chen Y, Wu H, Zhao H, Li Y, Shi G, et al. Cost Utility Analysis of Internet-Based Cognitive Behavioral Therapy for Major Depressive Disorder: Randomized Controlled Trial. J Med Internet Res. 2025;27:e67567.

49. Thase ME, McCrone P, Barrett MS, Eells TD, Wisniewski SR, Balasubramani GK, et al. Improving Cost-effectiveness and Access to Cognitive Behavior Therapy for Depression: Providing Remote-Ready, Computer-Assisted Psychotherapy in Times of Crisis and Beyond. Psychotherapy & Psychosomatics. 2020;89(5).

50. Wijnen BFM, Lokman S, Leone SS, Evers SMAA, Smit F. Complaint-Directed mini-Interventions for depressive symptoms: A health economic evaluation of unguided web-Based self-Help interventions based on a randomized controlled trial. Journal of medical Internet research. 2018;20(10):1-13.

51. Bower P, Byford S, Sibbald B, Ward E, King M, Lloyd M, et al. Randomised controlled trial of non-directive counselling, cognitive-behaviour therapy, and usual general practitioner care for patients with depression. II: cost effectiveness. BMJ. 2000;321(7273).

52. Lynch FL, Dickerson JF, Rozenman M, Gonzalez A, Schwartz KTG, Porta G, et al. Cost-effectiveness of Brief Behavioral Therapy for Pediatric Anxiety and Depression in Primary Care. JAMA network open. 2021;4(3):e211778-NA.

53. McCrone P, Knapp M, Proudfoot J, Ryden C, Cavanagh K, Shapiro DA, et al. Cost-effectiveness of computerised cognitive-behavioural therapy for anxiety and depression in primary care: Randomised controlled trial. The British Journal of Psychiatry. 2004;185(1).

54. Strauss C, Bibby-Jones AM, Jones F, Byford S, Heslin M, Parry G, et al. Clinical Effectiveness and Cost-Effectiveness of Supported Mindfulness-Based Cognitive Therapy Self-help Compared With Supported Cognitive Behavioral Therapy Self-help for Adults Experiencing Depression: The Low-Intensity Guided Help Through Mindfulness (LIGHTMind) Randomized Clinical Trial. JAMA Psychiatry. 2023;80(5).

55. Baka A, van der Zweerde T, Lancee J, Bosmans JE, van Straten A. Cost-effectiveness of Guided Internet-Delivered Cognitive Behavioral Therapy in Comparison with Care-as-Usual for Patients with Insomnia in General Practice. Behavioral sleep medicine. 2021;20(2):1-16.

56. Buntrock C, Lehr D, Smit F, Horvath H, Berking M, Spiegelhalder K, et al. Guided Internet-Based Cognitive Behavioral Therapy for Insomnia: Health-Economic Evaluation From the Societal and Public Health Care Perspective Alongside a Randomized Controlled Trial. Journal of Medical Internet Research. 2021;23(5).

57. De Bruin EJ, van Steensel FJ, Meijer AM. Cost-Effectiveness of Group and Internet Cognitive Behavioral Therapy for Insomnia in Adolescents: Results from a Randomized Controlled Trial. Sleep. 2016;39(8).

58. Brettschneider C, Gensichen J, Hiller TS, Breitbart J, Schumacher U, Lukaschek K, et al. Cost-effectiveness of Practice Team-Supported Exposure Training for Panic Disorder and Agoraphobia in Primary Care: a Cluster-Randomized Trial. JGIM: Journal of General Internal Medicine. 2020;35(4).

59. Le QA, Doctor JN, Zoellner LA, Feeny NC. Cost-effectiveness of prolonged exposure therapy versus pharmacotherapy and treatment choice in posttraumatic stress disorder (the Optimizing PTSD Treatment Trial): a doubly randomized preference trial. Journal of Clinical Psychiatry. 2014;75(3).

60. Xin Y, Davies A, Briggs A, McCombie L, Messow CM, Grieve E, et al. Type 2 diabetes remission: 2 year within-trial and lifetime-horizon cost-effectiveness of the Diabetes Remission Clinical Trial (DiRECT)/Counterweight-Plus weight management programme. Diabetologia. 2020;63(10):2112-22.

61. Alfonso-Rosa RM, del Pozo-Cruz J, del Pozo-Cruz B, Sañudo B, Abellan-Perpiñan JM. Cost-utility analysis of a 12-week whole-body vibration based treatment for people with type 2 diabetes: reanalysis of a RCT in a primary care context. Public health. 2015;129(7):993-5.

62. Ambrens M, Schooten KSv, Lung T, Clemson L, Close JCT, Howard K, et al. Economic evaluation of the e-Health StandingTall balance exercise programme for fall prevention in people aged 70 years and over. Age & Ageing. 2022;51(6).

63. Bailly L, Mosse P, Diagana S, Fournier M, d'Arripe-Longueville F, Diagana O, et al. "As du Coeur" study: a randomized controlled trial on quality of life impact and cost effectiveness of a physical activity program in patients with cardiovascular disease. BMC Cardiovascular Disorders. 2018;18(1).

64. Barton G, Sach T, Jenkinson C, Doherty M, Avery AJ, Muir K. Lifestyle interventions for knee pain in overweight and obese adults aged ≥45: economic evaluation of randomised controlled trial. BMJ (Clinical research ed). 2009;339(7721):610-2.

65. Bennell KL, Ahamed Y, Jull G, Bryant C, Hunt MA, Forbes AB, et al. Physical Therapist-Delivered Pain Coping Skills Training and Exercise for Knee Osteoarthritis: Randomized Controlled Trial. Arthritis care & research. 2016;68(5).

66. Chalder M, Wiles NJ, Campbell J, Hollinghurst S, Searle A, Haase AM, et al. A pragmatic randomised controlled trial to evaluate the cost-effectiveness of a physical activity intervention as a treatment for depression: the treating depression with physical activity (TREAD) trial. Health technology assessment (Winchester, England). 2012;16(10):10-164, iii.

67. Chew DS, Li Y, Zeitouni M, Whellan DJ, Kitzman DW, Mentz RJ, et al. Economic Outcomes of Rehabilitation Therapy in Older Patients With Acute Heart Failure in the REHAB-HF Trial: A Secondary Analysis of a Randomized Clinical Trial. JAMA cardiology. 2022;7(2):140-NA.

68. Coyle D, Coyle K, Kenny GP, Boule NG, Wells GA, Fortier M, et al. Cost-effectiveness of exercise programs in type 2 diabetes. International Journal of Technology Assessment in Health Care. 2012;28(3).

69. Eriksson MK, Hagberg L, Lindholm L, Malmgren-Olsson EB, Osterlind J, Eliasson M. Quality of life and cost-effectiveness of a 3-year trial of lifestyle intervention in primary health care. Archives of Internal Medicine. 2010;170(16).

70. Gillespie P, O'Shea E, Casey D, Murphy K, Devane D, Cooney A, et al. The cost-effectiveness of a structured education pulmonary rehabilitation programme for chronic obstructive pulmonary disease in primary care: the PRINCE cluster randomised trial. BMJ open. 2013;3(11):e003479-NA.

71. Gusi N, Reyes MC, Gonzalez-Guerrero JL, Herrera E, Garcia JM. Cost-utility of a walking programme for moderately depressed, obese, or overweight elderly women in primary care: a randomised controlled trial. BMC Public Health. 2008;8.

72. Hochheim M, Ramm P, Wunderlich M, Amelung V. Cost-effectiveness analysis of a chronic back pain multidisciplinary biopsychosocial rehabilitation (MBR) compared to standard care for privately insured in Germany. BMC health services research. 2021;21(1):1362-NA.

73. Hornby TG, Rafferty MR, Pinto D, French D, Jordan N. Cost-Effectiveness of High-intensity Training vs Conventional Therapy for Individuals With Subacute Stroke. Archives of Physical Medicine & Rehabilitation. 2022;103(7).

74. Johnson RE, Jones GT, Wiles NJ, Chaddock C, Potter RG, Roberts C, et al. Active exercise, education, and cognitive behavioral therapy for persistent disabling low back pain: a randomized controlled trial. Spine. 2007;32(15).

75. Koppenaal T, van Dongen JM, Kloek CJ, Arensman RM, Veenhof C, Pisters MF, et al. Effectiveness and Cost-Effectiveness of a Stratified Blended Physiotherapy Intervention Compared With Face-to-Face Physiotherapy in Patients With Nonspecific Low Back Pain: Cluster Randomized Controlled Trial. Journal of medical Internet research. 2023;25(NA):e43034-e.

76. Maru S, Mudge AM, Suna JM, Scuffham PA. One-year cost-effectiveness of supervised center-based exercise training in addition to a post-discharge disease management program for patients recently hospitalized with acute heart failure: The EJECTION-HF study. Heart & Lung. 2019;48(5).

77. Matchar DB, Eom K, Duncan PW, Lee M, Sim R, Sivapragasam NR, et al. A Cost-Effectiveness Analysis of a Randomized Control Trial of a Tailored, Multifactorial Program to Prevent Falls Among the Community-Dwelling Elderly. Archives of Physical Medicine & Rehabilitation. 2019;100(1).

78. Murphy SM, Edwards RT, Williams N, Raisanen L, Moore G, Linck P, et al. An evaluation of the effectiveness and cost effectiveness of the National Exercise Referral Scheme in Wales, UK: a randomised controlled trial of a public health policy initiative...[corrected][published erratum appears in J EPIDEMIOL COMMUNITY HEALTH 2012 Nov; p.1082]. Journal of Epidemiology & Community Health. 2012;66(8).

79. Oppong R, Jowett S, Nicholls E, Whitehurst DGT, Hill SL, Hammond A, et al. Joint protection and hand exercises for hand osteoarthritis: an economic evaluation comparing methods for the analysis of factorial trials. Rheumatology (Oxford, England). 2014;54(5):876-83.

80. Pinto D, Robertson MC, Abbott JH, Hansen P, Campbell AJ. Manual therapy, exercise therapy, or both, in addition to usual care, for osteoarthritis of the hip or knee. 2: economic evaluation alongside a randomized controlled trial. Osteoarthritis & Cartilage. 2013;21(10).

81. Reed SD, Whellan DJ, Li Y, Friedman JY, Ellis SJ, Pina IL, et al. Economic evaluation of the HF-ACTION (Heart Failure: A Controlled Trial Investigating Outcomes of Exercise Training) randomized controlled trial: an exercise training study of patients with chronic heart failure. Circulation Cardiovascular Quality & Outcomes. 2010;3(4).

82. Rhon DI, Kim M, Asche CV, Allison SC, Allen CS, Deyle GD. Cost-effectiveness of Physical Therapy vs Intra-articular Glucocorticoid Injection for Knee Osteoarthritis: A Secondary Analysis From a Randomized Clinical Trial. JAMA network open. 2022;5(1):e2142709-e.

83. Stanmore E, Mavroeidi A, de Jong LD, Skelton DA, Sutton CJ, Benedetto V, et al. The effectiveness and cost-effectiveness of strength and balance Exergames to reduce falls risk for people aged 55 years and older in UK assisted living facilities: a multi-centre, cluster randomised controlled trial. BMC medicine. 2019;17(1):49-NA.

84. Turner DA, Carter T, Sach T, Guo B, Callaghan P. Cost-effectiveness of a preferred intensity exercise programme for young people with depression compared with treatment as usual: An economic evaluation alongside a clinical trial in the UK. BMJ open. 2017;7(11):e016211-NA.

85. Ulfsdottir H, Bäck M, Cider Å, Jivegård L, Sandberg A, Nordanstig J, et al. Cost-Effectiveness of Exercise Therapy in Patients with Intermittent Claudication-A Comparison of Supervised Exercise, Home-Based Structured Exercise, and Walk Advice from the SUNFIT Trial. Journal of clinical medicine. 2023;12(16):5277-.

86. van Asselt AD, Nicolai SP, Joore MA, Prins MH, Teijink JA. Cost-effectiveness of exercise therapy in patients with intermittent claudication: supervised exercise therapy versus a 'go home and walk' advice. European Journal of Vascular & Endovascular Surgery. 2011;41(1).

87. Williamson E, Sanchez-Santos MT, Marian IR, Maredza M, Srikesavan C, Garrett A, et al. Improving the understanding and management of back pain in older adults: the BOOST research programme including RCT and OPAL cohort. Programme Grants for Applied Research. 2023;NA(NA):1-89.

88. Biesheuvel-Leliefeld KEM, Bosmans JE, Dijkstra-Kersten SMA, Smit F, Bockting CLH, van Schaik DJF, et al. A supported self-help for recurrent depression in primary care; An economic evaluation alongside a multi-center randomised controlled trial. PLoS ONE [Electronic Resource]. 2018;13(12).

89. Buntrock C, Berking M, Smit F, Lehr D, Nobis S, Riper H, et al. Preventing Depression in Adults With Subthreshold Depression: Health-Economic Evaluation Alongside a Pragmatic Randomized Controlled Trial of a Web-Based Intervention. Journal of Medical Internet Research. 2017;19(1).

90. Chapdelaine A, Vasiliadis H-M, Provencher MD, Norton PJ, Roberge P. Cost-effectiveness of transdiagnostic group cognitive behavioural therapy for anxiety disorders v. treatment as usual: economic evaluation of a pragmatic randomized controlled trial over an 8-month time horizon using self-reported data. Psychological medicine. 2023;53(14):1-6582.

91. Dams J, Kronmuller K-T, Leibing E, Steil R, Henningsen P, Leichsenring F, et al. Direct costs of social phobia in adolescents and cost-effectiveness of psychotherapy. Psychiatrische Praxis. 2019;46(3).

92. de Jonge M, Blankers M, Bockting CLH, van Dijk MK, Kikkert MJ, Dekker JJM. Economic evaluation of preventive cognitive therapy versus care as usual in cognitive behavioral therapy responders. Frontiers in psychiatry. 2024;14(NA):1134071-NA.

93. Dear BF, Zou JB, Ali S, Lorian CN, Johnston L, Sheehan J, et al. Clinical and cost-effectiveness of therapist-guided internet-delivered cognitive behavior therapy for older adults with symptoms of anxiety: A randomized controlled trial. Behavior Therapy. 2015;46(2).

94. Egger N, Konnopka A, Beutel ME, Herpertz S, Hiller W, Hoyer J, et al. Short-term cost-effectiveness of psychodynamic therapy and cognitive-behavioral therapy in social anxiety disorder: Results from the SOPHO-NET trial. Journal of Affective Disorders. 2015;180.

95. Hakkaart-Van Roijen L, Van Straten A, Al M, Rutten F, Donker M. Cost-utility of brief psychological treatment for depression and anxiety. The British Journal of Psychiatry. 2006;188(4).

96. Hedman E, Andersson E, Ljotsson B, Axelsson E, Lekander M. Cost effectiveness of internet-based cognitive behaviour therapy and behavioural stress management for severe health anxiety. BMJ Open. 2016;6(4).

97. Hollinghurst S, Carroll FE, Abel A, Campbell J, Garland A, Jerrom B, et al. Cost-effectiveness of cognitive-behavioural therapy as an adjunct to pharmacotherapy for treatment-resistant depression in primary care: economic evaluation of the CoBalT Trial. British Journal of Psychiatry. 2014;204(1).

98. Hollinghurst S, Peters TJ, Kaur S, Wiles N, Lewis G, Kessler D. Cost-effectiveness of therapist-delivered online cognitive-behavioural therapy for depression: randomised controlled trial. British Journal of Psychiatry. 2010;197(4).

99. Klein NS, Bockting CL, Wijnen B, Kok GD, van Valen E, Riper H, et al. Economic Evaluation of an Internet-Based Preventive Cognitive Therapy With Minimal Therapist Support for Recurrent Depression: Randomized Controlled Trial. Journal of Medical Internet Research. 2018;20(11).

100. Kolovos S, Kenter RMF, Bosmans JE, Beekman ATF, Cuijpers P, Kok RN, et al. Economic evaluation of Internet-based problem-solving guided self-help treatment in comparison with enhanced usual care for depressed outpatients waiting for face-to-face treatment: A randomized controlled trial. Journal of affective disorders. 2016;200(NA):284-92.

101. Kraepelien M, Mattsson S, Hedman-Lagerlöf E, Petersson IF, Forsell Y, Lindefors N, et al. Cost-effectiveness of internet-based cognitive-behavioural therapy and physical exercise for depression. BJPsych open. 2018;4(4):265-73.

102. Lamb SE, Lall R, Hansen Z, Castelnuovo E, Withers EJ, Nichols V, et al. A multicentred randomised controlled trial of a primary care-based cognitive behavioural programme for low back pain. The Back Skills Training (BeST) trial. Health Technology Assessment (Winchester, England). 2010;14(41).

103. Lynch FL, Hornbrook M, Clarke GN, Perrin N, Polen MR, O'Connor E, et al. Cost-effectiveness of an intervention to prevent depression in at-risk teens. Archives of General Psychiatry. 2005;62(11).

104. Meuldijk D, Carlier IV, van Vliet IM, van Hemert AM, Zitman FG, van den Akker-van Marle ME. Economic Evaluation of Concise Cognitive Behavioural Therapy and/or Pharmacotherapy for Depressive and Anxiety Disorders. The Journal of Mental Health Policy & Economics. 2015;18(4).

105. Mourad G, Lundgren J, Andersson G, Husberg M, Johansson P. Cost-effectiveness of internet-delivered cognitive behavioural therapy in patients with cardiovascular disease and depressive symptoms: secondary analysis of an RCT. BMJ Open. 2022;12(4).

106. Revicki DA, Siddique J, Frank L, Chung JY, Green BL, Krupnick J, et al. Cost-effectiveness of evidence-based pharmacotherapy or cognitive behavior therapy compared with community referral for major depression in predominantly low-income minority women. Archives of General Psychiatry. 2005;62(8).

107. Smit ES, Evers SMAA, de Vries H, Hoving C. Cost-effectiveness and cost-utility of Internet-based computer tailoring for smoking cessation. Journal of medical Internet research. 2013;15(3):55-70.

108. Stanczyk NE, Smit ES, Schulz DN, de Vries H, Bolman C, Muris JWM, et al. An Economic Evaluation of a Video- and Text-Based Computer-Tailored Intervention for Smoking Cessation: A Cost-Effectiveness and Cost-Utility Analysis of a Randomized Controlled Trial. PloS one. 2014;9(10):e110117-NA.

109. Titov N, Dear BF, Ali S, Zou JB, Lorian CN, Johnston L, et al. Clinical and cost-effectiveness of therapist-guided internet-delivered cognitive behavior therapy for older adults with symptoms of depression: a randomized controlled trial. Behavior Therapy. 2015;46(2).

110. Warmerdam L, Smit F, van Straten A, Riper H, Cuijpers P. Cost-utility and cost-effectiveness of internet-based treatment for adults with depressive symptoms: randomized trial. Journal of Medical Internet Research. 2010;12(5).

111. Ali S, Alemu FW, Owen J, Eells TD, Antle B, Lee JT, et al. Cost-Effectiveness of Computer-Assisted Cognitive Behavioral Therapy for Depression Among Adults in Primary Care. JAMA Netw Open. 2024;7(11):e2444599.

112. Kyle SD, Siriwardena AN, Espie CA, Yang Y, Petrou S, Ogburn E, et al. Clinical and cost-effectiveness of nurse-delivered sleep restriction therapy for insomnia in primary care (HABIT): a pragmatic, superiority, open-label, randomised controlled trial. Lancet. 2023;402(10406):975-87.

113. Adams J, Barratt P, Rombach I, Arden N, Barbosa Boucas S, Bradley S, et al. The clinical and cost effectiveness of splints for thumb base osteoarthritis: a randomized controlled clinical trial. Rheumatology. 2021;60(6).

114. de Vries GE, Hoekema A, Vermeulen KM, Claessen J, Jacobs W, van der Maten J, et al. Clinical- and Cost-Effectiveness of a Mandibular Advancement Device Versus Continuous Positive Airway Pressure in Moderate Obstructive Sleep Apnea. Journal of Clinical Sleep Medicine. 2019;15(10).

115. Gueugnon M, Fournel I, Soilly AL, Diaz A, Baulot E, Bussiere C, et al. Effectiveness, safety, and cost-utility of a knee brace in medial knee osteoarthritis: the ERGONOMIE randomized controlled trial. Osteoarthritis & Cartilage. 2021;29(4).

116. Li K-C, Huang L, Tian M, Di Tanna GL, Yu J, Zhang X, et al. Cost-Effectiveness of a Household Salt Substitution Intervention: Findings From 20 995 Participants of the Salt Substitute and Stroke Study. Circulation. 2022;145(20).

117. Bradshaw LE, Wyatt LA, Brown SJ, Haines RH, Montgomery AA, Perkin MR, et al. Emollient application from birth to prevent eczema in high-risk children: the BEEP RCT. Health Technol Assess. 2024;28(29):1-116.

118. Bonin E-M, Beecham J, Swift N, Raikundalia S, Brown JS. Psycho-educational CBT-insomnia workshops in the community. A cost-effectiveness analysis alongside a randomised controlled trial. Behaviour Research and Therapy. 2014;55.

119. Morgan K, Dixon, S, Mathers, N, Thompson, J, Tomeny M. Psychological treatment for insomnia in the regulation of long-term hypotic drug use. Health Techology Assessment. 2004;8(8).

120. Axelsson E, Andersson E, Ljotsson B, Bjorkander D, Hedman-Lagerlof M, Hedman-Lagerlof E. Effect of internet vs face-to-face cognitive behavior therapy for health anxiety: A randomized noninferiority clinical trial. JAMA Psychiatry. 2020;77(9).

121. Creswell C, Taylor L, Giles S, Howitt S, Radley L, Whitaker E, et al. Digitally augmented, parent-led CBT versus treatment as usual for child anxiety problems in child mental health services in England and Northern Ireland: a pragmatic, non-inferiority, clinical effectiveness and cost-effectiveness randomised controlled trial. The lancet Psychiatry. 2024;11(3):193-209.

122. Domino ME, Foster EM, Vitiello B, Kratochvil CJ, Burns BJ, Silva SG, et al. Relative cost-effectiveness of treatments for adolescent depression: 36-week results from the TADS randomized trial. Journal of the American Academy of Child & Adolescent Psychiatry. 2009;48(7).

123. Duarte A, Walker S, Littlewood E, Brabyn S, Hewitt C, Gilbody S, et al. Cost-effectiveness of computerized cognitive-behavioural therapy for the treatment of depression in primary care: findings from the Randomised Evaluation of the Effectiveness and Acceptability of Computerised Therapy (REEACT) trial. Psychological Medicine. 2017;47(10).

124. El Alaoui S, Hedman-Lagerlof E, Ljotsson B, Lindefors N. Does internet-based cognitive behaviour therapy reduce healthcare costs and resource use in treatment of social anxiety disorder? A cost-minimisation analysis conducted alongside a randomised controlled trial. BMJ Open. 2017;7(9).

125. Gerhards SA, de Graaf LE, Jacobs LE, Severens JL, Huibers MJ, Arntz A, et al. Economic evaluation of online computerised cognitive-behavioural therapy without support for depression in primary care: randomised trial. British Journal of Psychiatry. 2010;196(4).

126. Holst A, Bjorkelund C, Metsini A, Madsen JH, Hange D, Petersson EL, et al. Cost-effectiveness analysis of internet-mediated cognitive behavioural therapy for depression in the primary care setting: results based on a controlled trial. BMJ Open. 2018;8(6).

127. van Reijen NS, van Dieren S, Frans FA, Reekers JA, Metz R, Buscher H, et al. Cost Effectiveness of Endovascular Revascularisation vs. Exercise Therapy for Intermittent Claudication Due to Iliac Artery Obstruction. European Journal of Vascular & Endovascular Surgery. 2022;63(3).

128. Bodden DH, Dirksen CD, Bogels SM, Nauta MH, De Haan E, Ringrose J, et al. Costs and cost-effectiveness of family CBT versus individual CBT in clinically anxious children. Clinical Child Psychology and Psychiatry. 2008;13(4).

129. Kigozi J, Jowett S, Nicholls E, Tooth S, Hay EM, Foster NE, et al. Cost-utility analysis of interventions to improve effectiveness of exercise therapy for adults with knee osteoarthritis: the BEEP trial. Rheumatology advances in practice. 2018;2(2):rky018-NA.

130. Smeets RJ, Severens JL, Beelen S, Vlaeyen JW, Knottnerus JA. More is not always better: cost-effectiveness analysis of combined, single behavioral and single physical rehabilitation programs for chronic low back pain. European Journal of Pain. 2009;13(1).

131. Smit F, Willemse G, Koopmanschap M, Onrust S, Cuijpers P, Beekman A. Cost-effectiveness of preventing depression in primary care patients: randomised trial. British Journal of Psychiatry. 2006;188.

132. Stallard P, Phillips R, Montgomery A, Spears M, Anderson R, Taylor J, et al. A cluster randomised controlled trial to determine the clinical effectiveness and cost-effectiveness of classroom-based cognitive-behavioural therapy (CBT) in reducing symptoms of depression in high-risk adolescents. Health Technology Assessment. 2013;17(32).

133. Aranda-Reneo I, Albornos-Muñoz L, Rich-Ruiz M, Cidoncha-Moreno MÁ, Pastor-López Á, Moreno-Casbas T. Cost-Effectiveness of an Exercise Programme That Provided Group or Individual Training to Reduce the Fall Risk in Healthy Community-Dwelling People Aged 65-80: A Secondary Data Analysis. Healthcare (Basel, Switzerland). 2021;9(6):714-NA.

134. Bergstrom J, Andersson G, Ljotsson B, Ruck C, Andreewitch S, Karlsson A, et al. Internet-versus group-administered cognitive behaviour therapy for panic disorder in a psychiatric setting: A randomised trial. BMC Psychiatry Vol 10 2010, ArtID 54. 2010;10.

135. de Boer MJ, Versteegen GJ, Vermeulen KM, Sanderman R, Struys MM. A randomized controlled trial of an Internet-based cognitive-behavioural intervention for non-specific chronic pain: an effectiveness and cost-effectiveness study. European Journal of Pain. 2014;18(10).

136. Hupperets MDW, Verhagen E, Heymans MW, Bosmans JE, van Tulder MW, van Mechelen W. Potential Savings of a Program to Prevent Ankle Sprain Recurrence Economic Evaluation of a Randomized Controlled Trial. The American journal of sports medicine. 2010;38(11):2194-200.

137. Janssen KW, Hendriks MR, van Mechelen W, Verhagen E. The Cost-Effectiveness of Measures to Prevent Recurrent Ankle Sprains: Results of a 3-Arm Randomized Controlled Trial. American Journal of Sports Medicine. 2014;42(7).

138. Patil R, Kolu P, Raitanen J, Valvanne J, Kannus P, Karinkanta S, et al. Cost-effectiveness of vitamin D supplementation and exercise in preventing injurious falls among older home-dwelling women: findings from an RCT. Osteoporosis international : a journal established as result of cooperation between the European Foundation for Osteoporosis and the National Osteoporosis Foundation of the USA. 2015;27(1):193-201.

139. Rizzo JA, Baker DI, McAvay G, Tinetti ME. The cost-effectiveness of a multifactorial targeted prevention program for falls among community elderly persons. Medical care. 1996;34(9):954-69.

140. Simpson SA, Corney R, Fitzgerald P, Beecham J. A randomized controlled trial to evaluate the effectiveness and cost-effectiveness of psychodynamic counselling for general practice patients with chronic depression. Psychological medicine. 2003;33(2):229-39.

141. Thiart H, Ebert DD, Lehr D, Nobis S, Buntrock C, Berking M, et al. Internet-Based Cognitive Behavioral Therapy for Insomnia: A Health Economic Evaluation. Sleep. 2016;39(10).

142. van Apeldoorn F, Stant A, van Hout W, Mersch P, den Boer J. Cost-effectiveness of CBT, SSRI, and CBT+SSRI in the treatment for panic disorder. Acta Psychiatrica Scandinavica. 2014;129(4).

143. Boggs JM, Ritzwoller DP, Beck A, Dimidjian S, Segal ZV. Cost-Effectiveness of a Web-Based Program for Residual Depressive Symptoms: Mindful Mood Balance. Psychiatric services (Washington, DC). 2021;73(2):158-64.

144. Hewitt J, Saing S, Goodall S, Henwood T, Clemson L, Refshauge K. An economic evaluation of the SUNBEAM programme: A falls-prevention randomized controlled trial in residential aged care. Clinical Rehabilitation. 2019;33(3).

145. Holman AJ, Serfaty MA, Leurent BE, King MB. Cost-effectiveness of cognitive behaviour therapy versus talking and usual care for depressed older people in primary care. BMC Health Services Research. 2011;11.

146. Kafali N, Cook B, Canino G, Alegria M. Cost-effectiveness of a randomized trial to treat depression among Latinos. The Journal of Mental Health Policy & Economics. 2014;17(2).

147. Petrou S, Cooper P, Murray L, Davidson LL. Cost-effectiveness of a preventive counseling and support package for postnatal depression. International Journal of Technology Assessment in Health Care. 2006;22(4).

148. Poirier-Bisson J, Marchand A, Pelland M-E, Lessard M-J, Dupuis G, Fleet R, et al. Incremental cost-effectiveness of pharmacotherapy and two brief cognitive-behavioral therapies compared with usual care for panic disorder and noncardiac chest pain. Journal of Nervous and Mental Disease. 2013;201(9).

149. Robertson MC, Devlin N, Gardner MM, Campbell AJ. Effectiveness and economic evaluation of a nurse delivered home exercise programme to prevent falls. 1: Randomised controlled trial. BMJ. 2001;322(7288).

150. Robertson MC, Devlin N, Scuffham P, Gardner MM, Buchner DM, Campbell AJ. Economic evaluation of a community based exercise programme to prevent falls. Journal of Epidemiology & Community Health. 2001;55(8).

151. Scott J, Palmer S, Paykel E, Teasdale J, Hayhurst H. Use of cognitive therapy for relapse prevention in chronic depression. Cost-effectiveness study. Br J Psychiatry. 2003;182:221-7.

152. Slade EP, Gottlieb JD, Lu W, Yanos PT, Rosenberg S, Silverstein SM, et al. Cost-Effectiveness of a PTSD Intervention Tailored for Individuals With Severe Mental Illness. Psychiatric Services. 2017;68(12).

153. Thomas KS, Miller P, Doherty M, Muir KR, Jones AC, O'Reilly SC. Cost effectiveness of a two-year home exercise program for the treatment of knee pain. Arthritis & Rheumatism. 2005;53(3).

154. King M, Sibbald B, Ward E, Bower P, Lloyd M, Gabbay M, et al. Randomised controlled trial of non-directive counselling, cognitive-behaviour therapy and usual general practitioner care in the management of depression as well as mixed anxiety and depression in primary care. Health Technology Assessment (Winchester, England). 2000;4(19).

155. Roberge P, Marchand A, Reinharz D, Savard P. Cognitive-behavioral treatment for panic disorder with agoraphobia: A randomized, controlled trial and cost-effectiveness analysis. Behavior Modification. 2008;32(3).

156. Carande-Kulis V, Stevens JA, Florence CS, Beattie BL, Arias I. A cost-benefit analysis of three older adult fall prevention interventions. Journal of Safety Research. 2015;52.
